# Impact of Information Leakage in Platform Trials With Survival Endpoints on Type I Error Control

**DOI:** 10.1002/pst.70106

**Published:** 2026-06-25

**Authors:** Quynh Nguyen, Martin Posch, Benjamin Hofner, Franz König

**Affiliations:** ^1^ Section Data Science and Methods Paul‐Ehrlich‐Institut Langen Germany; ^2^ Department of Medical Informatics Biometry, and Epidemiology, Friedrich‐Alexander‐Universität Erlangen‐Nürnberg Erlangen Germany; ^3^ Institute of Medical Statistics, Center for Medical Data Science Medical University of Vienna Austria

**Keywords:** common control group, conditional error, maximum Type I error, time‐to‐event

## Abstract

Platform trials evaluate multiple treatments within a single trial infrastructure. Such designs have gained a lot of attraction in clinical research. If information gained from platform trials should provide confirmatory evidence for regulatory decisions, control of the Type I error rate is key. One critical issue is information leakage, for example, if any information of the ongoing trial is available, especially if it may impact the further conduct of treatments still in the platform trial and bias their results. This paper evaluates the potential impact of information leakage on the control of the Type I error rate in platform trials with time‐to‐event endpoints such as overall survival. We explore different strategies how information on the treatment effect of an still ongoing treatment could be obtained if a pre‐planned analysis for another arm is conducted. This (leaked) information might be used to decide whether to continue the other arm as planned or conduct its final analysis immediately. By means of clinical trial simulations we evaluate the impact of different levels of information leakage on the Type I error rate. We show how the conditional error principle can be applied to estimate worst case Type I error rate inflation for the different forms of information leakage. We do not aim to quantify the exact maximum Type I error rate inflation but rather to raise awareness of the potential risk for estimation of comparative results. Finally, we discuss the regulatory implications of information leakage and propose strategies to mitigate these risks.

## 1 Introduction

Platform trials offer a variety of flexible trial design features such as the evaluation of multiple treatment arms or the use of shared controls. Additional treatment arms can be added to an ongoing platform trial. In addition, adaptive randomisation or the use of non‐concurrent controls are commonly discussed for platform trials [1, 2]. Prominent examples of such flexible platform trials are the ISPY‐2 trial [3], the STAMPEDE trial [4] or the RECOVERY trial [5]. While the flexibility in trial design allows for faster evaluation of new interventions and provides potential logistical and operational advantages within a shared framework, this comes at the price of an increased complexity of the trial. Operational challenges such as the submission of clinical trials for authorisation, or statistical challenges such as multiplicity control, use of non‐concurrent controls or in general the use of adaptive features are only a few aspects which add to the complexity. Koenig et al. [1] and Nguyen et al. [2] provide a summary of operational, regulatory and methodological challenges in platform trials. In the latter, corresponding references to regulatory guidances from the European Medicines Agency (EMA) and the US Food and Drug Administration (FDA) are included where possible. In Europe, a Recommendation Paper on the Initiation and Conduct of Complex Clinical Trials was issued [6] mainly focusing on operational aspects, and the EMA published a Questions and Answers (EMA Q&A) document on Complex Clinical Trials [7], which primarily covers operational aspects but also discusses some methodological aspects. A concept paper discussing the need for a reflection paper on methodological aspects of platform trials was published in 2022 [8]. The FDA recently provided a draft guidance on master protocols [9], which was open for comments [10]. FDA's guidance on interacting with the FDA on complex trials provides aspects that should be considered when proposing complex trials [11] such as the statistical considerations or the decisions allocated to the data monitoring committees.

While methodological aspects on the need for multiplicity control [12, 13, 14], use of non‐concurrent controls [15, 16] or use of simulations [17] are discussed in the literature, sufficient regulatory guidance covering all complex aspects is currently still limited but slowly increasing. However, one aspect is almost always mentioned in the guidelines: maintaining data and trial integrity as one of the key aspects for platform trials [6, 7, 8, 11]. In particular, EMA's Q&A document on Complex Clinical Trials [8] identifies conflicting aspects between the need for transparency and the need for trial integrity when treatment arms are closed and consequently trial results become available. On the one hand, under the framework of the EU clinical trial regulation [18], timely submission and publication of summary results is mandated. On the other hand, publication of comparative results for one treatment against a shared control while other treatments are still ongoing in the trial can potentially compromise the blinding. In particular, information revealed about the shared control can (unintentionally) affect the further trial conduct or lead to data‐driven changes to the trial and consequently pose a risk to trial integrity. Thus, in the EMA Q&A document [8] an exception allows for a delayed release of results, if the publication of results might interfere with trial integrity. However, in an extreme scenario when platform trials potentially run perpetually, publication of results only at the very end of a platform trial would not be acceptable [1]. A balance between transparency and trial integrity should therefore be sought for and the strategy should be pre‐specified in the protocol [8].

Data‐driven changes in clinical trials have been discussed in the literature in the context of group‐sequential and adaptive designs [19]. If changes of the trials are motivated by external factors independent of the trial, then this should have no impact on the Type I error rate and therefore no adjustment of the significance level is needed. However, Proschan and Hunsberger [20] showed that sample size adaptations after observing interim trial data can result in a Type I error rate inflation. Proposing the conditional error principle, the authors obtained the maximum Type I error rate by assuming that for each interim outcome, the experimenter would choose the sample size adaptation that maximises the conditional Type I error rate and consequently the overall (unconditional) Type I error rate. Following the conditional error principle [20, 21], multiple authors have investigated the maximum Type I error rate for various trial adaptations based on interim data: Graf and Bauer [22] determined the maximum Type I error rate when sample size and allocation ratio are adapted based on interim data. Graf et al. [23] searched for the worst case scenario in multi‐arm trials comparing treatments to a shared control arm where treatment selection and sample size reassessments are performed based on interim data. Żebrowska, Posch and Magirr [24] investigated the maximum Type I error rate when sample size reassessment is based on interim data not only from the primary but also from a secondary endpoint. For these cases above, it is assumed that interim results for the treatment arms are available for any decision or adaptation. Thus, in general a (planned) interim analysis of this arm should precede in order to obtain interim data. In contrast, in a platform trial information on a specific arm might be released prior to a planned analysis of that arm. Supposedly independent analyses from one arm can release information for other arms when shared controls are used. In time‐to‐event platform trials, where analyses are event‐driven, the number of events is constantly monitored providing even more information. Combining information, comparative results for treatments still in the trial can potentially be recalculated or estimated, and in case of favourable results, an ad‐hoc efficacy stop could be initiated claiming that it was triggered by external information. The experimenter could deny any usage of internal information for the decision of stopping for efficacy as no data from (interim) analyses for that arm was used, while in fact information from the platform trial was used (results from other arms, and from event monitoring).

In a simulation study, we investigate the maximum Type I error rate in an event‐driven platform trial when one arm releases comparative results while other arms are still in the trial and combine this with information from event monitoring. We will focus on the Type I error rate for the treatment arm still in the trial. We investigate the impact on the Type I error rate if the standard test statistics are used despite performing design adaptations, that is, using a log‐rank test at level α. Importantly, we do not aim to quantify the exact maximum Type I error rate inflation or to provide guidance on how to (‘optimally’) use information in order to maximise the Type I error, but rather to investigate whether information leakage is an issue in platform trials. Furthermore, we limit the number of treatment arms in our simulations to two treatments, as with an increasing number of treatment arms the possibilities for information leakage increase exponentially with all potential combinations of leakage time points and leakage data. Nevertheless, using two treatment arms will still provide insights to the impact of information leakage in platform trials and can raise awareness of the potential issue.

Note that there are adaptive tests based on the conditional error principle which are known to control the Type I error rate [25, 26] depending on the interim data actually utilised [27]. These will not be considered in the simulation study, as the aim is to understand the potential Type I error inflation in case the use of internal information is concealed. Furthermore, the Type I error control across treatment arms in a platform study (see e.g., [13, 14, 28]) is out of scope here as well.

In the following section, we will introduce the notation, including the proposed trial design and four potential scenarios of information leakage. In Section 3, we present our simulation study, which is followed by the simulation results in Section 4. For each of the four scenarios of information leakage, we determine the maximum Type I error rate and discuss the worst case rules leading to the maximum Type I error rate. We provide a short discussion in Section 5 and in Section 6, we provide recommendations that can be implemented in platform trials to mitigate the risk for trial integrity.

## 2 Methods

### 2.1 General Notation and Log‐Rank Test

We assume an event‐driven platform trial with J treatment arms and one common control group. Let $X_{i}^{j}$ denote the survival outcome of patient i, $i\in \left\{1,\ldots ,n_{j}\right\}$, with treatment j,j∈{0,…,J} and a total sample size of $n_{j}$ in each treatment arm j. Treatment j=0 denotes the common control group. We assume exponentially distributed survival times, that is,

(1) $$X_{i}^{j}∼\mathrm{Exp}\left(\lambda_{j}\right),\;j\in \left\{0,\ldots ,J\right\}$$

and proportional and constant hazards such that the hazard ratio is denoted by $\theta_{j}=\lambda_{j}/\lambda_{0}$, where $\lambda_{j}$ and $\lambda_{0}$ are the hazard rates in treatment arm j and the control arm, respectively. The median survival time $m_{j}$ in each arm is defined as $m_{j}≔\ln\left(2\right)/\lambda_{j}$ which results from assuming an exponential distribution for the survival times, and the hazard rate can be correspondingly expressed as

(2) $$\lambda_{j}=\frac{\ln\left(2\right)}{m_{j}}.$$

Assuming constant and proportional hazards, a log‐rank test at level α can be performed to test the null hypothesis $H_{0}:\lambda_{j}=\lambda_{0}$. The corresponding log‐rank test statistic $Z_{j}$ depends on the number of events $d_{j}$ in treatment j and the number of events in the control arm $d_{0}$. Under the null hypothesis $Z_{j}$ is asymptotically standard normally distributed, that is, $Z_{j}\overset{a}{∼}N\left(0,1\right)$, or equivalently $Z_{j}^{2}\overset{a}{∼}\chi_{1}^{2}$. As the $\chi^{2}$ distribution, which is commonly used for the log‐rank test, is agnostic of the direction of differences, it is always a two‐sided test. In order to test for superiority on a two‐sided significance level α, we hence can reject the null hypothesis if $Z_{j}^{2}>\chi_{1,1-\alpha }^{2}$ and the estimated hazard ratio ${\hat{\theta }}_{j}$ shows in the right direction.

In general, $Z_{j}$ is normally distributed with

(3) $$\begin{array}{ll} E\left(Z_{j}\right) & =\sqrt{d_{j\cup 0}}\frac{\sqrt{\kappa }}{1+\kappa }\log\left({\hat{\theta }}_{j}\right),\\ \mathrm{Var}\left(Z_{j}\right) & =1, \end{array}$$

where $d_{j\cup 0}≔d_{j}+d_{0}$ denotes the pooled number of events in treatment arm j and the control group, and κ denotes the randomisation ratio $n_{j}/n_{0}$.

While survival analyses could be time‐based (e.g., analyses after a specific follow up time) the power is primarily driven by the number of observed events. Hence, the primary analysis of the survival outcomes is often event‐driven. The number of events ($d_{j\cup 0}$) required to show a specific effect $\theta_{j}$ depends on the significance level α, the power 1−β, and randomisation ratio κ [29, 30]. The total sample size $n_{j}+n_{0}$ can then determined by $d_{j\cup 0}/\Phi$, where Φ denotes the pooled probability of an event. One possibility to estimate this probability is to calculate Φ as the average event probability of the treatment arm j and the control arm, that is, $\Phi =\frac{1}{2}\left(\phi_{j}+\phi_{0}\right)$, where the probability of an event at a time point s

(4) $$\phi_{j}=g\left(\lambda_{j},s\right)≔\left(\begin{array}{ll} \frac{s}{a}-\frac{1-\exp\left(-\lambda_{j}\cdot s\right)}{\lambda_{j}\cdot a}, & \text{if}\;s\leq a\\ 1-\exp\left(-\lambda_{j}\cdot s\right)\cdot \frac{\exp\left(\lambda_{j}\cdot a\right)-1}{\lambda_{j}\cdot a}, & \text{if}\;s>a \end{array}\right.,$$

is a function depended on the assumed hazard rate $\lambda_{j}$, the accrual time a and the follow‐up time f. The latter is included when s>a, then s≔a+f. For further details refer to, for example, Kim and Tsiatis [31] and Wassmer [30].

### 2.2 Specific Considerations on Platform Trial Design

**FIGURE 1:**
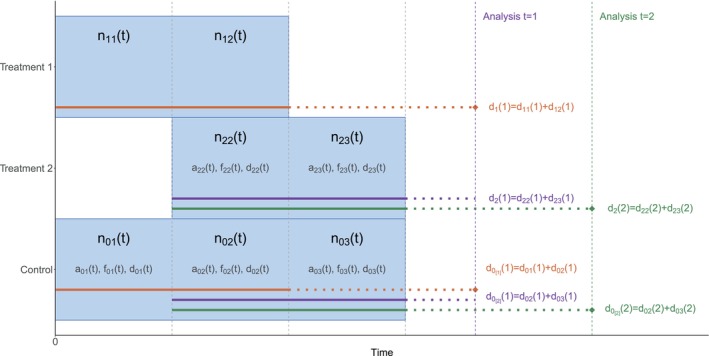
Schematic overview of the notation for a platform trial with two treatments and one control group. Boxes for each treatment denote the recruitment time leading to three recruitment periods. Solid lines denote the accrual periods for each treatment and its concurrent control and dotted lines denote the follow‐up times. Analysis of Treatment 1 and Treatment 2 are performed when the required number of events are observed leading to two analyses. $n_{\mathrm{jr}}\left(t\right)$ denote the number of patients in arm j in recruiting period with accrual time $a_{\mathrm{jr}}\left(t\right)$ and follow‐up time $f_{\mathrm{jr}}\left(t\right)$. Corresponding events are denoted as $d_{j}\left(t\right)$. The (potential) time dependency of the quantities is indicated by defining them as functions of time t.

In the following we will discuss and describe some further specifics of platform trials with survival time endpoints. An example of such a trial design is given in Figure 1 which is discussed further below.

#### 2.2.1 Recruitment Period

In a platform study, arms can be opened and closed over time. Allowing treatments to enter the platform at a later stage or to stop recruitment to an arm before recruitment is stopped for the whole trial naturally leads to recruitment periods r,r∈{1,…,R}. These periods are determined by all times points where the recruitment to a new arm starts and time points where recruitment to an arm ends. We can therefore split the overall sample sizes $n_{j},\;j\in \left\{0,\ldots ,J\right\},$ of the treatment arms and control arm by recruitment periods such that $n_{j}=\sum_{r}n_{\mathrm{jr}}$, where $n_{\mathrm{jr}}$ denotes the sample size for arm j in period r, and $n_{\mathrm{jr}}=0$ if no subjects are recruited in arm j at period r. All other parameters can be split in a similar manner. For example, the overall number of events $d_{j}$ for treatment j can be described as the sum of events $d_{\mathrm{jr}}$ observed from the $n_{\mathrm{jr}}$ patients recruited in recruitment period r.

#### 2.2.2 Concurrent Controls

For the comparison of treatment j to the control arm, often only control patients concurrently enrolled to treatment j are used. We will use this approach for the remainder of the paper. Where necessary, parameters for the concurrent control group are denoted with an additional subscript [j] (e.g., $d_{0_{\left[j\right]}}$ represents the number of events from the $n_{0_{\left[j\right]}}$ control patients concurrently enrolled to treatment arm j).

#### 2.2.3 Dependency on Analysis Time

The parameters and quantities in survival analyses depend on the time of analysis t,t∈{1,…,T}. When a treatment arm j together with the concurrent control arm reaches the required number of events for a (prespecified) analysis, we can define the information observed up to analysis t as a function of t. For example, the number of events in treatment arm j observed at analysis t is then denoted as $d_{j}\left(t\right)$. Similarly, the sample size, the follow up time and potentially the accrual time can depend on analysis t. In a multi‐arm platform trial we can additionally specify the corresponding quantities at analysis t for other arms, for example, the sample size $n_{j^{*}}\left(t\right)$ or the number of events $d_{j^{*}}\left(t\right)$, which accrued in another arm $j^{*}$ up to analysis t or the accrual time $a_{j^{*}}\left(t\right)$ and follow up time $f_{j^{*}}\left(t\right)$. Some quantities such as the number of events per arm should not be available to the experimenter at the time of analysing arm j. However, as discussed below, they might be fully or partially leaked and hence gain a special relevance.

#### 2.2.4 Illustrative Example

For illustration purposes and simplicity, we consider a concrete example of a platform trial with two treatments and one shared control for the remainder of the manuscript. Figure 1 depicts such a platform trial where one treatment joins the platform later resulting in three recruitment periods r∈{1,2,3} and two analyses t∈{1,2}. In this trial, for example, the (time‐dependent) total number of patients $n_{2}\left(t\right)$ recruited up to analysis t=1 for Treatment 2 can be decomposed into $n_{2}\left(t\right)=n_{22}\left(t\right)+n_{23}\left(t\right)$, that is, the sum of patients recruited in each recruitment period. Since Treatment 2 joins the platform later no patients were recruited to arm 2 during period 1 ($n_{21}\left(t\right)=0$). The corresponding (time‐dependent) accrual $a_{2}\left(t\right)$ and follow‐up times $f_{2}\left(t\right)$ of these patients can be split per recruitment period as well (e.g., $a_{22}\left(t\right)$ and $f_{22}\left(t\right)$ denote these quantities for patients recruited in period r=2, and $a_{23}\left(t\right)$ and $f_{23}\left(t\right)$ for patients recruited in period r=3). Note that in this example (Figure 1) the accrual periods for arm 2 are completed by the time arm 1 is analysed (t=1), and hence the sample sizes and accrual periods are fixed constants after that time. Follow up times, however, still depend on the analysis. In general, an analysis of an arm could occur while patients are still recruited to another arm and hence also accrual could become time dependent. The number of observed events $d_{2}\left(1\right)$ in treatment arm 2 until analysis t=1, which is the time point of primary analysis for Treatment 1, is the sum of events $d_{22}\left(1\right)$ and $d_{23}\left(1\right)$ from patients recruited in recruitment periods r=2 and r=3. Similarly, events for Treatment 2 observed until analysis t=2, which is the time point of primary analysis for Treatment 2, are denoted by $d_{2}\left(2\right)=d_{22}\left(2\right)+d_{23}\left(2\right)$. With regard to the control group, the additional subscript indicates events that are observed from the $n_{0_{\left[2\right]}}\left(t\right)=n_{02}\left(t\right)+n_{03}\left(t\right)$ control patients which were enrolled concurrently to Treatment 2. Hence, $d_{0_{\left[2\right]}}\left(1\right)$ and $d_{0_{\left[2\right]}}\left(2\right)$ correspond to events observed until analysis t=1 and t=2 from the control patients concurrently enrolled to Treatment 2. Of note, the pooled number of events in the log‐rank test statistic would then be defined based on concurrent events until analysis t only, that is, $d_{j\cup 0}$ would be determined by $d_{j}\left(t\right)+d_{0_{\left[j\right]}}\left(t\right)$ in Equation (3) at analysis t.

### 2.3 Information Leakage in Platform Trials

In the following we assume, without loss of generality, that we only have two treatment arms as depicted in Figure 1 and that Treatment 1 reaches the required total number of events $d_{1\cup 0}$ at analysis t=1 *before* Treatment 2 reaches its required number of events $d_{2\cup 0}$. The experimenter could use information released at analysis t=1, for example, comparative efficacy results from Treatment 1 or operational information from monitoring to adapt the trial such that an ad‐hoc efficacy analysis for Treatment 2 is conducted, the sample size for treatment arm 2 is recalculated or other adaptations could be implemented.

We focus on the first possibility and assume that if the released information indicates potentially favourable results, an ad‐hoc efficacy analysis (without any adjustment of the significance level) will be performed for Treatment 2. The ad‐hoc efficacy analysis will then serve as an immediate primary analysis even though the initial number of required events has not been reached yet. Otherwise, the experimenter waits until the planned required number of events is observed to perform the primary analysis. Given that the decision to perform an ad‐hoc analysis is data‐driven and dependent on the analysis for Treatment 1 at analysis t=1, the Type I error rate may be inflated.

Since the trial is event‐driven (i.e., the primary analysis will be performed once the required number of pooled events in treatment arm 2 and the control is reached), the pooled number of events $d_{2\cup 0}\left(t\right)$ for Treatment 2 and the control is constantly monitored to trigger the primary analysis and is thus assumed to be known at all times. This is one potential source of data leakage, which can be used to alter the trial design. However, further data such as comparative efficacy results from Treatment 1 against its concurrent control group (e.g., the estimated hazard ratio ${\hat{\theta }}_{1}\left(1\right)={\hat{\lambda }}_{1}\left(1\right)/{\hat{\lambda }}_{0_{\left[1\right]}}\left(1\right)$ at analysis t=1) or even information on the treatment and concurrent control arms directly (e.g., the estimated median survival time in the arms, ${\hat{m}}_{1}\left(1\right)$ and ${\hat{m}}_{0_{\left[1\right]}}\left(1\right)$) can become available at the time of analysis t=1 as well. To distinguish true values from data based estimates, we use a hat for the latter; for example, ${\hat{m}}_{j}$ denotes the estimated median in arm j and ${\hat{\theta }}_{j}$ the estimated hazard ratio. Using the leaked information on the shared control group, estimates of comparative efficacy results for Treatment 2 and its concurrent control can be obtained. These estimates based on leaked data will be denoted with a tilde, for example, ${\tilde{\theta }}_{j}$ denotes the estimate of the hazard ratio for arm j using leaked data. Note that the estimation process based on leaked data requires further assumptions (e.g., that survival times are exponentially distributed, the recruitment to the trial is equal and constant over time or that the hazard rates are constant over time and proportional) and the derived estimates usually differ from the data‐based estimates, that is, ${\tilde{\theta }}_{j}\left(1\right)\neq {\hat{\theta }}_{j}\left(1\right)$.

Let $D_{L}\left(1\right),L\in \left\{L1,L2,L3,L4\right\}$ denote the leaked data at analysis t=1 which can be used to adapt the trial (i.e., decide to stop for an ad‐hoc analysis of Treatment 2 at analysis t=1 or to wait until the planned analysis t=2), where L denotes the leaked information. We consider four different scenarios of leaked data L1 to L4, which are described in detail in Table 1. In short,

- in L1 the actual number of events in arm 2, $d_{2}\left(1\right)$, and the number of events in the concurrent control arm, $d_{0_{\left[2\right]}}\left(1\right)$, are leaked,
- in L2 the pooled number of events for arm 2 and concurrent controls, $d_{2\cup 0}\left(1\right)$, and the current observed hazard ratio between arm 2 and concurrent controls, ${\hat{\theta }}_{2}\left(1\right)$, are leaked,
- in L3 an estimate of the number events for arm 2, ${\tilde{d}}_{2}\left(1\right)$, and concurrent controls, ${\tilde{d}}_{0_{\left[2\right]}}\left(1\right)$, is derived based on leaked information on the median survival times in the control group ${\hat{m}}_{0_{\left[1\right]}}$ together with information on recruitment times and the pooled number of events for arm 2 and concurrent controls, $d_{2\cup 0}\left(1\right)$,
- and in L4 the pooled number of events, $d_{2\cup 0}\left(1\right)$, is leaked and an estimate of the hazard ratio ${\tilde{\theta }}_{2}\left(1\right)$ is derived based on the median survival times in the control group ${\hat{m}}_{0_{\left[1\right]}}$ together with information on recruitment times.

**TABLE 1:** Description of different scenarios of information release (leaked data) and a detailed explanation of how the released parameters are used directly or after multiple estimation steps for trial adaptation. The hat operator defines estimates from the data, the tilde defines estimates from leaked data. The data finally used to adapt the trial and to derive the test statistic for arm 2 (Equation (5)) is denoted as $D_{L}\left(1\right)$.

Leaked information	Details on usage ofleaked information	Interim data $D_{L}\left(1\right)$ used for adaptation
**Leakage Treatment 2:** $d_{2}\left(1\right)$: events in arm 2 $d_{0_{\left[2\right]}}\left(1\right)$: events in control	**L1**. Assuming the experimenter knows the number of events per arm $d_{2}\left(1\right)$ and $d_{0_{\left[2\right]}}\left(1\right)$ at analysis t=1.	$D_{L1}\left(1\right)=\left(d_{2}\left(1\right),d_{0_{\left[2\right]}}\left(1\right)\right)$
**Monitoring Treatment 2:** $d_{2\cup 0}\left(1\right)$: pooled events in arm 2 and control **Leakage Treatment 2:** ${\hat{\theta }}_{2}\left(1\right)$: observed hazard ratio	**L2**. In addition to the number of events, we assume that also the current observed hazard ratio ${\hat{\theta }}_{2}\left(1\right)$ is available.	$D_{L2}\left(1\right)=\left(d_{2\cup 0}\left(1\right),{\hat{\theta }}_{2}\left(1\right)\right)$
**Monitoring Treatment 2:** $d_{2\cup 0}\left(1\right)$: pooled events in arm 2 and control **Recruitment Information:** $n_{02}\left(1\right)$, $n_{03}\left(1\right)$: sample size in control $a_{02}\left(1\right)$, $f_{02}\left(1\right)$, $a_{03}\left(1\right)$, $f_{03}\left(1\right)$: accrual and follow time in control **Analysis Treatment 1:** ${\hat{m}}_{0_{\left[1\right]}}\left(1\right)$: median in control	**L3**. Efficacy results comparing treatment 1 to the control is used as follows: The median survival for the control group ${\hat{m}}_{0_{\left[1\right]}}\left(1\right)$ is released. Assuming exponential survival the hazard rate ${\tilde{\lambda }}_{0_{\left[1\right]}}\left(1\right)$ can be estimated based on the median using Equation (2). The probability of an event in the control patients ${\tilde{\phi }}_{02}=g\left({\tilde{\lambda }}_{0_{\left[1\right]}}\left(1\right),s\left(1\right)\right)$ and ${\tilde{\phi }}_{03}=g\left({\tilde{\lambda }}_{0_{\left[1\right]}}\left(1\right),s\left(1\right)\right)$ can be estimated using Equation (4) given the hazard rate, the accrual and follow‐up time for the control patients $n_{02}$ and $n_{03}$ at analysis t=1 which takes place at time s(1). The number of events in the control group can then be estimated from the information above ${\tilde{d}}_{0_{\left[2\right]}}\left(1\right)={\tilde{d}}_{02}\left(1\right)+{\tilde{d}}_{03}\left(1\right)$. Given the total number of events, the number of events in the treatment arm can be estimated as ${\tilde{d}}_{2}\left(1\right)=d_{2\cup 0}\left(1\right)-{\tilde{d}}_{0_{\left[2\right]}}\left(1\right)$.	$D_{L3}\left(1\right)=\left({\tilde{d}}_{2}\left(1\right),{\tilde{d}}_{0_{\left[2\right]}}\left(1\right)\right)$
**Monitoring Treatment 2:** $d_{2\cup 0}\left(1\right)$: pooled events in arm 2 and control **Recruitment Information:** $n_{02}\left(1\right)$, $n_{03}\left(1\right)$: sample size in control $a_{02}\left(1\right)$, $f_{02}\left(1\right)$, $a_{03}\left(1\right)$, $f_{03}\left(1\right)$: accrual and follow time in control $n_{2}\left(1\right)$: sample size in Treatment 2 $a_{2}\left(1\right)$, $f_{2}\left(1\right)$: accrual and follow time in Treatment 2 **Analysis Treatment 1:** ${\hat{m}}_{0_{\left[1\right]}}\left(1\right)$: median in control	**L4**. Extending L3, we continue the estimation: Given the number of patients $n_{2}\left(1\right)$ in Treatment 2 and the estimated number of events ${\tilde{d}}_{2}\left(1\right)$, we can estimate the event probability ${\tilde{\phi }}_{2}$ for Treatment 2 and use Equation (4) to solve for a estimated hazard rate ${\tilde{\lambda }}_{2}\left(1\right)$. Assuming a constant hazard rate for the control group and in general proportional hazards, the hazard ratio can be estimated as ${\tilde{\theta }}_{2}\left(1\right)={\tilde{\lambda }}_{2}\left(1\right)/{\tilde{\lambda }}_{0_{\left[1\right]}\left(1\right)}$.	$D_{L4}\left(1\right)=\left(d_{2\cup 0}\left(1\right),{\tilde{\theta }}_{2}\left(1\right)\right)$

**FIGURE 2:**
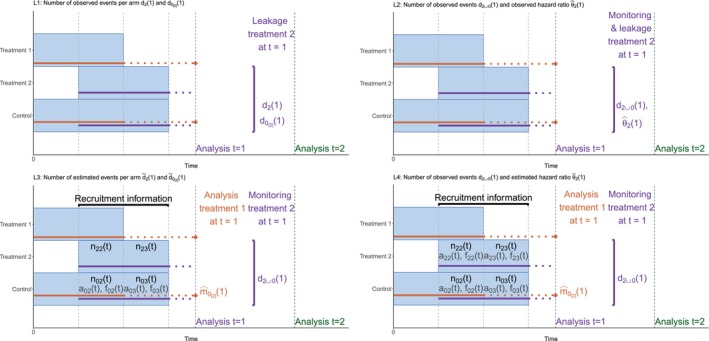
Schematic overview of the notation and different types of information leakage. Boxes for each treatment denote the recruitment time. For each scenario only the information that is leaked is displayed on the graph. $n_{\mathrm{jr}}\left(t\right)$ denote the number of patients in arm j in recruiting period with accrual time $a_{\mathrm{jr}}\left(t\right)$ and follow‐up time $f_{\mathrm{jr}}\left(t\right)$. The corresponding number of events is denoted as $d_{j}\left(t\right)$. Details on the scenarios are given in Table 1.

In L1 and L2 leaked information from the actual observed data for Treatment 2 and the control arm are used. While L1 contains the individual number of events in treatment arm 2 and the control arm, respectively, in L2 only the pooled number of events in these arms is known. However, in L2 additionally the observed hazard ratio is known. In L3 and L4, the pooled number of events in Treatment 2 and the control is known. The median survival in the common control arm, published with the analyses of Treatment 1, is used to estimate comparative results for treatment arm 2. Thus, L1 and L2 represent leakage scenarios where actual information from the treatment arm 2 is known, whereas L3 and L4 represent scenarios where leaked information from a platform trial was used to estimate comparative results for Treatment 2 and the control. In each scenario, different degrees of knowledge from the data are assumed (see Table 1). Additionally, an illustration of the different options is depicted in Figure 2. In practise, L1 and L2 can happen in open‐label trials or in blinded trials during (planned) trial adaptations such as a sample size reassessment where such data is monitored and used for adaptation but insufficient firewalls were implemented resulting in leakage of data. Furthermore, unblinding can result from monitoring of pooled events in platform trials with multiple treatment arms: the number of events per arm may be deducted from continuously observing the pooled number of events of each arm with its concurrent controls. Especially, when all arms start recruitment at the same time, a new event which increases, for example, $d_{1\cup 0}\left(t\right)$ but did not increase $d_{2\cup 0}\left(t\right)$ must be an event in treatment arm 1. On the other hand, an event which increases both $d_{1\cup 0}\left(t\right)$ and $d_{2\cup 0}\left(t\right)$ will be an event in the control group. For delayed entries of treatment arms to the trials, this becomes slightly more complicated but is still possible to some extend. Thus, L1 and L2 represent cases of internal unblinding, with L2 being a full unblinded case. In addition to the pooled number of events of each arm with its concurrent control, platform trials offer further information on the control group, in particular when results from analyses of treatment arms against the control group are published. L3 and L4 represent two potential usage of such information, in which the regularly monitored pooled number of events can be complemented by efficacy results from another arm.

Based on the leaked information, we now can estimate a log‐rank test statistic ${\tilde{Z}}_{2,L}\left(1\right)$ for Treatment 2 and the control at analysis t=1, where L∈{L1,L2,L3,L4}, by plugging the available information and further derived estimates into Equation (3). In the situation of our illustrative example (see Figure 1) this leads to the following test statistics

(5) $$\begin{array}{cc} {\tilde{Z}}_{2,L1}\left(1\right) & =\sqrt{d_{2\cup 0}\left(1\right)}\cdot \frac{1}{2}\cdot \log\left(\frac{d_{2}\left(1\right)}{d_{0_{\left[2\right]}}\left(1\right)}\right)\\ {\tilde{Z}}_{2,L2}\left(1\right) & =\sqrt{d_{2\cup 0}\left(1\right)}\cdot \frac{1}{2}\cdot \log\left({\hat{\theta }}_{2}\left(1\right)\right)\\ {\tilde{Z}}_{2,L3}\left(1\right) & =\sqrt{d_{2\cup 0}\left(1\right)}\cdot \frac{1}{2}\cdot \log\left(\frac{{\tilde{d}}_{2}\left(1\right)}{{\tilde{d}}_{0_{\left[2\right]}}\left(1\right)}\right)\\ {\tilde{Z}}_{2,L4}\left(1\right) & =\sqrt{d_{2\cup 0}\left(1\right)}\cdot \frac{1}{2}\cdot \log\left({\tilde{\theta }}_{2}\left(1\right)\right), \end{array}$$

where the ratio of observed events or estimated events is used as a surrogate for the hazard ratio in L1 and L3, respectively. For simplicity, we assumed that the randomisation ratio is equal for all arms (i.e., κ=1). The aim in the following is to quantify the extent of Type I error inflation when (full) knowledge of the data is available or when information from the common control is released.

### 2.4 Maximum Type I Error Rate

To assess the impact if leaked data is used for modifications of the remaining treatment arms in the trial we consider the maximum Type I error rate (see e.g., [22, 23, 24]). We assume that the experimenter of Treatment 2 decides at analysis t=1 to perform an ad‐hoc efficacy analysis for arm 2 or to wait until the primary analysis for arm 2 based on the leaked data such that the conditional Type I error rate is maximised. This leads to a worst case scenario and will also maximise the unconditional Type I error rate for the comparison of Treatment 2 and the control. Throughout the document we will consider the marginal Type I error rate for the comparison of Treatment 2 and the control only.

The conditional Type I error rate at analysis t given the leaked data of analysis t=1, that is, $D_{L}\left(1\right)$, is

(6) $${\mathrm{CE}}_{L}\left(t\right)=P_{H_{0}}\left(\left.Z_{2}^{2}\left(t\right)>\chi_{1,1-\alpha }^{2}\;\cap \;I\left({\hat{\theta }}_{2}<1\right)\right|D_{L}\left(1\right)\right),$$

where L∈{L1,L2,L3,L4}, $P_{H_{0}}$ is the probability under the null hypothesis, and I(⋅) is an indicator function which is 1 if its argument is true and 0 otherwise as for a one‐sided test at significance level α the null hypothesis is only rejected if in addition the hazard ratio shows in the right direction (${\hat{\theta }}_{2}<1$).

${\mathrm{CE}}_{L}\left(1\right)$ denotes the conditional error rate of the log‐rank test for arm 2 versus concurrent controls at analysis t=1, and ${\mathrm{CE}}_{L}\left(2\right)$ denotes the conditional error rate of the log‐rank test for arm 2 at analysis t=2. Following a similar approach as described in Wassmer and Brannath [32], Graf and Bauer [22], Graf et al. [23] and Żebrowska, Posch, and Magirr [24], the maximum Type I error rate when comparing Treatment 2 to the control can be determined by

(7) $$E_{L}=\int \max_{t\in \left(1,2\right)}\left({\mathrm{CE}}_{L}\left(t\right)\right)\cdot f\left(D_{L}\left(1\right)\right)\;{\mathrm{dD}}_{L}\left(1\right),$$

where $f\left(D_{L}\left(1\right)\right)$ denotes the probability density function of the data $D_{L}\left(1\right)$ used for adaptation. The Type I error rate is thus maximised when choosing to stop at analysis t given each outcome that maximises the equations above. The maximum Type I error rate can serve as an upper bound on potential inflation of the Type I error rate inflation.

While for L1 and L2 the observed information up to analysis t=1 can be used, for L3 and L4 estimations of the efficacy data are obtained based on leaked data, which makes the determination of the null distribution difficult. To analytically calculate the maximum Type I error rate, it is necessary to know the null distribution of the log‐rank test statistic resulting from each leakage scenario. Therefore, we used simulations to obtain approximate maximum Type I error rates for each of the information releases and varying time points of when Treatment 2 joins the platform. For L2 the estimated log‐rank test statistic can be analytically estimated which we used in Section 3.2 in addition to the simulations. In addition, the above equation provides the maximum Type I error rate but does not necessarily provide the worst case rule when to stop at analysis t. Therefore, we evaluated in additional simulations whether a worst case rule as a function of the information releases up to analysis t=1 can be obtained.

## 3 Simulation Study

We simulated the 3‐arm trial design described above with $N_{\mathrm{sim}}=10^{6}$ runs for each simulation scenario to estimate the maximum Type I error rate provided the different information leakages. Under the global null hypothesis we assumed exponential survival for all arms with a median survival of 5 months. Simulation results varying the treatment effect of Treatment 1 (moderate and high effect) are provided in the Supporting Information Section A.1, and simulation results varying the median survival under the global null in the Supporting Information Section A.2. Additionally, simulations using different data generating models (multi‐state model and delayed treatment effects) are provided in the Supporting Information Section A.3. For each arm we assumed an accrual duration of 10 months to recruit 100 patients per arm with an equal allocation ratio at all times. We assume that Treatment 1 and the control group start recruitment at the same time and varied the entry of Treatment 2 to the platform at time 0, 3, 5 and 7 months, corresponding to $n_{01}=0,30,50$ and 70 control patients already in the trial when Treatment 2 joins the platform. An entry at time 0 corresponds to a fixed multi‐arm trial, where all control patients are shared among both treatment arms (for simplicity in our notation this means $n_{11}=n_{01}=0$). A late entry of Treatment 2 means a longer recruitment of the control, a higher sample size in the control (i.e., additional $n_{03}$ patients), less shared controls with Treatment 1 and thus the presence of non‐concurrent controls (e.g., $n_{01}$ non‐concurrent controls for Treatment 2). For each analysis, a log‐rank test (1‐sided) comparing a treatment to only concurrent controls at an alpha level of 2.5% is performed. A total of 161 events (corresponding to an assumed hazard ratio of 0.6) is needed for a treatment and control comparison to trigger the primary analysis. We considered analysis time points of Treatment 1 at 20%,…,100% of required events for Treatment 1 and the control, where 100% corresponds to 161 events, that is, the primary analysis. We implemented administrative censoring, that is, event times after the required number of events were censored at the time of analysis. No additional censoring was simulated. At each analysis for Treatment 1, the four different information leakages L1 to L4 are published which can be used to decide for an ad‐hoc efficacy stop of Treatment 2. In summary, for each information leakage, we evaluate the impact of the following aspects on the Type I error rate for Treatment 2:

- the time point of Treatment 2 joining the platform, and
- the time point of Treatment 1 releasing information,

while keeping all other factors fix. A table summarising the main input parameters for the simulations is provided in the Supporting Information Section D. The input parameters to generate the data are the same for each leakage scenario. However, output parameters from the simulation differ for each leakage scenario. For example, for L1, only the individual number of events per arm are used, while for L2 the pooled number of events and the hazard ratio is used. Details on the different usage of information for each leakage scenario is provided in Table 1.

### 3.1 Estimation of the Maximum Type I Error Rate

To estimate the maximum Type I error rate as defined in Equation (7) based on simulated data, one needs to compute the conditional error rate ${\mathrm{CE}}_{L}\left(t\right)$ if one stops the trial at time t=1 and at time t=2, conditional on the observed (leaked) data at time t=1, that is, $D_{L}\left(1\right)$. To estimate these error rates based on simulations, we need to condition on all realisations of the simulation where a specific outcome was observed and compute the error rate for these simulations if one would conduct the analysis on the spot or at the planned primary analysis. The estimate of both conditional error rates is possible in the simulations as we have all the data available for all arms until the respective (primary) analysis but can assume that we would have stopped the trial and analyse arm 2 together with the (primary) analysis of arm 1. The details of the estimation process can be found in Algorithm 1, where the leaked information $D_{L}\left(1\right)$ is, without loss of generality, considered to be of discrete nature.

In scenarios L1 and L3 the leaked data consists of the number of events and is naturally of discrete nature. In scenarios L2 and L4 key information that is leaked is of continuous nature (hazard ratio ${\hat{\theta }}_{2}\left(1\right)$ for L2 and ${\tilde{\theta }}_{2}\left(1\right)$ for L4), as can be seen in Table 1 and Figure 2. In these cases, one needs to categorise the leaked data and estimate the conditional errors for these categorised versions of the leaked data. In our case, we categorised the hazard ratios into intervals based on the quantiles of the leaked hazard ratios and base all further computations for the maximum Type I error rate on this binned version of the leaked data. The derived maximum type on error rate estimate is an approximation in these cases.

The decision rule (10) when to stop the trial in order to maximize the conditional error rate given the leaked data L is called ‘approximate adaptation rule’ throughout this document to reflect the potential categorisation of the data.

ALGORITHM 1 **Estimation of maximum Type I error.**

1. Definitions:
  - Let $s,s\in \left\{1,\ldots ,N_{\mathrm{sim}}\right\}$ denote the number of the simulation run.
  - Let $D_{L,s}\left(1\right)$ be a vector which denotes the leaked information in simulation s observed at analysis t=1, or a categorised version thereof. *For example*, $D_{L,s}\left(1\right)$ *could be the number of events in arm 2 and the concurrent control arm in scenario* L1, that is, $D_{L1,s}\left(1\right)={\left(d_{2,s}\left(1\right),d_{0_{\left[2\right]},s}\left(1\right)\right)}^{⊤}$.
  - Let $S_{L,s^{*}}≔\left\{s\;|\;D_{L,s}\left(1\right)=D_{L,s^{*}}\left(1\right)\right\}$ define an index set of simulations where the leaked data $D_{L,s^{*}}\left(1\right)$ was observed. *To continue the above example*, $S_{L,s^{*}}$ *could denote the index set of all simulations, where* $D_{L1,s}\left(1\right)$ *equals the leaked information in simulation* $s^{*}$, for example, $s^{*}=1$. *This could be for example the vector* $D_{L1,s^{*}}\left(1\right)=D_{L1,1}\left(1\right)={\left(20,30\right)}^{⊤}$ *which would mean that in that simulation 20 events were observed in arm 2 and 30 events in the concurrent control arm, and* $S_{L,1}$ *denotes the index set of simulations where this combination of events was observed*.
2. For each *unique* combination of the leaked data $D_{L,s^{*}}\left(1\right)$ derive the corresponding index set $S_{L,s^{*}}$, and compute the corresponding conditional errors and maximum Type I error rate given as follows:
  - Obtain the estimated conditional error rate at analysis t=1 as the relative frequency of simulations with a rejection at analysis t=1 for the leaked information $D_{L,s^{*}}\left(1\right)$:
(8) $$R_{S_{L,s^{*}}}\left(1\right)≔\frac{1}{|S_{L,s^{*}}|}\sum_{s\in S_{L,s^{*}}}I\left({\hat{Z}}_{2,s}^{2}\left(1\right)>\chi_{1,0.95}^{2}\;\cap \;I\left({\hat{\theta }}_{2,s}\left(1\right)<1\right)\right),$$
    where $|S_{L,s^{*}}|$ is the cardinality of the index set, that is, the number of simulations which led to the same leaked data $D_{L,s^{*}}\left(1\right)$, ${\hat{Z}}_{2,s}^{2}\left(1\right)$ is the estimated log‐rank test statistic for arm 2 in simulation s at time t=1, and ${\hat{\theta }}_{2,s}\left(1\right)$ the corresponding estimated hazard ratio.
  - Obtain the estimated conditional error rate at analysis t=2 as the relative frequency of simulations with a rejection at analysis t=2 for the leaked information $D_{L,s^{*}}\left(1\right)$:
(9) $$R_{S_{L,s^{*}}}\left(2\right)≔\frac{1}{|S_{L,s^{*}}|}\sum_{s\in S_{L,s^{*}}}I\left({\hat{Z}}_{2,s}^{2}\left(2\right)>\chi_{1,0.95}^{2}\;\cap \;I\left({\hat{\theta }}_{2,s}\left(2\right)<1\right)\right),$$
    with estimates for the log‐rank statistic and hazard ratio based on the full data at analysis t=2.
  - The Type I error rate is maximised by if, based on the leaked data, one stops at analysis t=1 if this leads to the larger estimated conditional error rate compared to continuing to analysis t=2, that is,

(10) $$R_{S_{L,s^{*}}}\left(1\right)>R_{S_{L,s^{*}}}\left(2\right).$$

The resulting conditional error rate when using this adaptation rule can be estimated by

(11) $$\max\left(R_{S_{L,s^{*}}}\left(1\right),R_{S_{L,s^{*}}}\left(2\right)\right).$$

3 The overall maximum Type I error rate is estimated by averaging over the maximum of the estimated conditional error rates (11) using a weighted mean

(12) $${\hat{E}}_{L}=\sum_{\text{unique}\;\mathrm{set}\;s^{*}}\frac{|S_{L,s^{*}}|}{N_{\mathrm{sim}}}\cdot \max\left(R_{S_{L,s^{*}}}\left(1\right),R_{S_{L,s^{*}}}\left(2\right)\right),$$

over all unique sets of leaked data $s^{*}$.

### 3.2 Evaluation of Bias for the Estimated Maximum Type I Error

Notably, the estimation of the maximum Type I error rate (Equation (12)) following the approximate adaptation rule (Equation (10)) may lead to bias of the actual maximum error rate: On the one hand due to the categorisation of the hazard ratios there will be a negative bias as we average in our rule. On the other hand the use of the maximum of two estimates might lead to a positive bias, that is, overestimating the maximum Type I error rate. Furthermore, the estimations of the conditional error rates are dependent on the occurrences of leaked data in the simulation which adds another layer of noise if realisations of leaked data are only present a few times. Therefore, we implemented additional simulations to evaluate the impact of this two sources of bias and to obtain a lower bound for the maximum Type I error rate.

Thus, for L1, L3 and L4 we approximated the adaptation rule based on the estimated conditional error rates obtained by Equation (10), including the categorisation of the estimated hazard ratio using a nearest neighbour smoothing approach to reduce to potential noise followed by a linear discriminant analysis (LDA) to separate cases of $R_{S_{L,s^{*}}}\left(1\right)>R_{S_{L,s^{*}}}\left(2\right)$ from $R_{S_{L,s^{*}}}\left(1\right)<R_{S_{L,s^{*}}}\left(2\right)$. Cases of equal rates are assigned to the latter class of $R_{S_{L,s^{*}}}\left(1\right)<R_{S_{L,s^{*}}}\left(2\right)$ when defining the new adaptation rule. We ran an independent simulation using the new adaptation rule using the LDA to derive an estimate of the Type I error rate resulting from using this rule. As this rule only approximates the approximated adaptation rule Equation (10), the resulting Type I error rate gives the lower bound for the maximum Type I error rate. For a detailed description of the nearest neighbour approach and LDA algorithm see Supporting Information Section B. For L2, the worst case rule is obviously to stop at analysis t=1 if the already observed log‐rank test statistic is already significant. Therefore, we ran an independent simulation using this stopping rule to estimate the maximum Type I error rate and compared it to the approximate maximum Type I error rate (Equation (12)). Throughout the document we call the LDA based rule for L1, L3 and L4 and also the worst case rule based on the log‐rank statistic (Equation (3)) for L2 the ‘simplified adaptation rules’.

The combinations of the information used for the adaptation rules (see Table 1) and the strategy to implement both rules is summarised in Table 2.

**TABLE 2:** Overview on combination of information used (columns L1 to L4, see Table 1) and the two adaptation rules used in the simulations. For detailed descriptions of the simplified adaptation rule we refer to the supplement. The maximum of estimated CEs algorithm is defined in Section 3.1 above.

	Information used			
	L1	L2	L3	L4
Approximate adaptation rule (Equation (10))	Maximum of estimated CEs	Maximum of estimated CEs based on categories	Maximum of CEs	Maximum of estimated CEs based on categories
Simplified adaptation rule	LDA	Based on log‐rank statistic	LDA	LDA

## 4 Results

### 4.1 Estimation of the Maximum Type I Error Rate

**FIGURE 3:**
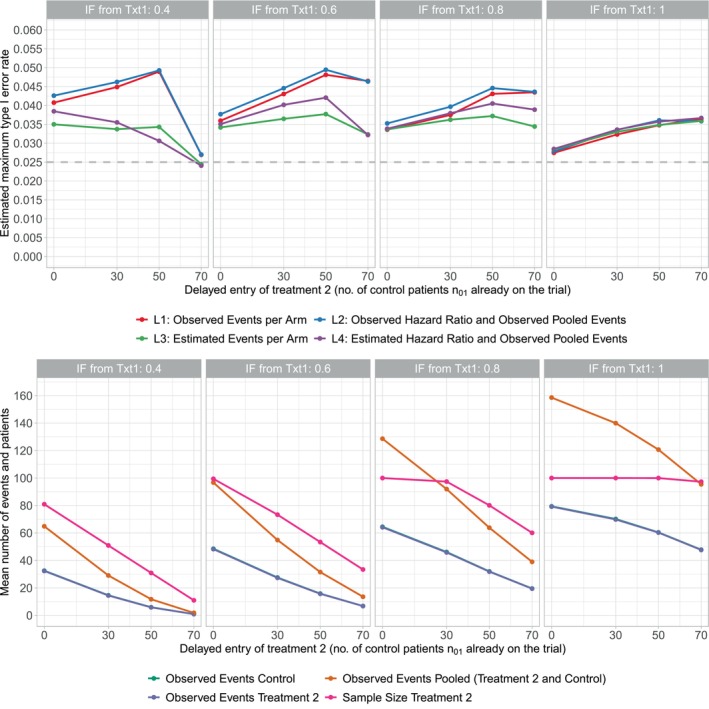
Top: Estimated maximum Type I error rate for the comparison of Treatment 2 and the control using the approximate adaptation rule (Equation (10)) when results of Treatment 1 against the shared control at an interim analysis (information fraction [IF] 0.4, 0.6, 0.8), or at the primary analysis (information fraction 1) (see grids) of Treatment 1 is published, and delayed entry of Treatment 2 (*x*‐axis). Entry at 0 denotes an immediate start of Treatment 2 (i.e., all arms start at the same time), an entry at 70 denotes a late entry. Bottom: Mean number of events in the control or Treatment 2, as well as pooled (control and Treatment 2). Mean number of patients recruited to Treatment 2 at the time of analysis of Treatment 1.

The estimated maximum Type I error rate for the comparison of treatment arm 2 and the control arm using the approximate adaptation rule (Equation (10)) for the four different types of information leakages when Treatment 1 reaches an interim or primary analysis (i.e., information fraction for Treatment 1 is 100%) is shown in Figure 3 in the top panel as a function of varying time points of Treatment 2 joining the platform. All information leakage L1 to L4 to decide for an ad‐hoc efficacy analysis lead to an inflation of the Type I error rate, with the highest inflation obtained when both the observed hazard ratio and number of events are available (L2, blue line). In all cases, the maximum Type I error rate increases with the delayed entry of Treatment 2 into the platform as less information for Treatment 2 and the shared control is available. The latter is of particular importance when outcome results were estimated based on leaked data (e.g., ${\tilde{d}}_{2}\left(1\right),{\tilde{d}}_{0_{\left[2\right]}}\left(1\right)$ or ${\tilde{\theta }}_{2}\left(1\right)$ in L3, green line and L4, purple line). The amount of information (i.e., the number of events in the control and treatment arm, as well as the sample size in Treatment 2) for each delayed entry of Treatment 2 and information leakage time point is provided in the bottom panel of Figure 3. With a late entry of Treatment 2 to the trial, or an early interim analysis of Treatment 1, lower amount of information is available. Note that under the global null, the number of events in the control and treatment arm are the same, thus the purple and orange lines are overlapping in the graph. With low amount of information from Treatment 2, additional rejections at the ad‐hoc analysis are possible (which would not have been rejected at the primary analysis). When both treatments start at the same time (i.e., delayed entry of Treatment 2 is 0), each treatment will reach the primary analysis first in approximately 50% of cases under the global null ($\theta_{1}=\theta_{2}=1$). In cases, when Treatment 2 reaches the primary analysis first, no information leakage happens leading to only slight Type I error inflation (note that the maximum Type I error rate conditioned that the primary analysis of Treatment 2 happens later would be actually higher). Figure S2 in the Supporting Information displays the frequency of when Treatment 2 has its primary analysis after the analysis of Treatment 1 (i.e., a leakage is still possible). Furthermore, Figure 3 shows the maximum Type I error rate (top panel) and the amount of information available (bottom panel) when Treatment 1 reaches 40%, 60% and 80% of events (i.e., interim analyses of Treatment 1 at information fraction [IF] 40%, 60% and 80% of events) resulting in an earlier information release. The maximum Type I error rate is furthermore increased with early interim analyses of Treatment 1. Similarly, less information is available for Treatment 2 at the early time points allowing for additional rejections at the ad‐hoc analysis which would have not been rejected at the primary analysis. However, in some cases almost no information is available for Treatment 2, which in contrast reduces the maximum Type I error rate close to the significance level since no leakage is possible. For example at interim analysis of Treatment 1 at 40% information (left grid) and a delayed entry of Treatment 2 after 70 patients (*x*‐axis), recruitment of Treatment 2 just started and thus, often no events have been observed so far. Overall, from Figure 3 it can be seen that the Type I error rate inflation is dependent on the amount of information available for Treatment 2, the type of information used for trial adaptation, and the time point when the analysis is performed (i.e., how far the ad‐hoc and primary analysis are apart). A large amount of information for Treatment 2 allows the experimenter to precisely estimate the treatment effect and log‐rank test statistic. This increases the maximum Type I error. On the other hand, with a large amount of information, the overlap of information for the ad‐hoc analysis and the planned primary analysis for Treatment 2 increases. This reduces the possibility for additional rejections at the ad‐hoc analysis as the two analysis time points are close and therefore decreases the maximum Type I error. Consequently, the two aspects have opposing effects on the maximum Type I error rate and further discussion is provided in the next subsection. Since the presented Type I error rate is dependent on the analysis time point of Treatment 1, we also evaluated scenarios with varying effect of Treatment 1 (moderate and high effect) in the Supporting Information Section A. Higher effects for treatment arm 1 mean less events for treatment arm 1 and therefore more time is needed to reach the pre‐specified number of events. Slight differences in the inflation are observed but the overall pattern is the same as under the global null and confirm the discussions below on the two aspects affecting the maximum Type I error rate. The data for the results above were generated using an exponential distribution. Simulations using alternative methods for data generation (i.e., multi‐state model and delayed treatment effects) are provided in the Supporting Information in Section A3. We observed a similar inflation pattern as with the exponential distribution, which aligns with the findings so far. The similarity is likely due to the fact that the assumptions for the median times using the alternative distributions were chosen such that the overall median would be approximately the same as when using the exponential distribution.

#### 4.1.1 Amount and Type of Available Information

Depending on what type of information is used for the decision of an ad‐hoc analysis, the maximum Type I error rate is affected differently. In general, a higher Type I error rate is observed, when the leaked information allows for a precise estimation of the underlying treatment effect or the test statistic. A high correlation between the estimated log‐rank test statistics using leaked information and the observed log‐rank test statistic at the ad‐hoc analysis results in a higher maximum Type I error. It is clear that the correlation between the estimated log‐rank test statistic using L2 (observed hazard ratio and observed pooled events) and the observed log‐rank test statistic at the ad‐hoc analysis is 1 as all data needed for the log‐rank test statistic is available (i.e., ${\tilde{Z}}_{2,L2}\left(1\right)\approx {\hat{Z}}_{2}\left(1\right)$). For the estimations based on leaked data in L3 and L4 used for trial adaptation, more information allows for a better estimation and therefore the correlation of the observed log‐rank test statistic and estimated statistics based on leaked data increases. This correlation can be considered a surrogate for the degree of unblinding.

#### 4.1.2 Amount of Available Information and Distance Between Two Analysis Time Points

Nevertheless, we have seen that with increasing amount of information (i.e., when the ad‐hoc analysis t=1 moves closer to the primary analysis t=2), the Type I error rate inflation is smaller as compared to scenarios with lower amount of information. With higher amount of information, the resulting estimated log‐rank test statistic based on leaked data at time t=1 will be closer to the observed log‐rank test statistic at the primary analysis t=2, which has a Type I error rate of 2.5%. The low amount of information for Treatment 2 results in a lower correlation of the estimated log‐rank test statistic based on the leaked data and the primary log‐rank test statistics and increases the possible number of rejections at the ad‐hoc analysis t=1 which would have not been rejected at analysis t=2.

Overall, two opposing effects are observed with increasing amount of information for Treatment 2, that is, how well is the log‐rank test statistic at analysis t=1 represented by the type of information, and how much information has been accumulated so far. A combination of these two effects determines the amount of error rate inflation. The exact relationship of the type and amount of information on the maximum Type I error rate is however out of scope for this publication and requires further extensive simulations varying multiple parameters. Nevertheless, selected simulations varying the treatment effect of Treatment 1, and varying the median survival under the global null are provided in the Supporting Information Section A which substantiate the discussion on the two opposing effects.

### 4.2 Bias When Estimating the Maximum Type I Error Rate

**FIGURE 4:**
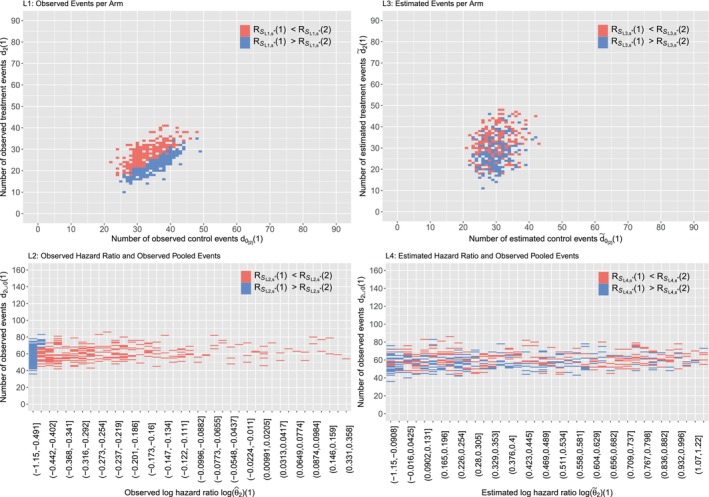
Tile graph comparing the conditional Type I error rate for the tests that stop at the ad‐hoc analysis t=1 and the primary analysis t=2 given L1, L2, L3 and L4 when Treatment 2 joins the platform after 50 patients (50% of control patients). Ad‐hoc analysis t=1 is triggered by Treatment 1 against the shared control at an interim analysis of IF at 80%. Cases of equal conditional Type I error rates are omitted.

To evaluate the bias when estimating the maximum Type I error rate using Equation (12), we defined a simplified adaptation rule using an LDA for L1, L3 and L4 and using the log‐rank test statistic for L2. Our approach to determine a simplified rule based on a LDA is solely based on pragmatism. To determine the optimal separation $R_{S_{L,s^{*}}}\left(1\right)>R_{S_{L,s^{*}}}\left(2\right)$ from the rest is out of scope here and would require further extensive simulations. With our simplified decision rule, we estimate the approximated maximum Type I error rate as best as possible and determine the potential bias observed in our simulation while acknowledging the shortcomings of the approach. In Figure 4 the estimated conditional error rates $R_{S_{L,s^{*}}}\left(1\right)$ and $R_{S_{L,s^{*}}}\left(2\right),L\in \left\{L1,L2,L3,L4\right\}$ for the tests that stop at analysis t=1 and t=2 are displayed as tiles in relation to the leaked data L. In this graph, Treatment 2 joins the platform after 50 patients and analysis t=1 is triggered when Treatment 1 reaches an IF of 80%. Tile graphs for other delayed entries and analysis time points are provided in the Supporting Information Section B.3. Blue tiles denote when $R_{S_{L,s^{*}}}\left(1\right)>R_{S_{L,s^{*}}}\left(2\right),L\in \left\{L1,L2,L3,L4\right\}$. Tile graphs with an overlay of the resulting LDA on the tile graphs are presented in the Supporting Information Section B.5. For L1 (upper left panel) a rather clear separation of the blue and red tiles can be seen, but for L3 and L4 (upper and lower right panels) a higher variety of pairs and noise is observed due to the estimation of the data.

For L2, the separation of the coloured tiles results from the condition that $(-\sqrt{d_{0_{\left[2\right]}}\left(1\right)+d_{2}\left(1\right)}\cdot 0.5\cdot \log{\left({\hat{\theta }}_{2}\left(1\right)\right)}^{2}>\chi_{1,0.95}^{2}$ and ${\hat{\theta }}_{2}\left(1\right)<1$ (for a one‐sided test) which follows Equation (3). Consequently, the simplified adaptation rule leading to a separation of the blue and red tiles is obtained when experimenters decide to stop for an ad‐hoc analysis if the leaked information allows a rejection of the null hypothesis. Full details on the adaptation rules are provided in the Supporting Information Section B.

**FIGURE 5:**
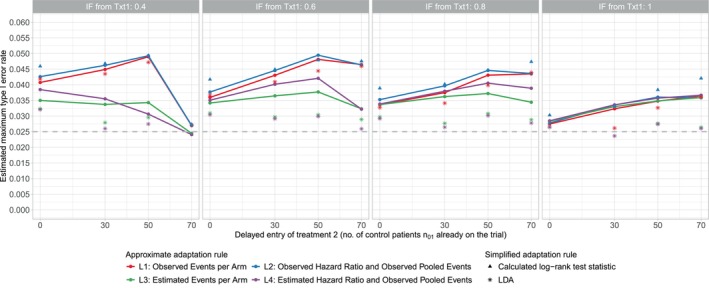
Estimated maximum Type I error rate for the comparison of Treatment 2 and the control using the approximate adaptation rule (Equation (10)) when results of Treatment 1 against the shared control at an interim analysis (information fraction [IF] 0.4, 0.6, 0.8), or at the primary analysis (information fraction 1) (see grids) of Treatment 1 is published, and delayed entry of Treatment 2 (*x*‐axis). Entry at 0 denotes an immediate start of Treatment 2 (i.e., all arms start at the same time), an entry at 70 denotes a late entry. An additional simulation was performed using the simplified adaptation rule determined by the LDA (stars), or using the log‐rank test statistic (triangle). If the maximum Type I error rate is indeed 0.05, then the simulation error using the simplified adaption rule is $\sqrt{0.05\cdot 0.95/10^{6}}=0.00022$.

In Figure 5 we display the approximate maximum Type I error rate using the approximate adaptation rule Equation (10) as shown in Figure 3 and added the Type I error rate using the simplified adaption rule as symbols: stars denote the rule using the LDA and triangles denote the rule using the log‐rank test statistic. In general, for L1 (red curves and red stars), a good approximation of the estimated maximum Type I error rate can be observed. The Type I error rate using the simplified adaptation rule is either matching or slightly lower than the estimated maximum Type I error rate using the approximate adaptation rule in most cases. Discrepancies may be attributed to the LDA predictions and noise from the simulation. Especially, when analysis t=1 is performed at IF 1 of Treatment 1. The corresponding tile graph in the Supporting Information Section B.3 shows that a clear separation using the LDA appears difficult and may not correspond to the optimal separation of the tiles.

On the contrary, for L2 (blue curves and blue triangles), we see that in most cases the estimated maximum Type I error rate using the approximated adaptation rule can be matched by the simplified adaptation rule. In some cases, the estimated maximum Type I error rate using the simplified adaptation rule was even higher than using the approximated adaptation rule. This was due to the fact that the latter rule is dependent on the number of occurrences of each unique pair $D_{L,s^{*}}=\left(d_{2\cup 0}\left(1\right),\text{Categorised}\;{\hat{\theta }}_{2}\left(1\right)\right)$ and especially the classification of the hazard ratios into bins using quantiles, which affects the estimated maximum Type I error rate. To evaluate the impact of the choice of the categorisations of the hazard ratio, we further estimated the maximum Type I error rate using an alternative definition of bins. The estimated maximum Type I errors using these alternative bins are similar or slightly higher than with the bins based on quantiles. The overall trend remains the same (see Supporting Information Section B.7).

For both, L3 and L4 we see that the tile graphs in Figure 4 (right panel) show a high amount of noise. In comparison to the observed number of events per arm (L1) or the observed total number of events and the observed hazard ratio (L2), a clear pattern for an adaptation rule is difficult to obtain potentially due to several reasons. Clearly, the estimated number of events per arm and estimated hazard ratio are dependent on the assumptions made for estimation and information available for the estimation. Several estimation steps are involved which increases the variability. The approximation of the conditional error rate is dependent on the frequency in which the estimated pairs were obtained. Therefore, the resulting LDA for L3 and L4 cannot completely separate the coloured tiles (Figures S21 and S22 in the Supporting Information). This results in a Type I error rate using the simplified adaptation rule much lower than the estimated maximum Type I error rate using the approximate adaptation rule (Equation (10)). The adaptation rule based on the LDA was not optimised to find the best separation. Further optimisation of the rule to better separate the tiles may increase the Type I error rate closer to the maximum Type I error rate.

Overall, this exercise shows that when using the approximate adaptation rule (Equation (10)), on the one hand a positive bias is induced, leading to a potential overestimation of the maximum Type I error. For L2 and L4, an opposing effect is expected in addition to the positive bias due to the categorisation of the hazard ratios. When using the simplified adaptation rule for L2, we see a higher maximum Type I error rate compared to the approximate adaptation rule as the observed hazard ratio is directly used in the adaptation rule as opposed to the average hazard ratio in a category when using the approximate adaptation rule. This effect could be reduced when using alternative bins for the categorisation of the hazard ratio (see Supporting Information Section B.7). For L1, L3 and L4, the simplified adaptation rule used an LDA, which may not have been optimal. Nevertheless, the resulting Type I error rate can be considered a lower bound for the maximum Type I error rate as it approximates the estimated maximum Type I error rate using the approximated adaptation rule.

## 5 Discussion

In this paper, we have investigated the potential information leakage in event‐driven platform trials and the resulting maximum Type I error rate. We evaluated four different scenarios of information leakage covering both access to the observed interim data, for example, the estimated hazard ratio from the trial data and estimated comparative results, for example, the estimated hazard ratio based on leaked data. The latter is possible by combining released results from other arms on the platform trial and data from regular monitoring. Even pooled data such as event counts pooled between the active and the control arm might release information that allows to estimate the event rate in another (concurrent) arm. This is different from other types of trial.

The simulations showed that the amount of Type I error inflation depends on the type of information that is leaked, how well the log‐rank test statistic can be predicted based on that information, and on the time point of information leakage. The timing is relevant as this affects the amount of information available and thus the variability of the recalculations. Here we have two opposing effects. On the one hand, it is good to have as much information from Treatment 2 as possible in order to estimate the treatment effect and log‐rank test statistic as precisely as possible to maximize the Type I error rate (and thus the chance of a successful trial). On the other hand, the two analysis time points should be far enough apart, as the rejection regions from the two analysis time points become more divergent and thus can lead to a higher inflation of the Type I error rate. There is no inflation if almost no or almost all data of Treatment 2 have been collected at analysis t=1. The simulation results presented in Section 4 were obtained under the global null hypothesis, that is, when no treatment has an effect, with a median survival time of 5 months for all arms. In additional simulation scenarios provided in the Supporting Information we varied the hazard ratio of Treatment 1 (see Supporting Information, Section A.1), and varied the median survival under the global null (see Supporting Information, Section A.2) and have observed these opposing effects as well. When varying the treatment effect in Treatment 1, we see that in cases when Treatment 1 is very effective (i.e., low hazard ratio) a lower maximum Type I error inflation is observed for Treatment 2 as it takes longer until the required number of events for the analysis of Treatment 1 are reached. In that case also more events and information for Treatment 2 are obtained until time t=1. This leads to a higher similarity of the data used in the ad‐hoc interim analyses and the pre‐planned primary analysis of Treatment 2, reducing the possibility of additional rejections. Similar opposing effects were seen under the global null with varying median survival times when Treatment 2 joins the platform at later time points (see Supporting Information, Section A.2). With a higher median survival, it takes longer until the required number of events for the analysis of Treatment 1 is reached allowing for more information to accrue for Treatment 2 at analysis t=1 for trial adaptation.

In all simulated cases an inflation in Type I error rate was observed, with the highest inflation when the actual hazard ratio and the number of events for Treatment 2 are available at analysis t=1. In this case, the log‐rank test statistic can be approximated and the experimenter is fully unblinded. Less inflation was observed when comparative results used for trial adaptation are estimated based on other leaked data, as the estimation relies on assumptions.

Throughout the manuscript, we solely evaluated the marginal Type I error rate for the comparison of Treatment 2 and the control but an inflation of the marginal Type I error rate also leads to an inflation of the study‐wise Type I error rate. A strict control of the Type I error rate across Treatment arms was not in scope and may, in practise, also not always be necessary or relevant for complex clinical trials as mentioned in the FDA's draft guidance on master protocols [9]. It mentions that generally no multiplicity adjustment across multiple comparisons of different treatment arms to the control group in umbrella or platform trials is recommended. However, exceptions exist, for example, in case of multiple doses of the same drug. This is also in line with other discussions on the need for multiplicity adjustment across arms in platform trials [14, 28]. An EMA guideline on platform trials is pending [7]. Importantly, while strict Type I error control across treatment arms may not be necessary in complex clinical trials, the strict Type I error control due to other sources such as multiple endpoints and interim analyses for each individual treatment compared to the control is still mandatory in confirmatory trials and general methodological guidelines apply [9].

Firewalls are often implemented in clinical trials to avoid unblinding. These usually prevent observing interim data from the arm of interest itself. However, in platform trials, naturally, information on the shared control will be obtained when other treatments release results. In particular, event‐driven platform trials provide even more information, such as the number of events. This allows an estimation of comparative results even without observed interim data for the arm of interest. While unplanned interim analyses driven by external factors usually do not require an adjustment on the Type I error rate, the potential leakage of data in platform trials raises concerns, as it is less clear if decisions were made based on external evidence only. In the worst case, experimenters could use estimated results to decide to stop for an ad‐hoc efficacy analysis but claim that the decision was driven by external data or by operational reasons. This possibility makes it difficult to verify whether decisions were data‐driven or not. Both for experimenters and regulators, a risk in both directions exists. A summary of sources of data leakage and potential measures to prevent or reduce the leakage is provided in Section 6.

Clearly, our simulation and estimation of comparative results rely on many assumptions, which in practise will not be necessarily fulfilled. Missing data and censoring will affect the amount of information available for estimation and thus the information leakage may be less clear than assumed in our simulations. We assumed no missing data and applied administrative censoring only, that is, follow up times were censored at the time of analysis. The analysis of the impact of missing data and other censoring patterns on the Type I error was not in scope of this manuscript. It merely introduces another layer of complexity and results in a multitude of additional simulation scenarios. As the main aim of this manuscript was to raise awareness of the potential issue of information leakage, we focussed on varying other essential variables only. Nevertheless, future research on this may be of interest.

In addition, the maximum Type I error rate is only reached when experimenters are able to obtain the leaked information on the hazard ratio, which usually is unlikely in the case of well conducted blinded trials. This *could* reduce the potential impact of data leakage in practise. However, accidental unblinding can occur in all trials, and potential counter measures and recommendations are provided in Section 6.

We approximated the maximum Type I error rate using the approximated adaptation rule by taking the maximum of two estimates (see Algorithm 1) which may overestimate the actual maximum error rate. On the other hand we used categorisations for the hazard ratio, which is necessary to calculate the approximated maximum Type I error rate (see Section 3.1) when using continuous data, which has an opposing effect resulting in a negative bias. In additional simulations we evaluated the bias resulting from using the approximated adaptation rule. The approximation of the estimated maximum Type I error rate using the simplified adaptation rule can be considered a lower bound for the maximum Type I error rate. Importantly, we did not aim to determine an ‘optimal’ rule to maximize the Type I error rate. Thus, the approximated adaptation rule should only be viewed as a pragmatic approximation. Overall, the simulation results should not be treated as an exact quantification of the maximum Type I error but should rather show that information leakage can in principle result in an inflation of the Type I error rate. It is also noteworthy, that not only the maximum Type I error rate is affected by the different types of information leakage. Clearly, with an earlier analysis t=1 the power for the comparison of Treatment 2 to control can decrease in the ad‐hoc analysis for Treatment 2 as they are performed with less events (Supporting Information, Section C).

We only considered two treatments and a control arm for our manuscript. In practise, more treatment arms may be included in a platform trial. It is important to note that an increased number of treatment arms will not change the results from scenarios L1 and L2 as these use the observed leaked data (number of events and hazard ratio) from the arm of interest and independent of the leaked data from other arms on the trial. It potentially impacts scenarios L3 and L4, though, as it increases the available information, which can be used to estimate the number of events or the hazard ratio. In case of a high overlap of control patients between the treatment arms, the additional information may help to increase the precision of these estimates. While the extension to more treatment arms would be of interest for the latter scenarios, it strongly increases the complexity of the simulations as additional considerations on the type and timing of the leaked data and the potential use of the leaked information would be needed. For example, the data leakage could occur at the same or at different times. Furthermore, the amount of leaked data could vary such that only the accrual and follow up information, the number of events, the median times, or a combination thereof may be available at different time points for different treatment arms. Thorough considerations on the potential combination of the amount of leaked information and time point of information leakage would be needed in that case. As repeatedly stated, this was not in scope here. The main objective was to show whether information leakage is an issue in platform trials rather than provide guidance on how to ‘optimally’ use leaked information. Using only two treatment arms is considered sufficient to show that information leakage has an effect on the maximum Type I error rate. The extension to more treatment arms may be evaluated in the future.

While we have focused on the impact of information release on the Type I error rate in platform trials, an interesting aspect is also observed under the alternative: Assuming two treatments and a control start at the same time. If Treatment 1 reaches the required number of events showing a significant treatment effect against the common control, then Treatment 2 can conclude that it will also be superior to the control group as in total less events were observed, which can only be due to the active arm.

Overall, this paper should raise awareness of the potential for estimation of comparative results based on leaked data in platform trials and hence the possibility for post hoc trial adaptations. To this end, we investigated the maximum Type I error rate inflation. However, information leakage does not only affect the Type I error rate but might introduce bias and also affects further trial design aspects such as recruitment speed, change in patients recruited, or compliance with the study protocol.

## 6 Recommendations

### 6.1 Avoiding Data Leakage in Platform Trials

While malicious breach of firewalls to obtain leaked data is considered unlikely in well conducted clinical trials, data leakage and (accidental) unblinding can occur in all trials. There is an increased number of sources and a higher likelihood for data leakage in platform trials. Therefore, platform trials require special care and detailed pre‐specification and safeguards for the trial integrity such as the use of independent committees or restricted access to data. This is of particular importance if the trial is confirmatory. A short summary of the sources of unblinding or data leakage and the proposed measures to prevent these are provided in Table 3. A detailed discussion is provided in the following paragraphs.

**TABLE 3:** Overview of the main sources of unblinding or data leakage in platform trials and the potential measures to prevent or reduce these sources.

Sources of unblinding or data leakage	Measures to prevent or reduces leakage
1. Data leakage for the arm of interest	
Number of events per arm Hazard ratio	Restrict access to data for study team (firewall) Use of iDMCs Use independent decision‐making teams with only the minimum needed personnel Use of independent study teams per sub‐study
2. Data leakage through monitoring information	
Pooled number of events of each arm and its concurrent controls Sample sizes per arm (including information on the amount of overlap of controls) Accrual and follow‐up information	Avoid reporting and sharing operational information which has the potential to estimate effects in ongoing arms. For example, instead of reporting the pooled number of events, only report the projected analysis times per arm and update these times only when ultimately needed. Keep the reporting of operational information to the minimum needed, for example, if possible blind the information on the overlap of control patients
3. Information leakage on shared control arm when other treatment arms share results	
Number of events in control arm Median survival rate in control arm Hazard rate in control arm	Reduce sharing of individual efficacy results for the control group. Only report comparative summary results (e.g., difference between groups or hazard ratios) Publish results only when and if needed. Find a good balance between publication of results (time and content) and maintaining blinding for other arms, that is, find a reasonable compromise between transparency and trial integrity.

#### 6.1.1 Data Leakage for the Arm of Interest

The usual firewall to avoid information leakage in trials is to restrict and manage the access of study team personnel to all critical trial data. Otherwise, knowledge of leaked data such as in scenarios L1 and L2, where observed results from Treatment 2 were available to the core study team even though this information should be blinded, can introduce bias in the remaining trial conduct, risk the trial integrity and interpretability of the results. Furthermore, following guidance from FDA [9] and EMA [7], an independent, external data monitoring committee (iDMC) to monitor efficacy and safety data is recommended.

In some cases, developers would like to include internal representatives in the decision‐making either as part of the iDMC or in a separate decision‐making board for, for example, strategic internal planning of the development programme. A clear firewall should be in place to safeguard independence of decision making. The number of internal representatives exposed to the trial data for pre‐planned decision making of trial adaptations should be kept at a minimum and these individuals should not be involved in the study team [33, 34]. The study team overlooks the daily work of the clinical trial and remains blinded until the blind is officially broken [35]. Since multiple study arms are evaluated in platform trials, if sufficient resources are available, independent study teams per sub‐study should be considered in addition to the iDMC to further separate the available information as good as possible. The roles, responsibilities and the access to the study data should be pre‐defined and documented before the study starts for all the different teams and boards.

#### 6.1.2 Data Leakage Through Monitoring Information

In a platform trial with a shared control and event‐driven analyses, combining operational data with information on the common control from efficacy analyses of another arm allows for an estimation of comparative results (see scenarios L3 and L4). This is in particular possible because the total number of events is usually monitored in event‐driven trials by the study team to plan for the primary analysis. This requires additional measures beyond restricting access to unblinded data.

In terms of operational data such as the sample sizes per arm, the accrual and follow‐up information or the information on the amount of overlap of control patients, the regular reporting of these parameters to the study team should be kept to a minimum as they could potentially allow to infer comparative results if combined with additional information. Only selected members of the study team that require the information, for example, for data cleaning purposes or managing the drug supply, should have access.

Where possible, avoid continuous or frequent reporting of the number of pooled events to the study team, which is usually done to determine the analysis time point. If pooled event counts $d_{j\cup 0}$ are frequently reported, one can estimate the number of events per arm, if at least two active arms j enrol new patients at any given time. In a multi‐arm design were all arms start (and end) recruitment at the same time, pooled event counts allow the exact computation of all group specific event counts. This gets slightly more complicated if arms only overlap partially but is still possible to some extend. Hence, rather than reporting the actual number of events to the study team, only the projected time point of primary analysis should be reported. Furthermore, a risk assessment should be performed for individual operational parameters (e.g., continuous accrual information, number of pooled events) by evaluating the risk for a potential unblinding when reporting these operational parameters against the operational burden by keeping these parameters blinded to the full or partial study team. This assessment including the measurements for keeping the blind of the operational parameters for (individual) study team members should be performed and documented prior to study start in a firewall charter.

#### 6.1.3 Information Leakage for Common Control Arm When Other Treatments Share Results

Lastly, in platform trials, results and information on the common control may become available and might be misused to change trial in a data‐driven manner when analyses are performed for one arm, while other arms are still on the trial. In some cases, immediate communication of the results is unavoidable, for example, in case of ad‐hoc notifications for the stock exchange. In that case it is advisable to keep the sharing of individual results of the control group to a minimum. Thus, rather than reporting the median survival or hazard rate in the control group (or the active arm), report only the hazard ratio between the active and control arm. In certain situations, a delay in reporting of results may be possible and reasonable and supports the trial integrity. While trial results should in general be reported as early as possible and in conjunction with legal and ethical requirements, it might be advised to keep results confidential for a specific time to allow other arms to mature or complete. An assessment on the timing of reporting results and its risk to trial integrity should be conducted prior to study start including a communication plan that defines which results will be reported when and to whom. The EMA Q&A document [7] provides recommendations on the balance between transparency (reporting of trial results) and maintaining the trial integrity.

### 6.2 Measures to Reduce the Impact of Leakage

In any case, clear and unambiguous pre‐specification of all analyses (timing and conduct), who has access to which data and when will which information be released or communicated to whom is of utmost importance. These aspects should be pre‐specified preferably in the study protocol and might be complemented with further details in the statistical analysis plan or other trial documents such as the iDMC charter or the communication plan. Adherence to the plans is crucial in complex clinical trials, including platform trials, as these leave more room for inadvertent data leakage, which could meaningfully impact the robustness of the data as shown in this manuscript. Examples of aspects to be specified for complex clinical trials are defined in EMA's Q&A document [7]. These include, for example, the definition of end of trial, or end of sub‐protocol, a communication plan, and the role of iDMCs. All these aspects describe measures to reduce the risk of information leakage to trial integrity.

We want to emphasise the fact that in platform trials with a common control and event‐driven analyses, the inadvertent dissemination of information may still be present although operational measures have been implemented. As seen in the simulation, an estimation of efficacy results based on leaked data is possible, though many assumptions are required. Thus, if not pre‐specified, any unplanned interim analysis or design adaptation could be (mistakenly or correctly) seen as data‐driven. Two options could be pre‐specified to minimize the potential impact of the release of information on Type I error rate and bias:

- *Prohibit Adaptations and Unplanned Analyses:* No interim analyses or adaptations to ongoing arms are allowed if not pre‐specified in the protocol, where pre‐specification means that the design aspects are specified before the enrolment to that arm starts. While we have focused in our simulations on early efficacy stops, any desired trial adaptation such as sample size recalculations or changes to endpoints should be pre‐specified. In this case, no doubt would be present that decisions to perform an analysis or adaptation were a post hoc, data‐driven decision. This would also prohibit any unplanned analysis or adaptations triggered by external events such as recruitment issues, results from other trials or similar and hence might not always be practical.
- *Force ad‐hoc Interim Analyses:* Assuming that some degree of unblinding is present, all analyses of treatment arms on the platform will automatically trigger pre‐planned interim analyses for all ongoing arms in the trial. By adjusting for these pre‐planned analyses one can avoid the risk of post hoc, data‐driven analyses. Classical group‐sequential methods such as α‐spending function approaches [36] could be implemented to assign significance levels at interim analyses according to the information fraction observed so far (e.g., using spending functions which mimic O'Brien‐Fleming or Pocock boundaries). Alternatively, also administrative α‐spending methods such as the Haybittle‐Peto rule [37, 38] might be considered, which spends a small administrative α at each ad‐hoc interim analysis and use an adjusted α which is close to the full significance level at the planned primary analysis. It is noted that it is not considered acceptable to use the full significance level at the primary analysis when using administrative α‐spending as this would lead to an increase in Type I error rate. Given the potentially many triggered analyses, the magnitude of inflation may be further increased. As mentioned before, all details of these analyses including spending functions and planned adaptations need to be defined prospectively before the corresponding arm is opened for enrolment. With increasing number of treatment arms and thus number of potential interim analyses, the operational burden of such an approach increases as timely data entry, data monitoring, data cleaning and also data analysis are required for all arms.

Depending on the time point of joining the platform and time point of information releases triggered by other treatments, each option should be carefully considered. For example, if an arm plans to join the platform rather late and an analysis of another arm would be a few weeks later, pre‐specification that no interim analysis will be performed might be a reasonable solution as the data will be very immature and information leakage for the new arm will be minimal anyhow. To this end, Asikanius et al. [39] proposed to allow an analysis to be performed only after all arms completed the recruitment when designing a three‐arm trial with two treatments and a common control. This example shows that further aspects or conditions could be specified in the protocol on when a forced interim analysis should be foreseen. Conditioning on the recruitment, one could pre‐specify that a forced pre‐planned interim analysis will only be performed for the arm of interest at the time of another arm's primary analysis if all planned treatment and concurrent controls of the arm of interest have been recruited. Should the recruitment still be ongoing, the protocol should pre‐specify that no ad‐hoc adaptation and interim analysis would be allowed. Furthermore, if a primary analysis of one arm would be close to a planned interim analysis of another arm anyway, experimenters could consider moving the interim analysis to the same time point as the primary analysis and adjust for the analysis accordingly.

In summary, the key aspect is the pre‐specification of measures and actions in the protocol and additional study documents from (1) an operational point of view, for example, with the implementation of an iDMC, shielding the pooled number of events from the study team(s), independent study teams and data access plans, and (2) a statistical perspective, for example, pre‐plan if, when and what (adjusted) analyses are performed when other treatment arms release information.

## Author Contributions

All authors designed the research and drafted the article. Quynh Nguyen performed the simulations. All authors contributed to the interpretation of findings, critically reviewed and edited the manuscript.

## Funding

The authors have nothing to report.

## Ethics Statement

The authors have nothing to report.

## Consent

The authors have nothing to report.

## Conflicts of Interest

The authors declare no conflicts of interest.

## Supporting information


**Figure S1:** Top: Estimated maximum Type I error rate for the comparison of Treatment 2 and the control using the approximate adaptation rule when results of Treatment 1 against the shared control at an interim analysis (information fraction [IF] 0.4, 0.6, 0.8), or at the primary analysis (information fraction 1) (see grids) of Treatment 1 is published, and delayed entry of Treatment 2 (*x*‐axis). Entry at 0 denotes an immediate start of Treatment 2 (i.e., all arms start at the same time), an entry at 70 denotes a late entry. Bottom: Mean number of events in the control or Treatment 2, as well as pooled (control and Treatment 2). Mean number of patients recruited to Treatment 2 at the time of analysis of Treatment 1.
**Figure S2:** Frequency (%) that the pre‐planned analysis of Treatment 2 will take place after the first analysis of Treatment 1 (at information fraction [IF] 0.4, 0.6, 0.8.1).
**Figure S3:** Top: Estimated maximum Type I error rate for the comparison of Treatment 2 and the control using the approximate adaptation rule when results of Treatment 1 against the shared control at an interim analysis (information fraction [IF] 0.4, 0.6, 0.8), or at the primary analysis (information fraction 1) (see grids) of Treatment 1 is published, and delayed entry of Treatment 2 (*x*‐axis). Entry at 0 denotes an immediate start of Treatment 2 (i.e., all arms start at the same time), an entry at 70 denotes a late entry. Bottom: Mean number of events in the control or Treatment 2, as well as pooled (control and Treatment 2). Mean number of patients recruited to Treatment 2 at the time of analysis of Treatment 1. Treatment 1 with moderate effect (HR 0.833).
**Figure S4:** Top: Estimated maximum Type I error rate for the comparison of Treatment 2 and the control using the approximate adaptation rule when results of Treatment 1 against the shared control at an interim analysis (information fraction [IF] 0.4, 0.6, 0.8), or at the primary analysis (information fraction 1) (see grids) of Treatment 1 is published, and delayed entry of Treatment 2 (*x*‐axis). Entry at 0 denotes an immediate start of Treatment 2 (i.e., all arms start at the same time), an entry at 70 denotes a late entry. Bottom: Mean number of events in the control or Treatment 2, as well as pooled (control and Treatment 2). Mean number of patients recruited to Treatment 2 at the time of analysis of Treatment 1. Treatment 1 with high effect (HR 0.625).
**Figure S5:** Top: Estimated maximum Type I error rate for the comparison of Treatment 2 and the control using the approximate adaptation rule for Treatment 2 when results of Treatment 1 against the shared control at an interim analysis (information fraction [IF] 0.4, 0.6, 0.8), or at the primary analysis (information fraction 1) (see grids) of Treatment 1 is published, and delayed entry of Treatment 2 (*x*‐axis). Entry at 0 denotes an immediate start of Treatment 2 (i.e., all arms start at the same time), an entry at 70 denotes a late entry. Varying treatment effects of Treatment 1 are displayed in different shapes. For the sake of simplicity and convenience, the different hazard ratios for treatment 1 are additionally labelled for selected results in selected grids only. The results for the global null as in the main paper are displayed with connecting lines. Bottom: Mean number of events in the control or Treatment 2, as well as pooled (control and Treatment 2). Mean number of patients recruited to Treatment 2 at the time of analysis of Treatment 1. Varying treatment effects of Treatment 1 are displayed in different shapes. For the sake of simplicity and convenience, the different hazard ratios for Treatment 1 are additionally labelled for selected results in selected grids only. The results for the global null as in the main paper are displayed with connecting lines.
**Figure S6:** Frequency (%) that the pre‐planned analysis of Treatment 2 will take place after the first analysis of Treatment 1 (at information fraction [IF] 0.4, 0.6, 0.8.1). Varying treatment effects of Treatment 1 are displayed by different symbols. For the sake of simplicity and convenience, the different hazard ratios for Treatment 1 are additionally labelled for selected results in selected grids only. The results for the global null as in the main paper are displayed with connecting lines.
**Figure S7:** Top: Estimated maximum Type I error rate for the comparison of Treatment 2 and the control using the approximate adaptation rule for Treatment 2 when results of Treatment 1 against the shared control at an interim analysis (information fraction [IF] 0.4, 0.6, 0.8), or at the primary analysis (information fraction 1) (see grids) of Treatment 1 is published, and delayed entry of –Treatment 2 (*x*‐axis). Entry at 0 denotes an immediate start of Treatment 2 (i.e., all arms start at the same time), an entry at 70 denotes a late entry. Varying median survival for all treatments under the global null are displayed in different shapes. For the sake of simplicity and convenience, the different medians are additionally labelled for selected results in selected grids. The results for the global null as in the main paper are displayed with connecting lines. Bottom: Mean number of events in the control or Treatment 2, as well as pooled (control and Treatment 2). Mean number of patients recruited to Treatment 2 at the time of analysis of Treatment 1. Varying median survival for all treatments under the global null are displayed in different shapes. For the sake of simplicity and convenience, the different medians are additionally labelled for selected results in selected grids. The results for the global null as in the main paper are displayed with connecting lines.
**Figure S8:** Frequency that the pre‐planned analysis of Treatment 2 will take place after the first analysis of Treatment 1 (at information fraction [IF] 0.4, 0.6, 0.8.1). Varying median survival for all treatments under the global null are displayed in different colours for a delayed entry of Treatment 2 after 50 patients in the control arm already recruited. For the sake of simplicity and convenience, the different medians are additionally labelled for selected results in selected grids. The results for the global null as in the main paper are displayed with connecting lines.
**Figure S9:** Top: Estimated maximum Type I error rate for the comparison of Treatment 2 and the control using the approximate adaptation rule when results of Treatment 1 against the shared control at an interim analysis (information fraction [IF] 0.4, 0.6, 0.8), or at the primary analysis (information fraction 1) (see grids) of Treatment 1 is published, and delayed entry of Treatment 2 (*x*‐axis). Entry at 0 denotes an immediate start of Treatment 2 (i.e., all arms start at the same time), an entry at 70 denotes a late entry. Bottom: Mean number of events in the control or Treatment 2, as well as pooled (control and Treatment 2). Mean number of patients recruited to Treatment 2 at the time of analysis of Treatment 1. Data generation for all arms following an exponential distribution (solid lines), or following an multi‐state (MS) distribution (dotted lines).
**Figure S10:** Top: Estimated maximum Type I error rate for the comparison of Treatment 2 and the control using the approximate adaptation rule when results of Treatment 1 against the shared control at an interim analysis (information fraction [IF] 0.4, 0.6, 0.8), or at the primary analysis (information fraction 1) (see grids) of Treatment 1 is published, and delayed entry of Treatment 2 (*x*‐axis). Entry at 0 denotes an immediate start of Treatment 2 (i.e., all arms start at the same time), an entry at 70 denotes a late entry. Bottom: Mean number of events in the control or Treatment 2, as well as pooled (control and Treatment 2). Mean number of patients recruited to Treatment 2 at the time of analysis of Treatment 1. Data generation for all arms following an exponential distribution (solid lines), or mixture where Treatment 1 follows an multi‐state (MS) distribution but Treatment 2 and the control follow an exponential distribution (dotted lines).
**Figure S11:** Top: Estimated maximum Type I error rate for the comparison of Treatment 2 and the control using the approximate adaptation rule when results of Treatment 1 against the shared control at an interim analysis (information fraction [IF] 0.4, 0.6, 0.8), or at the primary analysis (information fraction 1) (see grids) of Treatment 1 is published, and delayed entry of Treatment 2 (*x*‐axis). Entry at 0 denotes an immediate start of Treatment 2 (i.e., all arms start at the same time), an entry at 70 denotes a late entry. Bottom: Mean number of events in the control or Treatment 2, as well as pooled (control and Treatment 2). Mean number of patients recruited to Treatment 2 at the time of analysis of Treatment 1. Data generation for all arms following an exponential distribution (solid lines), or mixture where Treatment 1 follows an exponential distribution but Treatment 2 and the control follow a multi‐state (MS) distribution (dotted lines).
**Figure S12:** Top: Estimated maximum Type I error rate for the comparison of Treatment 2 and the control using the approximate adaptation rule when results of Treatment 1 against the shared control at an interim analysis (information fraction [IF] 0.4, 0.6, 0.8), or at the primary analysis (information fraction 1) (see grids) of Treatment 1 is published, and delayed entry of Treatment 2 (*x*‐axis). Entry at 0 denotes an immediate start of Treatment 2 (i.e., all arms start at the same time), an entry at 70 denotes a late entry. Bottom: Mean number of events in the control or Treatment 2, as well as pooled (control and Treatment 2). Mean number of patients recruited to Treatment 2 at the time of analysis of Treatment 1. Data generation for all arms following an exponential distribution (solid lines), or following an delayed distribution (dotted lines).
**Figure S13:** L1: Observed events per Arm. Tile graph comparing the conditional Type I error rate at the ad‐hoc analysis *t* = 1 and the primary analysis *t* = 2 given the observed number of events per arm. Row grids: Delay of Treatment 2 joining the platform after 0 patients (100% of control patients), 30, 50 and 70 patients in the control arm already on the trial. Column grids: Ad‐hoc analysis *t* = 1 is triggered by Treatment 1 against the shared control at information fraction 0.4, 0.6, 0.8 and 1 (primary analysis). Cases of equal conditional Type I error rates are omitted.
**Figure S14:** L2: Observed Hazard Ratio and Observed Pooled events. Tile graph comparing the estimated conditional Type I error rate at the ad‐hoc analysis *t* = 1 and the primary analysis *t* = 2 given the observed number of events and observed log hazard ratio. Ad‐hoc analysis *t* = 1 is triggered by Treatment 1 against the shared control at information fraction 0.4, 0.6, 0.8 and 1 (primary analysis). Cases of equal conditional Type I error rates are omitted.
**Figure S15:** L3: Estimated Events per Arm. Tile graph comparing the estimated conditional Type I error rate at the ad‐hoc analysis *t* = 1 and the primary analysis *t* = 2 given the observed number of events per arm. Row grids: Delay of Treatment 2 joining the platform after 0 patients (100% of control patients), 30, 50 and 70 patients in the control arm already on the trial. Column grids: Ad‐hoc analysis *t* = 1 is triggered by Treatment 1 against the shared control at information fraction 0.4, 0.6, 0.8 and 1 (primary analysis). Cases of equal conditional Type I error rates are omitted.
**Figure S16:** L4: Estimated Hazard Ratio and Observed Pooled events. Tile graph comparing the estimated conditional Type I error rate at the ad‐hoc analysis *t* = 1 and the primary analysis *t* = 2 given the observed number of events and estimated log hazard ratio. Ad‐hoc analysis *t* = 1 is triggered by Treatment 1 against the shared control at information fraction 0.4, 0.6, 0.8 and 1 (primary analysis). Cases of equal conditional Type I error rates are omitted.
**Figure S17:** L1: Observed events per Arm. Smoothed tile graph comparing the estimated conditional Type I error rate at the ad‐hoc analysis *t* = 1 and the primary analysis *t* = 2 given the observed number of events per arm. Tiles represent the original tiles. Dots are overlayed showing the smoothed tiles using the smoothed decision indicator. Row grids: Delay of Treatment 2 joining the platform after 0 patients (100% of control patients), 30, 50 and 70 patients in the control arm already on the trial. Column grids: Ad‐hoc analysis *t* = 1 is triggered by Treatment 1 against the shared control at information fraction 0.4, 0.6, 0.8 and 1 (primary analysis). Cases of equal conditional Type I error rates are omitted.
**Figure S18:** L3: Estimated events per Arm. Smoothed tile graph comparing the estimated conditional Type I error rate at the ad‐hoc analysis *t* = 1 and the primary analysis *t* = 2 given the estimated number of events per arm. Tiles represent the original tiles. Dots are overlayed showing the smoothed tiles using the smoothed decision indicator. Row grids: Delay of Treatment 2 joining the platform after 0 patients (100% of control patients), 30, 50 and 70 patients in the control arm already on the trial. Column grids: Ad‐hoc analysis *t* = 1 is triggered by Treatment 1 against the shared control at information fraction 0.4, 0.6, 0.8 and 1 (primary analysis). Cases of equal conditional Type I error rates are omitted.
**Figure S19:** L4: Estimated Hazard Ratio and Observed Pooled events. Smoothed tile graph comparing the estimated conditional Type I error rate at the ad‐hoc analysis *t* = 1 and the primary analysis *t* = 2 given the observed number of events and estimated log hazard ratio. Tiles represent the original tiles. Dots are overlayed showing the smoothed tiles using the smoothed decision indicator. Ad‐hoc analysis *t* = 1 is triggered by Treatment 1 against the shared control at information fraction 0.4, 0.6, 0.8 and 1 (primary analysis). Cases of equal conditional Type I error rates are omitted.
**Figure S20:** L1: Observed events per Arm. Smoothed tile graph comparing the estimated conditional Type I error rate at the ad‐hoc analysis *t* = 1 and the primary analysis *t* = 2 given the observed number of events per arm. Tiles represent the smoothed tiles. Dots are overlayed showing the LDA predictions. Row grids: Delay of Treatment 2 joining the platform after 0 patients (100% of control patients), 30, 50 and 70 patients in the control arm already on the trial. Column grids: Ad‐hoc analysis *t* = 1 is triggered by Treatment 1 against the shared control at information fraction 0.4, 0.6, 0.8 and 1 (primary analysis). Cases of equal conditional Type I error rates are omitted.
**Figure S21:** L3: Estimated events per Arm. Smoothed tile graph comparing the estimated conditional Type I error rate at the ad‐hoc analysis *t* = 1 and the primary analysis *t* = 2 given the estimated number of events per arm. Tiles represent the smoothed tiles. Dots are overlayed showing the LDA predictions. Row grids: Delay of Treatment 2 joining the platform after 0 patients (100% of control patients), 30, 50 and 70 patients in the control arm already on the trial. Column grids: Ad‐hoc analysis *t* = 1 is triggered by Treatment 1 against the shared control at information fraction 0.4, 0.6, 0.8 and 1 (primary analysis). Cases of equal conditional Type I error rates are omitted.
**Figure S22:** L4: Estimated Hazard Ratio and Observed Pooled events. Smoothed tile graph comparing the estimated conditional Type I error rate at the ad‐hoc analysis *t* = 1 and the primary analysis *t* = 2 given the observed number of events and estimated log hazard ratio. Tiles represent the smoothed tiles. Dots are overlayed showing the LDA predictions. Ad‐hoc analysis *t* = 1 is triggered by Treatment 1 against the shared control at information fraction 0.4, 0.6, 0.8 and 1 (primary analysis). Cases of equal conditional Type I error rates are omitted.
**Figure S23:** L2: Observed Hazard Ratio and Observed Pooled events. Tile graph comparing the estimated conditional Type I error rate at the ad‐hoc analysis *t* = 1 and the primary analysis *t* = 2 given the observed number of events and observed log hazard ratio. Dots are overlayed showing the condition ($-\sqrt{d_{0_{\left[2\right]}}\left(1\right)+d_{2}\left(1\right)}\times 0.5\times \log{\left(\hat{\theta_{2}\left(1\right)}\right)}^{2}>\chi_{1,1-5\%}^{2}$ and $\hat{\theta_{2}\left(1\right)}<1$). Ad‐hoc analysis *t* = 1 is triggered by Treatment 1 against the shared control at information fraction 0.4, 0.6, 0.8 and 1 (primary analysis). Cases of equal conditional Type I error rates are omitted.
**Figure S24:** Estimated maximum Type I error rate for the comparison of Treatment 2 and the control using the approximate adaptation rule (Equation (10)) of L2 with the categorisation of the HR according to quantiles or alternatively when results of Treatment 1 against the shared control at an interim analysis (information fraction [IF] 0.4, 0.6, 0.8), or at the primary analysis (information fraction 1) (see grids) of Treatment 1 is published, and delayed entry of Treatment 2 (*x*‐axis). Entry at 0 denotes an immediate start of Treatment 2 (i.e., all arms start at the same time), an entry at 70 denotes a late entry. An additional simulation was performed using the simplified adaptation rule using the log‐rank test statistic (triangle).
**Figure S25:** Best case estimated power when analysis of Treatment 1 against the shared control is published at information fraction 0.4, 0.6, 0.8 and 1 (primary analysis) (see grids) from Treatment 1, and delayed entry of Treatment 2 (*x*‐axis). Entry at 0 denotes an immediate start of Treatment 2, an entry at 70 denotes a late entry. Treatment 1 under null (HR 1).
**Figure S26:** Mean number of events in the control or Treatment 2, as well as pooled (control and Treatment 2). Mean number of patients recruited to Treatment 2 at the time of analysis of Treatment 1.
**Figure S27:** Frequency of primary analysis of Treatment 2 after an interim/primary analysis of Treatment 1 (i.e., a possibility for leakage).
**Table S1:** Input parameters for the main simulation.

**Data S1:** Supporting information.

## Data Availability

R‐Code for the simulation is provided in the Supporting Information.
