# Supplementary material for: Impact of Information Leakage in Platform Trials With Survival Endpoints on Type I Error Control
Source: Pharm Stat. 2026 Jun 25;25(4):e70106. doi: 10.1002/pst.70106 (PMC13305155; doi:10.1002/pst.70106)
Supplement: Supplementary file 1 — Figure S1: Top: Estimated maximum Type I error rate for the comparison of Treatment 2 and the control using the approximate adaptation rule when results of Treatment 1 against the shared control at an interim analysis (information fraction [IF] 0.4, 0.6, 0.8), or at the primary analysis (information fraction 1) (see grids) of Treatment 1 is published, and delayed entry of Treatment 2 (x‐axis). Entry at 0 denotes an immediate start of Treatment 2 (i.e., all arms start at the same time), an entry at 70 denotes a late entry. Bottom: Mean number of events in the control or Treatment 2, as well as pooled (control and Treatment 2). Mean number of patients recruited to Treatment 2 at the time of analysis of Treatment 1. Figure S2: Frequency (%) that the pre‐planned analysis of Treatment 2 will take place after the first analysis of Treatment 1 (at information fraction [IF] 0.4, 0.6, 0.8.1). Figure S3: Top: Estimated maximum Type I error rate for the comparison of Treatment 2 and the control using the approximate adaptation rule when results of Treatment 1 against the shared control at an interim analysis (information fraction [IF] 0.4, 0.6, 0.8), or at the primary analysis (information fraction 1) (see grids) of Treatment 1 is published, and delayed entry of Treatment 2 (x‐axis). Entry at 0 denotes an immediate start of Treatment 2 (i.e., all arms start at the same time), an entry at 70 denotes a late entry. Bottom: Mean number of events in the control or Treatment 2, as well as pooled (control and Treatment 2). Mean number of patients recruited to Treatment 2 at the time of analysis of Treatment 1. Treatment 1 with moderate effect (HR 0.833). Figure S4: Top: Estimated maximum Type I error rate for the comparison of Treatment 2 and the control using the approximate adaptation rule when results of Treatment 1 against the shared control at an interim analysis (information fraction [IF] 0.4, 0.6, 0.8), or at the primary analysis (information fraction 1) (see grids) of Treatme [file PST-25-0-s002.pdf]

Supplement - Impact of information leakage in platform trials with survival endpoints on type I error control

Quynh Nguyen, Martin Posch, Benjamin Hofner, Franz König

April 2026

Contents

A Estimated maximum type I error rate under varying assumptions **2**
- A.1 Varying treatment effect of treatment 1 . . . 5
  - A.1.1 Moderate effect (HR = 0.833) . . . 5
  - A.1.2 High effect (HR = 0.625) . . . 5
  - A.1.3 Comparison of the estimated maximum type I error rate varying treatment effect of treatment 1 . . . 7
- A.2 Varying median survival under global null . . . 10
- A.3 Varying underlying distribution . . . 13
  - A.3.1 Multi-state (MS) model . . . 13
  - A.3.2 Delayed treatment effect . . . 17

B Approach to define simplified adaptation rule and evaluation of the potential bias of the maximum type I error rate **18**
- B.1 Simplified adaptation Rule for $L1$, $L3$ and $L4$ . . . 18
  - B.1.1 Smoothing algorithm . . . 18
  - B.1.2 Linear discriminant analysis . . . 19
- B.2 Simplified adaptation Rule for $L2$ . . . 19
- B.3 Tile graphs showing conditional error rates . . . 19
- B.4 Smoothed tile graphs . . . 26
- B.5 LDA overlay on tile graphs . . . 30
- B.6 Overlay of approximation of log-rank test statistic . . . 34
- B.7 Impact of the categorisation of the HR on the maximum type I error rate for $L2$ . . . 37

C Power **38**

D Main simulation input parameter **40**

A Estimated maximum type I error rate under varying assumptions

For completeness and comparability, we repeat Figure 3 from the main publication here again in Figure 1. The underlying assumptions for Figure 3 in the main publication were that no treatment had an effect (e.g., global null, HR: 1), and for each arm a median survival of 5 months was assumed. In additional simulations, we varied

- the effect of treatment 1, i.e. varying the hazard ratio for treatment 1 from 0.5 to 1.2, while keeping treatment 2 under the null, and
- the effect of the assumed median survival under the null, i.e. varying the median (and therefore the shape of the exponential function) for all arms from 3 to 13.88 months.

We will show the estimated maximum type I error rates for the comparison of treatment 2 and the control using the approximate adaptation rule for selected hazard ratios of treatment 1 (Figure 3 and Figure 4) and a comparison of the estimated maximum type I error rate for all hazard ratios of treatment 1 (Figure 5). For varying median survival, we only show the comparison of the estimated maximum type I error rate for all median survivals (Figure 7) as similar patterns are seen in the single graphs. Throughout the document we will consider the marginal type I error rate for the comparison of treatment 2 and the control only.

The additional simulations will underline the findings of the main paper: The amount of inflation is dependent on the type of information and how well the log-rank test statistic can be predicted, and the time point of information release as this affects the variability for recalculation. Here we have two opposing effects. On the one hand, it is good to have as much information from treatment 2 as possible in order to estimate the treatment effect and log-rank test statistic as precisely as possible. But on the other hand, the two analysis points should be far enough apart, as the rejection regions from the two analysis time points become more divergent and thus lead to a higher inflation of the type 1 error rate.

Figure 2 displays the frequency of a primary analysis of treatment 2 after an analysis of treatment 1, i.e., the frequency of a possible leakage. When both treatments start recruitment at the same time (i.e., delay of 0), then treatment 2 will only face a possible leakage in 50 % of the simulations. In the other half of simulations, treatment 2 already reached the primary analysis before treatment 1 and thus, no leakage is possible in these cases.

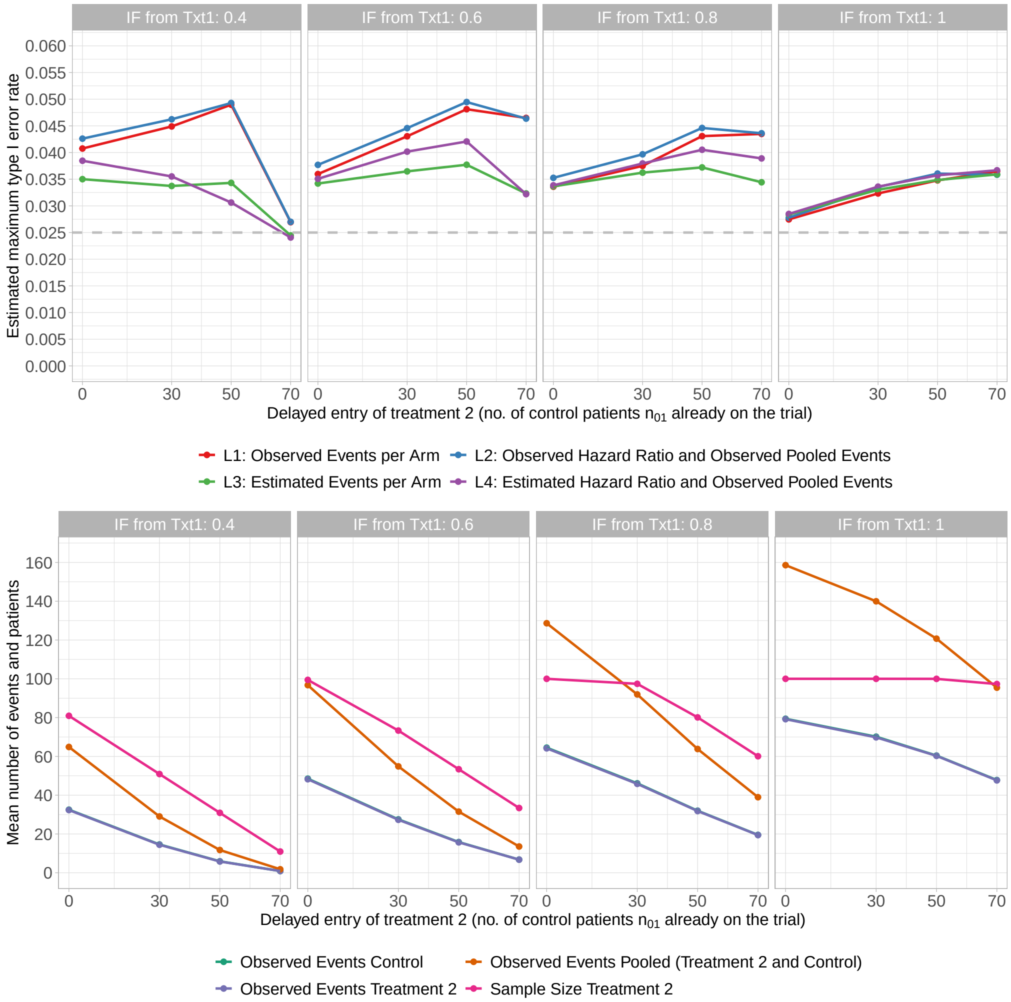

Supplementary Figure 1: Top: Estimated maximum type I error rate for the comparison of treatment 2 and the control using the approximate adaptation rule when results of treatment 1 against the shared control at an interim analysis (information fraction (IF) 0.4, 0.6, 0.8), or at the primary analysis (information fraction 1) (see grids) of treatment 1 is published, and delayed entry of treatment 2 (x-axis). Entry at 0 denotes an immediate start of treatment 2 (i.e. all arms start at the same time), an entry at 70 denotes a late entry. Bottom: Mean number of events in the control or treatment 2, as well as pooled (control and treatment 2). Mean number of patients recruited to treatment 2 at the time of analysis of treatment 1.

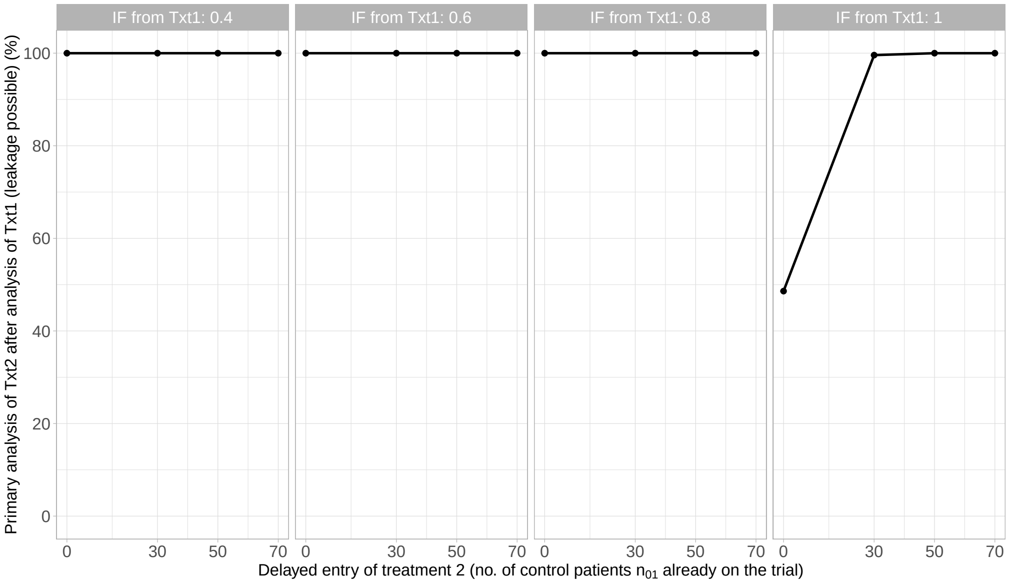

Supplementary Figure 2: Frequency (%) that the pre-planned analysis of treatment 2 will take place after the first analysis of treatment 1 (at information fraction (IF) 0.4, 0.6, 0.8. 1).

A.1 Varying treatment effect of treatment 1

A.1.1 Moderate effect (HR = 0.833)

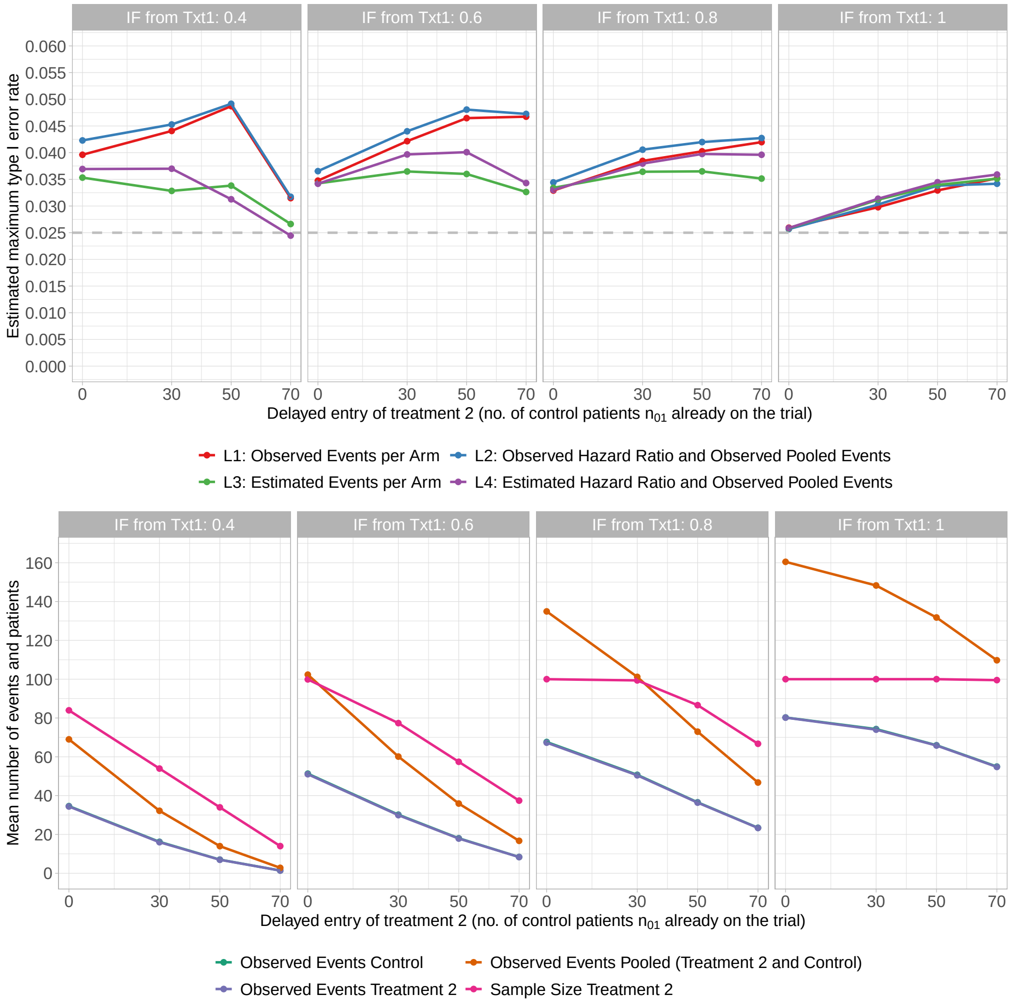

Supplementary Figure 3: Top: Estimated maximum type I error rate for the comparison of treatment 2 and the control using the approximate adaptation rule when results of treatment 1 against the shared control at an interim analysis (information fraction (IF) 0.4, 0.6, 0.8), or at the primary analysis (information fraction 1) (see grids) of treatment 1 is published, and delayed entry of treatment 2 (x-axis). Entry at 0 denotes an immediate start of treatment 2 (i.e. all arms start at the same time), an entry at 70 denotes a late entry. Bottom: Mean number of events in the control or treatment 2, as well as pooled (control and treatment 2). Mean number of patients recruited to treatment 2 at the time of analysis of treatment 1. Treatment 1 with moderate effect (HR 0.833).

A.1.2 High effect (HR = 0.625)

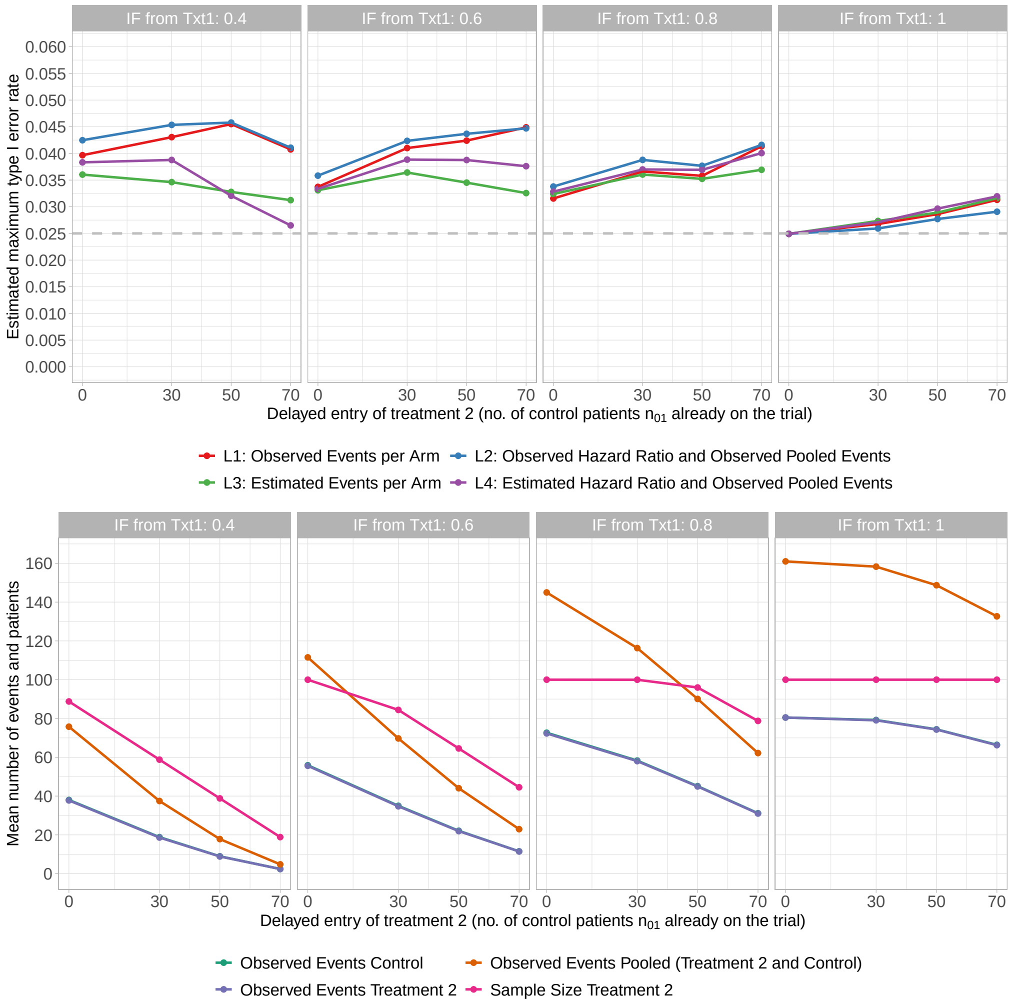

Supplementary Figure 4: Top: Estimated maximum type I error rate for the comparison of treatment 2 and the control using the approximate adaptation rule when results of treatment 1 against the shared control at an interim analysis (information fraction (IF) 0.4, 0.6, 0.8), or at the primary analysis (information fraction 1) (see grids) of treatment 1 is published, and delayed entry of treatment 2 (x-axis). Entry at 0 denotes an immediate start of treatment 2 (i.e. all arms start at the same time), an entry at 70 denotes a late entry. Bottom: Mean number of events in the control or treatment 2, as well as pooled (control and treatment 2). Mean number of patients recruited to treatment 2 at the time of analysis of treatment 1. Treatment 1 with high effect (HR 0.625).

A.1.3 Comparison of the estimated maximum type I error rate varying treatment effect of treatment 1

For varying treatment effects of treatment 1 (i.e. hazard ratio from 0.5 to 1.2) the estimated maximum type I error rate (Figure 5 top panel), the mean number of events and sample size of treatment 2 (Figure 5 bottom panel), and the frequency of a primary analysis of treatment 2 after an analysis of treatment 1 (Figure 6) are displayed in relation to the delayed entry of treatment 2. These figures combine the previous single graphs (e.g. 1, 3 and 4) into one overall graph for a better comparison.

A low hazard ratio for treatment 1 (i.e. treatment 1 is effective) leads to longer time until the required events for an interim or primary analysis of treatment 1 is observed. Thus, in comparison to the global null, treatment 2 has more time to recruit patients and accumulate more events. Hence, the amount of information once an interim analysis or the primary analysis of treatment 1 is performed, is increased (Figure 5 bottom panel). Furthermore, treatment 2 reaches its primary analysis more frequently than treatment 1 if treatment 1 is very effective (i.e. hazard ratio of 0.5 to 0.833) when both arms start at the same time (Figure 6 far right). In these cases, no leakage is possible. As under the global null, the estimated maximum type I error rate is usually highest when the observed hazard ratio for treatment 2 and the pooled number of events are leaked ($L2$) as this leakage provides the best estimate of the log-rank test statistic. A good estimate of the treatment effect or log-rank test statistic using the leaked information ($L1$ to $L4$) increases the maximum type I error. However, on the other hand lower hazard ratios of treatment 1 lead to more patients recruited and events accumulated at time $t = 1$ for treatment 2 which moves the analysis $t = 1$ closer to the planned primary analysis at time $t = 2$ and reduces the additional rejections at time $t = 1$. Therefore, lower maximum type I error rates are observed with lower hazard ratios of treatment 1 in comparison to higher hazard ratios of treatment 1 (see e.g. top left grid in Figure 5 when information is leaked at 40% IF from treatment 1). As under the global null, in some cases treatment 2 joins the study very late while the analysis at time $t = 1$ is performed early, resulting in no leakage for treatment 2 and thus reducing the maximum type I error rate close to the significance level. Similarly, in some cases, treatment 2 already reached the primary analysis before the primary analysis of treatment 1, thus no leakage is possible and a lower maximum type I error rate is observed (see e.g., top right grid in Figure 5 when treatment 1 has a low hazard ratio of 0.833 or lower and treatment 2 joins the trial early).

Overall, the varying treatment effect of treatment 1 mostly affects the amount of information that is accumulated for treatment 2. If treatment 1 is effective (e.g. lower hazard ratio), it takes longer until the required events for an interim or primary analysis of treatment 1 is reached. In this time, more patients can be recruited to treatment 2 and also more events are observed. With more information, the overlap from the ad-hoc analysis at time $t = 1$ and the planned primary analysis decreases and thus reduces the number of additional rejections at time $t = 1$ which would have not been rejected at the primary analysis. This reduces the maximum type I error rate.

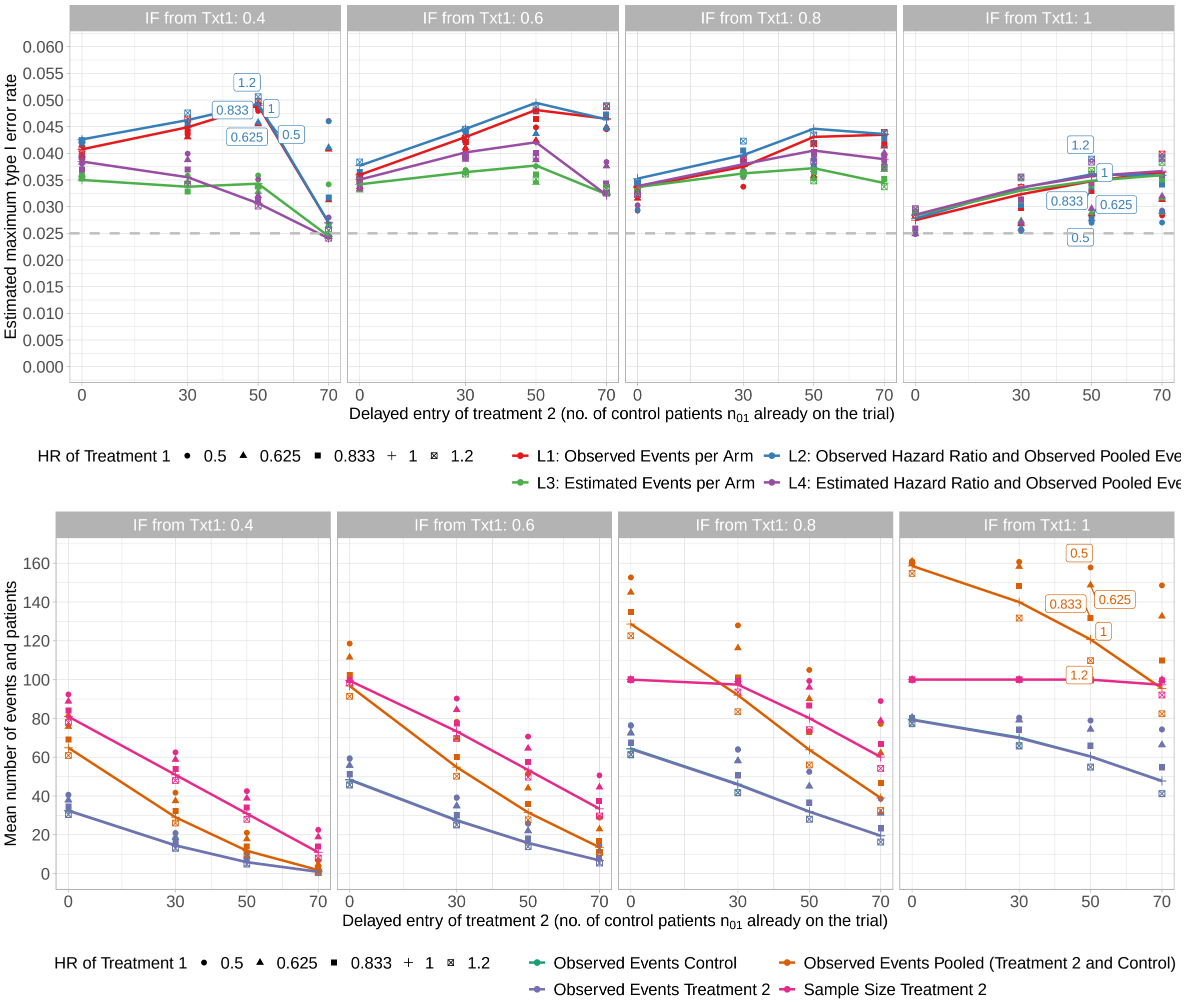

Supplementary Figure 5: Top: Estimated maximum type I error rate for the comparison of treatment 2 and the control using the approximate adaptation rule for treatment 2 when results of treatment 1 against the shared control at an interim analysis (information fraction (IF) 0.4, 0.6, 0.8), or at the primary analysis (information fraction 1) (see grids) of treatment 1 is published, and delayed entry of treatment 2 (x-axis). Entry at 0 denotes an immediate start of treatment 2 (i.e. all arms start at the same time), an entry at 70 denotes a late entry. Varying treatment effects of treatment 1 are displayed in different shapes. For the sake of simplicity and convenience, the different hazard ratios for treatment 1 are additionally labelled for selected results in selected grids only. The results for the global null as in the main paper are displayed with connecting lines. Bottom: Mean number of events in the control or treatment 2, as well as pooled (control and treatment 2). Mean number of patients recruited to treatment 2 at the time of analysis of treatment 1. Varying treatment effects of treatment 1 are displayed in different shapes. For the sake of simplicity and convenience, the different hazard ratios for treatment 1 are additionally labelled for selected results in selected grids only. The results for the global null as in the main paper are displayed with connecting lines.

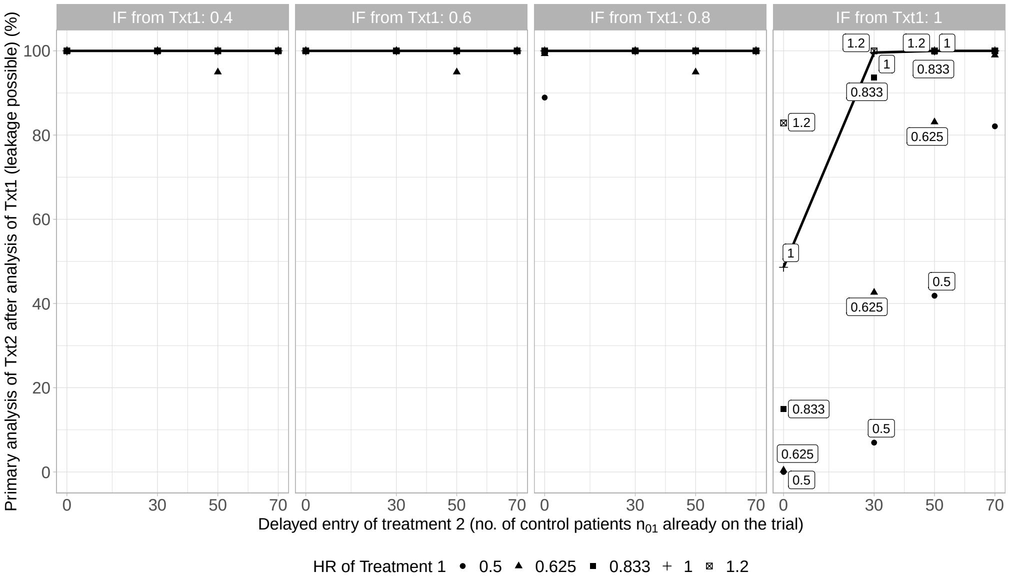

Supplementary Figure 6: Frequency (%) that the pre-planned analysis of treatment 2 will take place after the first analysis of treatment 1 (at information fraction (IF) 0.4, 0.6, 0.8. 1). Varying treatment effects of treatment 1 are displayed by different symbols. For the sake of simplicity and convenience, the different hazard ratios for treatment 1 are additionally labelled for selected results in selected grids only. The results for the global null as in the main paper are displayed with connecting lines.

A.2 Varying median survival under global null

As the pattern for the estimated maximum type I error rate is similar when varying the median survival, we refrain from showing the individual graphs for each median survival but directly show the comparison between all median survival times. For varying median survival times for all treatments under the global null (i.e., median of 3, 5, 8 and 13.88) the estimated maximum type I error rate (Figure 7 top panel), the mean number of events and sample size of treatment 2 (Figure 7 bottom panel), and the frequency of a primary analysis of treatment 2 after an analysis of treatment 1 (Figure 8) are displayed in relation to the delayed entry of treatment 2.

An overall high median survival leads to longer time until the required events for an interim or primary analysis of treatment 1 is observed. Thus, in comparison to the global null, treatment 2 has more time to recruit patients and accumulate more events. Hence, the amount of information increases once an interim analysis or the primary analysis of treatment 1 is performed (Figure 7 bottom panel). As under the global null, the maximum type I error rate is highest when the observed hazard ratio for treatment 2 and the pooled number of events are leaked ($L2$) as this leakage provides the best estimate of the log-rank test statistic. A good estimate of the treatment effect or log-rank test statistic using the leaked information ($L1$ to $L4$) increases the maximum type I error. However, on the other hand with more information at time $t = 1$ for treatment 2 moves the analysis $t = 1$ closer to the planned primary analysis at time $t = 2$ and reduces the additional rejections at time $t = 1$. Therefore, a lower maximum type I error rate is observed with low median survival times in comparison to higher median survivals (see e.g. top left grid in Figure 7 when information is leaked at 40% IF from treatment 1). As under the global null, in some cases treatment 2 joins the study very late while the analysis at time $t = 1$ is performed early, resulting in no leakage for treatment 2 and thus reducing the maximum type I error rate close to the significance level. Similarly, in some cases, treatment 2 already reached the primary analysis before the primary analysis of treatment 1, thus no leakage is possible and a lower maximum type I error rate is observed (see e.g. top right grid in Figure 7 when a median survival of 13.88 is assumed).

Overall, the varying effect of the median survival times under the global null affects the amount of information that is accumulated for treatment 2. If the underlying median survival is high, it takes longer until the required events for an interim or primary analysis of treatment 1 is reached. In this time, more patients can be recruited to treatment 2 and also more events are observed. With more information, the overlap from the ad-hoc analysis at time $t = 1$ and the planned primary analysis decreases and thus reduces the number of additional rejections at time $t = 1$ which would have not been rejected at the primary analysis. This reduces the estimated maximum type I error rate.

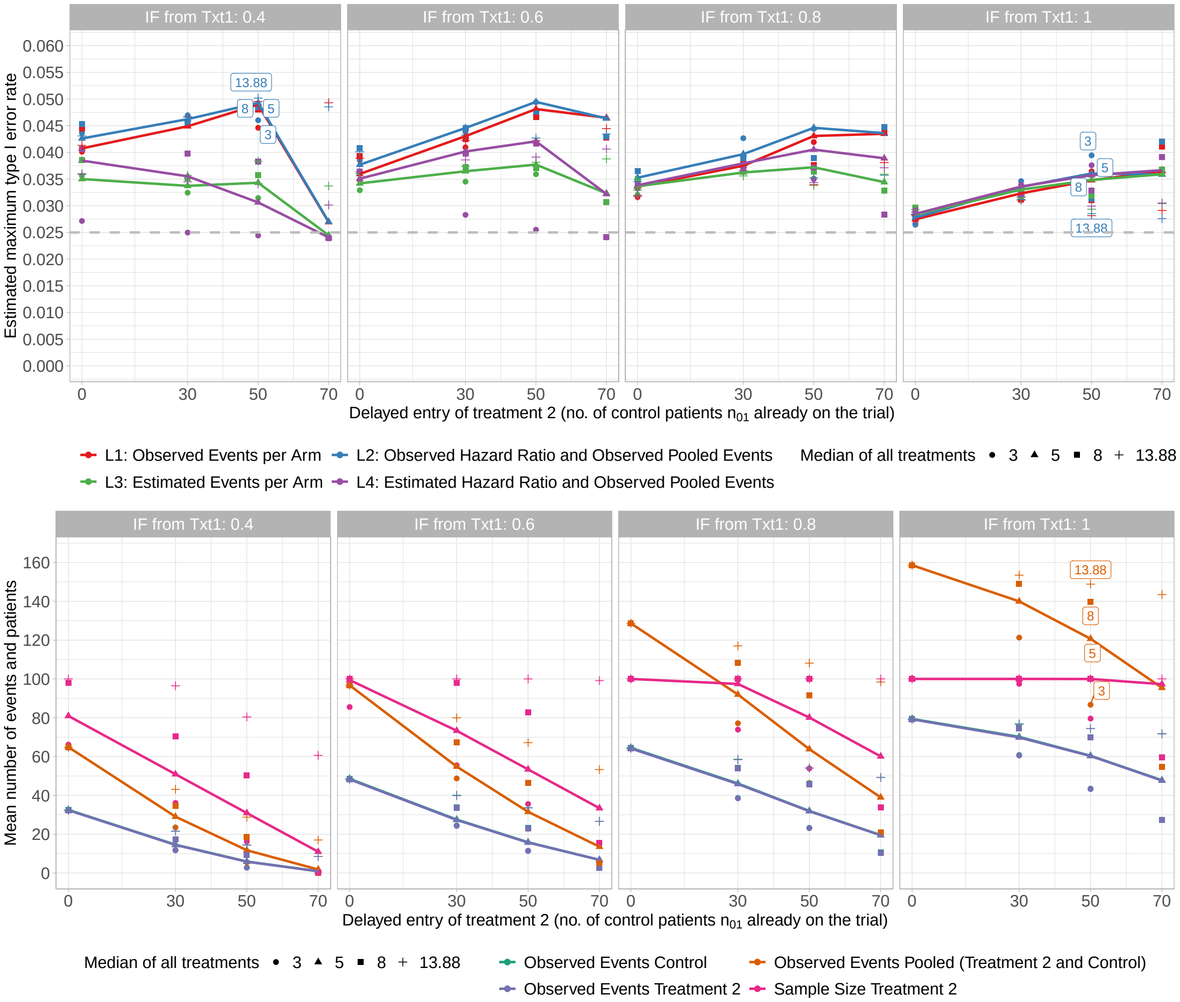

Supplementary Figure 7: Top: Estimated maximum type I error rate for the comparison of treatment 2 and the control using the approximate adaptation rule for treatment 2 when results of treatment 1 against the shared control at an interim analysis (information fraction (IF) 0.4, 0.6, 0.8), or at the primary analysis (information fraction 1) (see grids) of treatment 1 is published, and delayed entry of treatment 2 (x-axis). Entry at 0 denotes an immediate start of treatment 2 (i.e. all arms start at the same time), an entry at 70 denotes a late entry. Varying median survival for all treatments under the global null are displayed in different shapes. For the sake of simplicity and convenience, the different medians are additionally labelled for selected results in selected grids. The results for the global null as in the main paper are displayed with connecting lines. Bottom: Mean number of events in the control or treatment 2, as well as pooled (control and treatment 2). Mean number of patients recruited to treatment 2 at the time of analysis of treatment 1. Varying median survival for all treatments under the global null are displayed in different shapes. For the sake of simplicity and convenience, the different medians are additionally labelled for selected results in selected grids. The results for the global null as in the main paper are displayed with connecting lines.

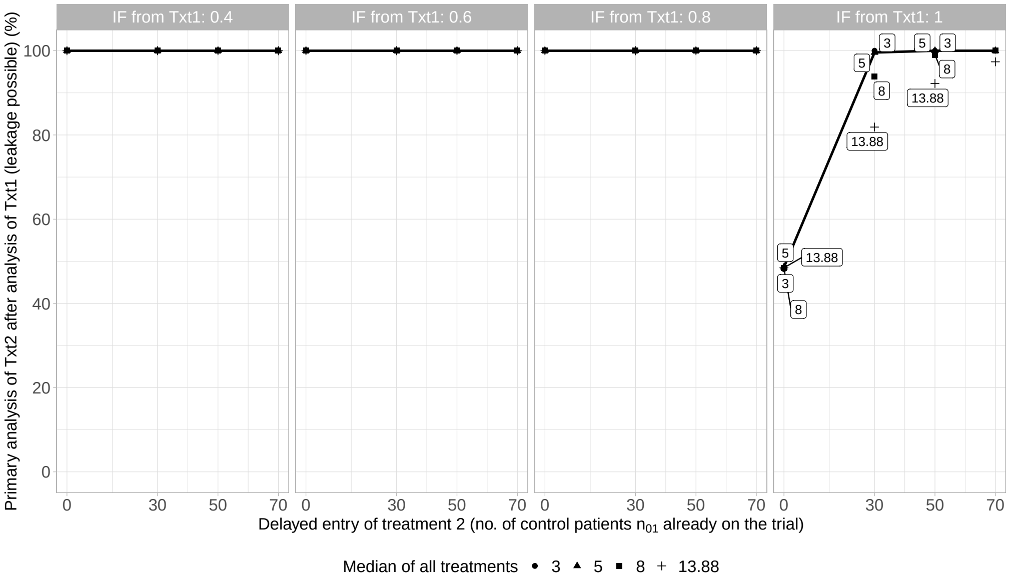

Supplementary Figure 8: Frequency that the pre-planned analysis of treatment 2 will take place after the first analysis of treatment 1 (at information fraction (IF) 0.4, 0.6, 0.8. 1). Varying median survival for all treatments under the global null are displayed in different colours for a delayed entry of treatment 2 after 50 patients in the control arm already recruited. For the sake of simplicity and convenience, the different medians are additionally labelled for selected results in selected grids. The results for the global null as in the main paper are displayed with connecting lines.

A.3 Varying underlying distribution

Following the approach described by Ristl et al. (2021) we considered a multi-state distribution and a delayed treatment effect. For the exponential distribution, we did not specify the exact type of event for the time-to-event analyses. For progression-free survival, the events would be progressive disease (PD) or death. For overall survival, the event would be death. Following the approach by Ristl et al. (2021), we consider progression-free survival as an example to evaluate the effect of a different distribution for the data generation other than the exponential distribution. The multi-state (MS) model can be split into three states: no progression or death, progression, and death. For each of these states a hazard can be defined, i.e., a distinct hazard from no progression or death to death, a hazard from no progression or death to progression, and a hazard from progression to death.

A.3.1 Multi-state (MS) model

For the MS distribution we assumed a hazard for death before PD corresponding to a median survival time of 6 months, a median for death after PD of 4 months and a median for PD of 3 months. The median times were chosen to approximate an overall median of 5 months as assumed for the exponential distribution.

Three scenarios using a MS distribution were evaluated: 1. all arms follow a MS distribution (see Figure 9), 2. only treatment 1 follows a MS distribution but treatment 2 and the control follow an exponential distribution (see Figure 10), 3. treatment 1 follows an exponential distribution but treatment 2 and the control follow a MS distribution (see Figure 11). In all three cases, the null hypothesis between treatment 2 and the control is true.

In all three scenarios, a similar trend in inflation is observed in comparison to the original data generation using the exponential distribution for all arms. The deviation of the maximum type I error rate may be small due to the chosen assumptions for the medians using the MS distribution which were chosen to approximate the same median time as used for the exponential distribution.

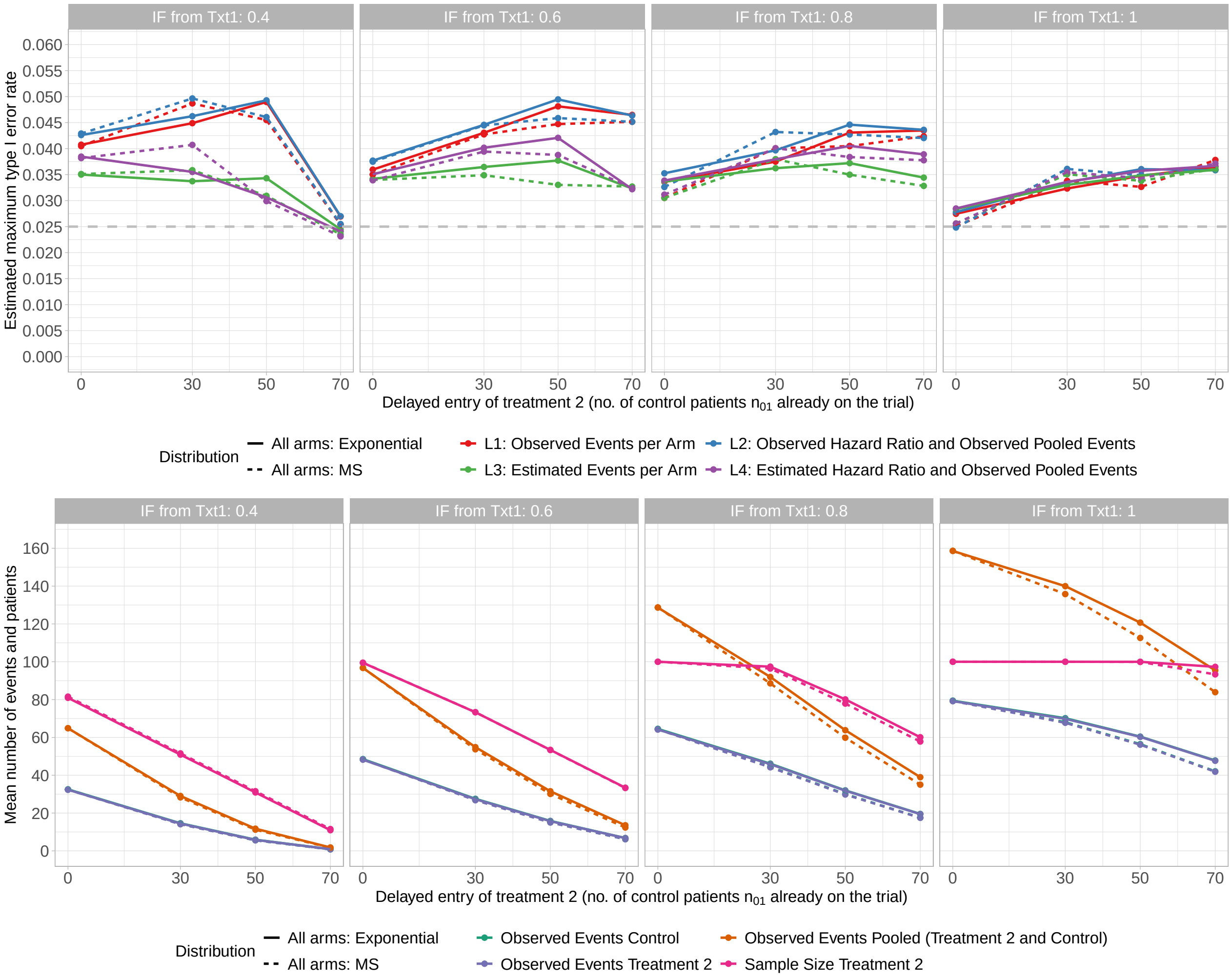

Supplementary Figure 9: Top: Estimated maximum type I error rate for the comparison of treatment 2 and the control using the approximate adaptation rule when results of treatment 1 against the shared control at an interim analysis (information fraction (IF) 0.4, 0.6, 0.8), or at the primary analysis (information fraction 1) (see grids) of treatment 1 is published, and delayed entry of treatment 2 (x-axis). Entry at 0 denotes an immediate start of treatment 2 (i.e. all arms start at the same time), an entry at 70 denotes a late entry. Bottom: Mean number of events in the control or treatment 2, as well as pooled (control and treatment 2). Mean number of patients recruited to treatment 2 at the time of analysis of treatment 1. Data generation for all arms following an exponential distribution (solid lines), or following an multi-state (MS) distribution (dotted lines).

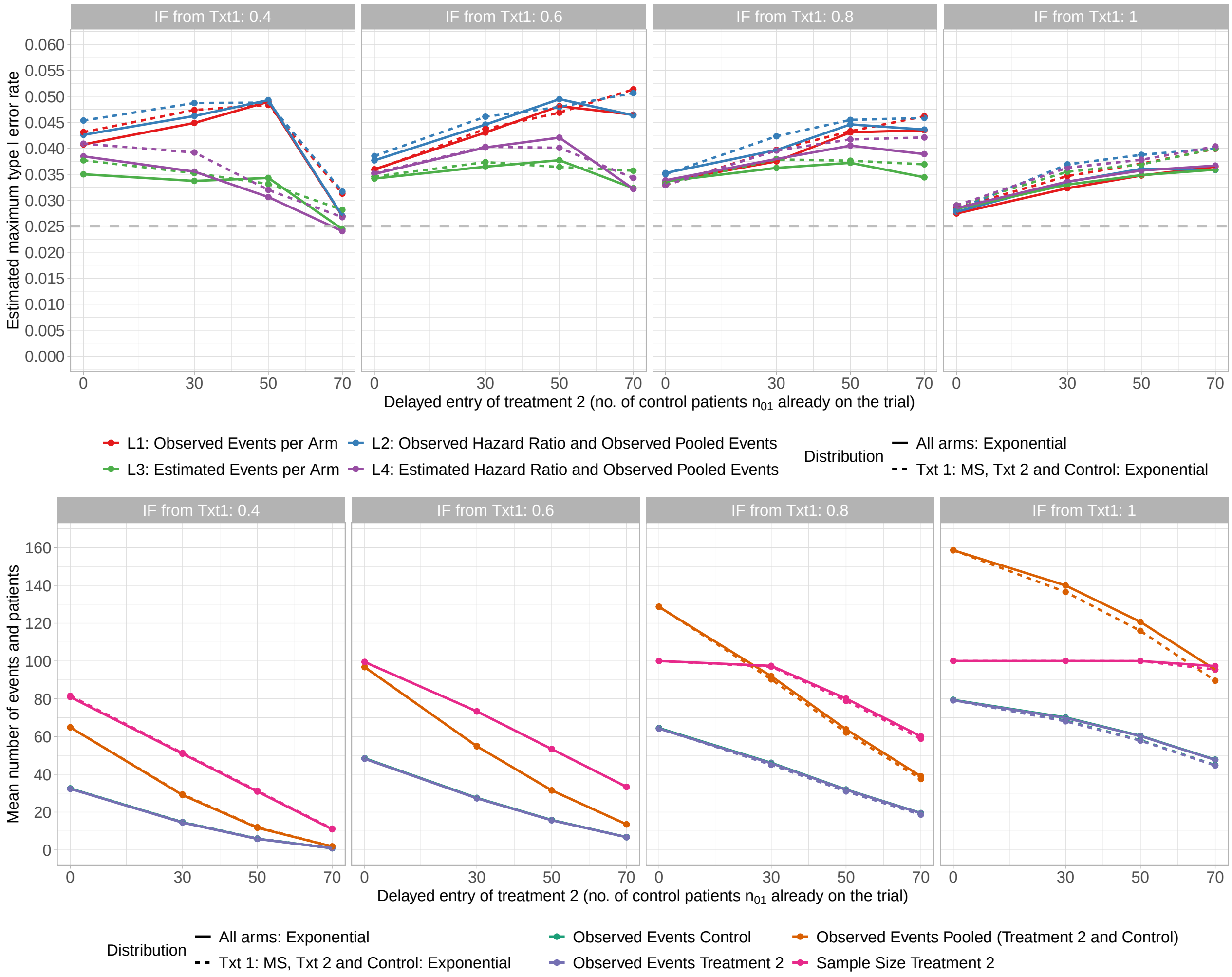

Supplementary Figure 10: Top: Estimated maximum type I error rate for the comparison of treatment 2 and the control using the approximate adaptation rule when results of treatment 1 against the shared control at an interim analysis (information fraction (IF) 0.4, 0.6, 0.8), or at the primary analysis (information fraction 1) (see grids) of treatment 1 is published, and delayed entry of treatment 2 (x-axis). Entry at 0 denotes an immediate start of treatment 2 (i.e. all arms start at the same time), an entry at 70 denotes a late entry. Bottom: Mean number of events in the control or treatment 2, as well as pooled (control and treatment 2). Mean number of patients recruited to treatment 2 at the time of analysis of treatment 1. Data generation for all arms following an exponential distribution (solid lines), or or mixture where treatment 1 follows an multi-state (MS) distribution but treatment 2 and the control follow an exponential distribution (dotted lines).

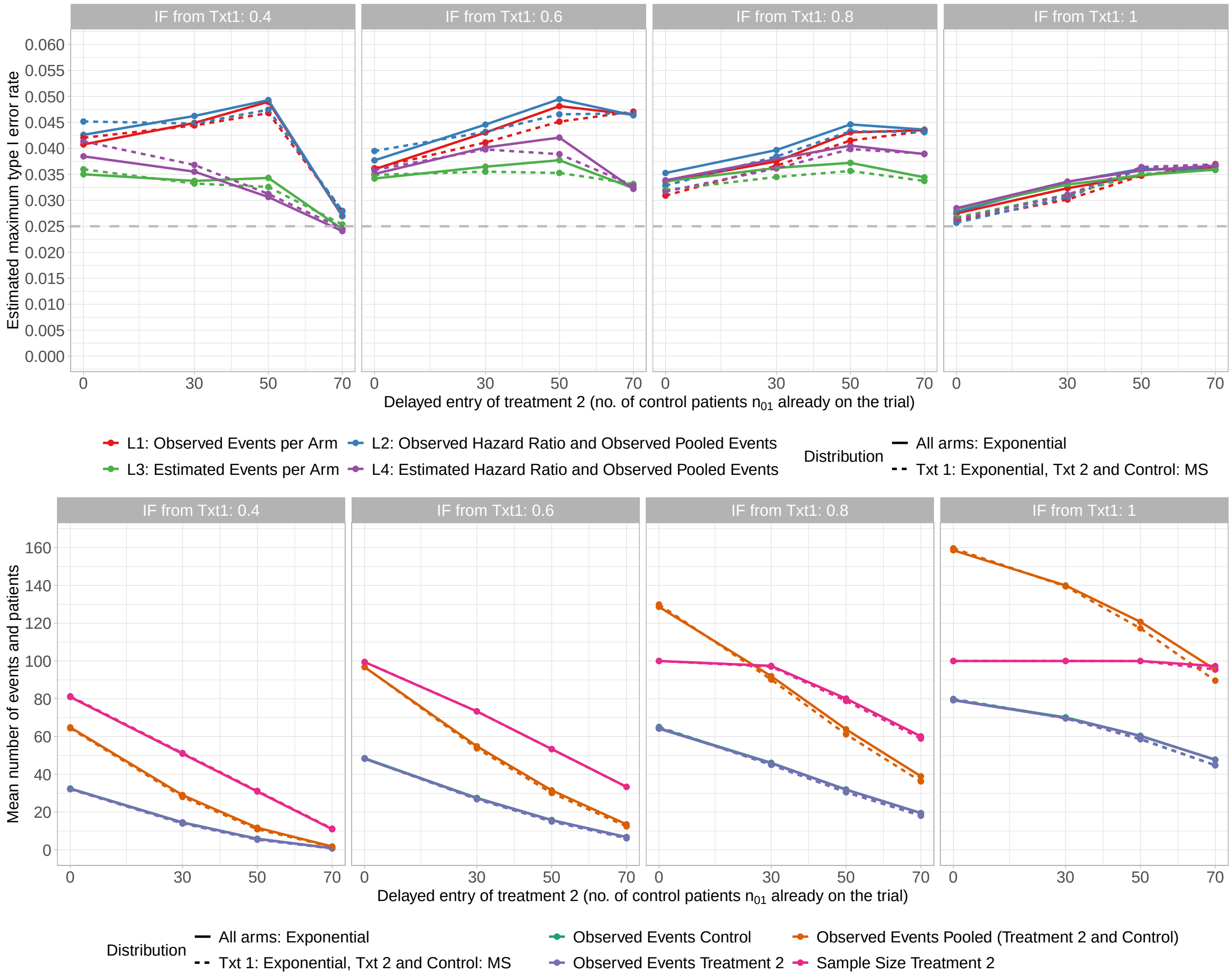

Supplementary Figure 11: Top: Estimated maximum type I error rate for the comparison of treatment 2 and the control using the approximate adaptation rule when results of treatment 1 against the shared control at an interim analysis (information fraction (IF) 0.4, 0.6, 0.8), or at the primary analysis (information fraction 1) (see grids) of treatment 1 is published, and delayed entry of treatment 2 (x-axis). Entry at 0 denotes an immediate start of treatment 2 (i.e. all arms start at the same time), an entry at 70 denotes a late entry. Bottom: Mean number of events in the control or treatment 2, as well as pooled (control and treatment 2). Mean number of patients recruited to treatment 2 at the time of analysis of treatment 1. Data generation for all arms following an exponential distribution (solid lines), or or mixture where treatment 1 follows an exponential distribution but treatment 2 and the control follow a multi-state (MS) distribution (dotted lines).

A.3.2 Delayed treatment effect

For the delayed treatment effect, we assumed that in the first 3 months, the median for the three states (death before PD, death after PD, PD) are 5 months, 3 months and 3 months. Afterwards, the medians are 8 months, 6 months and 5 months. The median times were chosen to approximate an overall median of 5 months as assumed for the exponential distribution.

One scenario using a delayed treatment effect was evaluated: all arms follow a delayed treatment effect (see Figure 12) and the null hypothesis between treatment 2 and the control is true.

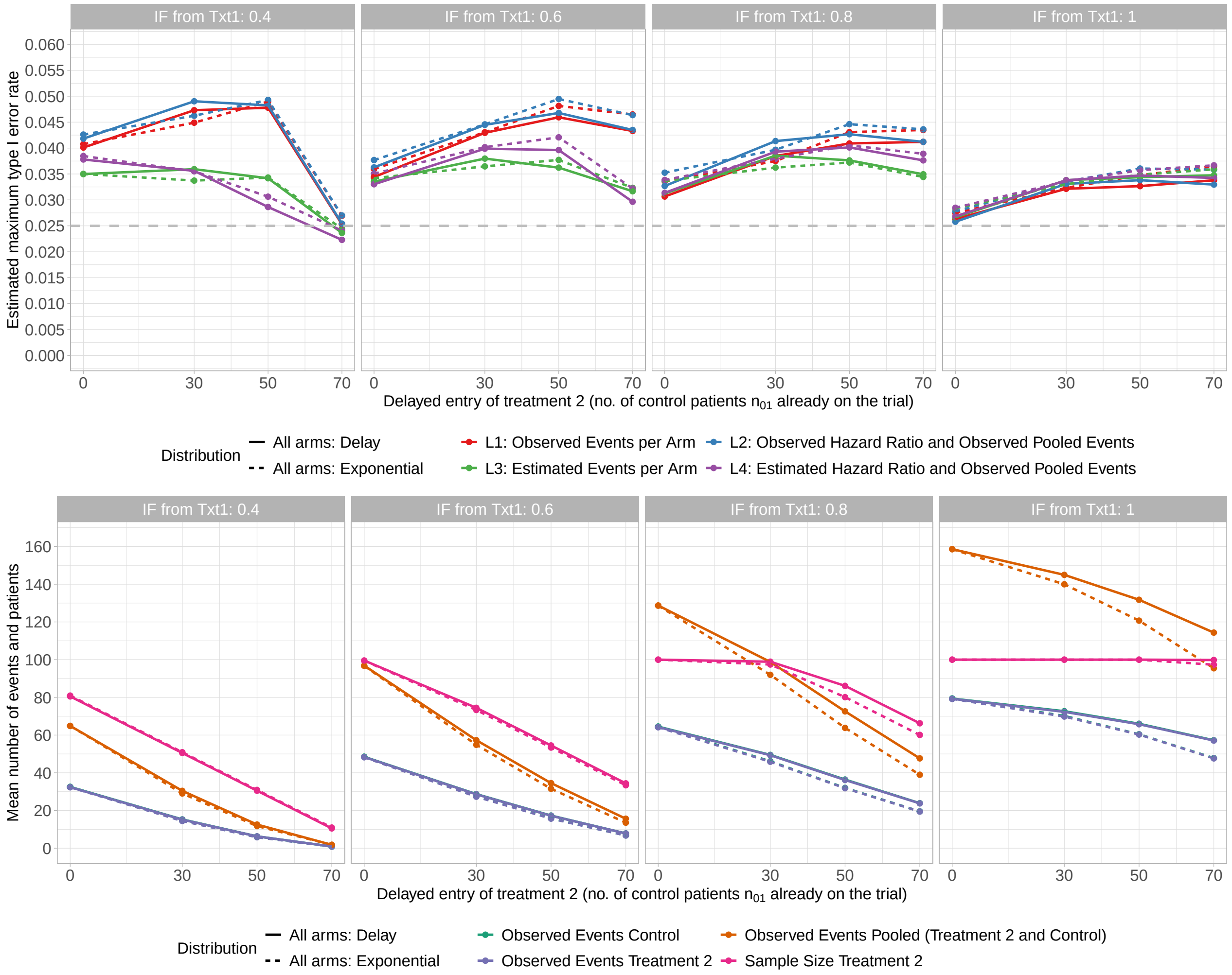

Supplementary Figure 12: Top: Estimated maximum type I error rate for the comparison of treatment 2 and the control using the approximate adaptation rule when results of treatment 1 against the shared control at an interim analysis (information fraction (IF) 0.4, 0.6, 0.8), or at the primary analysis (information fraction 1) (see grids) of treatment 1 is published, and delayed entry of treatment 2 (x-axis). Entry at 0 denotes an immediate start of treatment 2 (i.e. all arms start at the same time), an entry at 70 denotes a late entry. Bottom: Mean number of events in the control or treatment 2, as well as pooled (control and treatment 2). Mean number of patients recruited to treatment 2 at the time of analysis of treatment 1. Data generation for all arms following an exponential distribution (solid lines), or following an delayed distribution (dotted lines).

A similar trend in inflation is observed in comparison to the original data generation using the exponential distribution for all arms. The deviation of the maximum type I error rate may be small due to the chosen assumptions for the medians using the delayed distribution which were chosen to approximate the same median time as used for the exponential distribution.

B Approach to define simplified adaptation rule and evaluation of the potential bias of the maximum type I error rate

B.1 Simplified adaptation Rule for $L1$, $L3$ and $L4$

We recall the notation in section 3.1 and equation 8 from the manuscript:

$$\widehat{E}_L = \sum_{\text{unique set } s^*} \frac{|\mathcal{S}_{L,s^*}|}{N_{\text{sim}}} \cdot \max(R_{\mathcal{S}_{L,s^*}}(1), R_{\mathcal{S}_{L,s^*}}(2)), \tag{1}$$

The type I error rate is thus maximised when for the set of leaked data $s*$, the experimenter decides to stop at analysis 1, if $R_{\mathcal{S}_{L,s^*}}(1) > R_{\mathcal{S}_{L,s^*}}(2)$ but decides to wait until analysis 2, if $R_{\mathcal{S}_{L,s^*}}(1) < R_{\mathcal{S}_{L,s^*}}(2)$. Thus, the key is to find for each pair set of leaked data $s*$ whether $R_{\mathcal{S}_{L,s^*}}(1)$ is smaller or greater than $R_{\mathcal{S}_{L,s^*}}(2)$ and assign the corresponding decision to each set to either stop at analysis $t = 1$ or wait until analysis $t = 2$. If $R_{\mathcal{S}_{L,s^*}}(1) = R_{\mathcal{S}_{L,s^*}}(2)$, the same type I error rate is obtained whether the experimenter stops at analysis $t = 1$ or waits until analysis $t = 2$. For the simplified adaptation rule, experimenters will wait until analysis $t = 2$ if equal error rates are obtained, i.e. $R_{\mathcal{S}_{L,s^*}}(1) \leq R_{\mathcal{S}_{L,s^*}}(2)$. Nevertheless, we omitted equal cases for the smoothing algorithm and determination of the linear discriminant analysis below in order to obtain a better separation. The rule will then include equal cases to the decision of waiting until analysis $t = 2$.

In section B.3, the estimated conditional error rates $R_{\mathcal{S}_{L,s^*}}(1)$ and $R_{\mathcal{S}_{L,s^*}}(2)$ at analysis $t = 1$ and $t = 2$ for each set of leaked data $s*$ are displayed in tiles. Row grids show the delay of treatment 2 to the platform. A delay of 0 patients denotes a fixed trial (i.e., all arms start at the same time), while a delay of 70 patients denotes a trial, where already 70 patients in the control arm are on the trial (i.e. only 30 control patients will be shared with treatment 1).

Using the colour separation of the tiles, we can determine a simplified adaptation rule for each set of leaked data and evaluate the bias for the estimated maximum type I error rate in an independent simulation: Experimenter's always stop for an analysis $t = 1$ when sets are within the blue tiles, but continue to analysis $t = 2$ when sets are within red tiles. In the following, we describe our approach to determine the simplified adaptation rule by using a smoothing algorithm and a linear discriminant analysis (LDA) afterwards.

B.1.1 Smoothing algorithm

As each tile depends on the number of occurrences $|\mathcal{S}_{L,s^*}|$ of the respective set of leaked data in the simulation, some pairs were obtained more frequently than others. Some pairs were observed only in a small number of simulations. Therefore, the tile graphs are subject to noise. In order to reduce this, we performed a nearest neighbour smoothing algorithm. For each set of leaked data $s*$:

- Denote the decision indicator $DI_{L,s*}$:
  - $DI_{L,s*} = 1$, if $R_{\mathcal{S}_{L,s^*}}(1) > R_{\mathcal{S}_{L,s^*}}(2)$, implying to stop at analysis $t = 1$,
  - $DI_{L,s*} = 0$, if $R_{\mathcal{S}_{L,s^*}}(1) \leq R_{\mathcal{S}_{L,s^*}}(2)$, implying to stop at analysis $t = 2$.
- Obtain the decision indicator of the surrounding 8 neighbours of the set $s*$ and calculate the mean $\bar{DI}_{L,s*}$ using the sets indicator and the 8 neighbours.
  - For example, assume $s* = (d_2(1), d_{0_{[2]}}(1)) = (20, 15)$ for $L1$. The 8 surrounding neighbours are $(19, 14), (19, 15), (19, 16), (20, 14), (20, 16), (21, 14), (21, 15), (21, 16)$. The mean $\bar{DI}_{L,s*}$ will be calculated using the decision indicator from the 8 neighbours and from $(20, 15)$ itself.
  - If some neighbours are not available, then this will be treated as missing and less than 8 neighbours will be used to calculate $\bar{DI}_{L,s*}$.
- Create a smoothed decision indicator $DI^{smooth}_{L,s*}$.
  - If $\bar{DI}_{L,s*} \geq 0.5$, then assign $DI^{smooth}_{L,s*} = 1$, otherwise $DI^{smooth}_{L,s*} = 0$

An overlay of the smoothed tiles using the smoothed decision indicator is displayed in section B.4. As the original tiles have already shown a good clear separation of the blue and red tiles, only a few tiles were re-assigned.

B.1.2 Linear discriminant analysis

As some pairs of events have not been observed in the simulation and are therefore not seen in the tile graphs in section B.3 or section B.4, we performed a linear discriminant analysis on all available smoothed pairs in order to obtain a general adaptation rule for all pairs as follows:

- Run an LDA using the sets of leaked data $s*$ to predict a posterior probability $p_{L,s*}$ of each pair of events belonging to the class of $DI^{smooth}_{L,s*} = 1$, implying to stop at analysis $t = 1$
- Calculate Youden's criteria (Sensitivity + Specificity - 1) varying the cutoff $p_{L,s*} > c, c \in (0.1, 0.2, 0.3, 0.4, 0.5)$ and chose the cutoff $c_{L,Youden}$ which maximises Youden's criteria
- Obtain the predicted probability $p_{L,s*}$ for all sets of leaked data
- Create a LDA decision indicator $DI^{LDA}_{L,s*}$.
  - If $p_{L,s*} \geq c_{L,Youden}$, then assign $DI^{LDA}_{L,s*} = 1$ (implying to stop at analysis $t = 1$),
  - otherwise $DI^{LDA}_{L,s*} = 0$ (implying to stop at analysis $t = 2$)

An overlay of the resulting LDA predictions on the smoothed tiles is displayed in section B.5. For the majority of scenarios, the LDA provides a good separation. Nevertheless, when the amount of information is low, the separation can become difficult.

The resulting LDA is the basis for the simplified adaptation rule for $L1$, $L3$ and $L4$ when running a new simulation. Our approach to determine an adaptation rule did not aim to provide an optimal separation. To determine the optimal separation is out of scope here and would require further extensive simulations. With our simplified adaptation rule, we will approximate the estimated maximum type I error rate using the approximate adaptation rule as best as possible and determine the potential bias observed in our simulation while acknowledging the shortcomings of the approach.

B.2 Simplified adaptation Rule for $L2$

For $L2$, we notice that a pattern can be seen that distinguishes cases of $R_{\mathcal{S}_{L2,s^*}}(1) > R_{\mathcal{S}_{L2,s^*}}(2)$ from the rest which is dependent on the number of events and the log hazard ratio. Mostly, these cases can be identified if $\left(-\sqrt{d_{0_{[2]}}(1) + d_2(1)} \cdot 0.5 \cdot \log(\widehat{\theta}_2(1))\right)^2 > \chi^2_{1,0.95}$ and $\widehat{\theta}_2(1) < 1$. This is the approximation of the log-rank test statistic through the hazard ratio and number of events. Consequently, the adaptation rule leading to a separation is obtained when experimenters decide to stop for an ad-hoc analysis if the released information allows a rejection of the null hypothesis. Thus, for $L2$ no smoothing and no LDA has to be performed but the approximation of the log-rank test statistic can be used to provide an adaptation rule in a new simulation. In section B.6, alternative bin borders were chosen such that for a given number of events and the borders, the log-rank test statistic approximation would be greater than the chi-squared critical value $\chi^2_{1,1-5\%}$.

B.3 Tile graphs showing conditional error rates

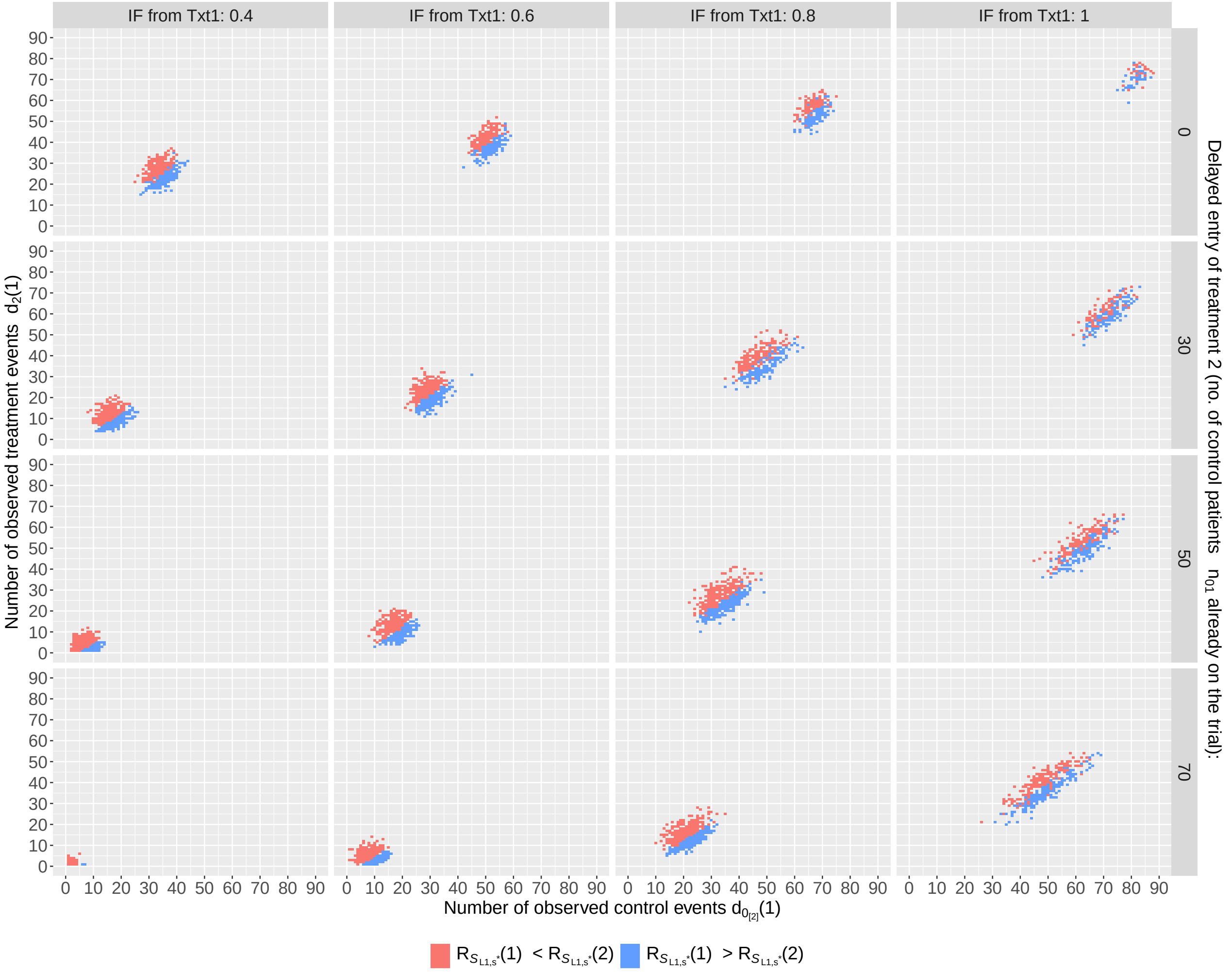

Supplementary Figure 13: $L1$: Observed events per Arm. Tile graph comparing the conditional type I error rate at the ad-hoc analysis $t = 1$ and the primary analysis $t = 2$ given the observed number of events per arm. Row grids: Delay of treatment 2 joining the platform after 0 patients (100 % of control patients), 30, 50 and 70 patients in the control arm already on the trial. Column grids: Ad-hoc analysis $t = 1$ is triggered by treatment 1 against the shared control at information fraction 0.4, 0.6, 0.8 and 1 (primary analysis). Cases of equal conditional type I error rates are omitted.

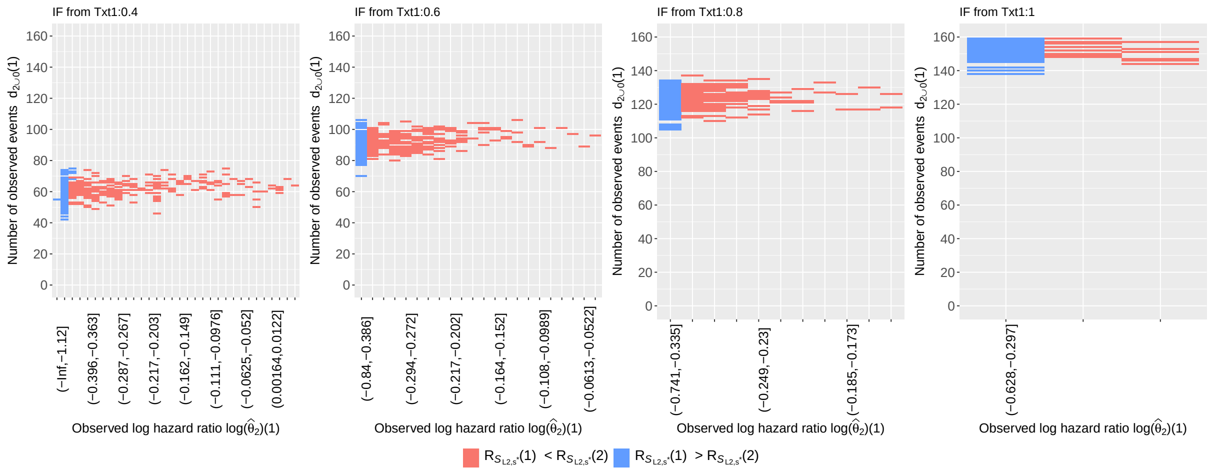

(a) Delay of treatment 2 joining the platform after 0 patients

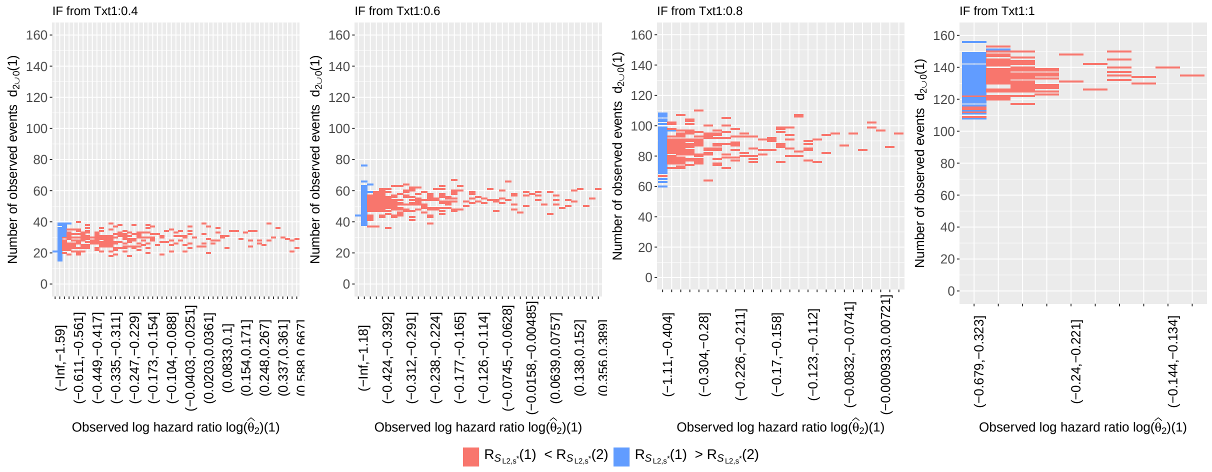

(b) Delay of treatment 2 joining the platform after 30 patients

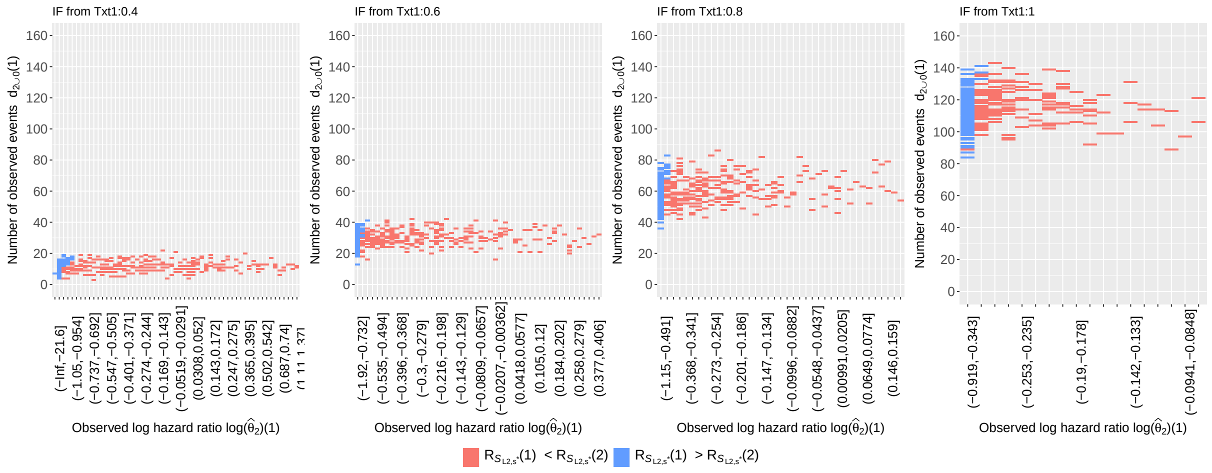

(c) Delay of treatment 2 joining the platform after 50 patients

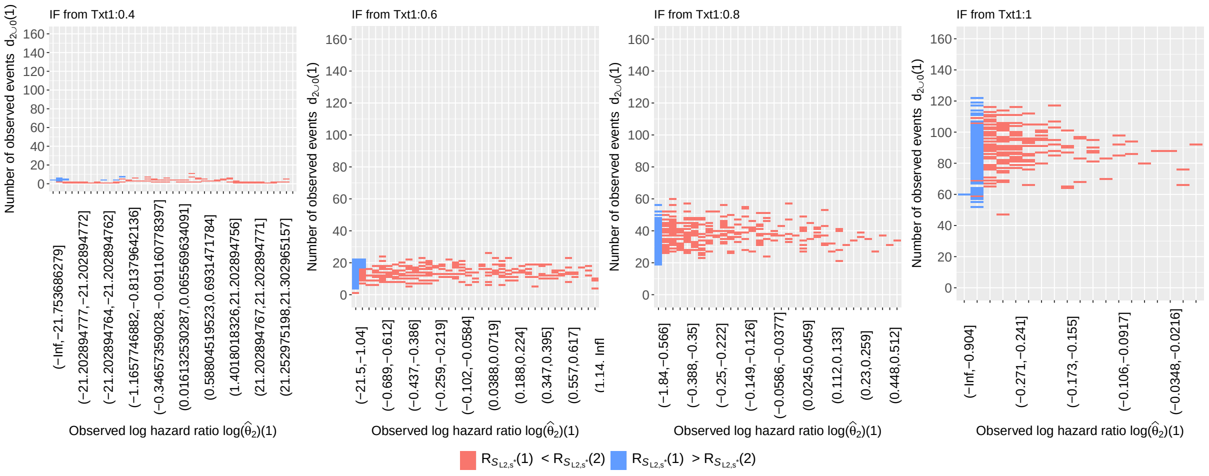

(d) Delay of treatment 2 joining the platform after 70 patients

Supplementary Figure 14: *L*2: Observed Hazard Ratio and Observed Pooled events. Tile graph comparing the estimated conditional type I error rate at the ad-hoc analysis $t = 1$ and the primary analysis $t = 2$ given the observed number of events and observed log hazard ratio. Ad-hoc analysis $t = 1$ is triggered by treatment 1 against the shared control at information fraction 0.4, 0.6, 0.8 and 1 (primary analysis). Cases of equal conditional type I error rates are omitted.

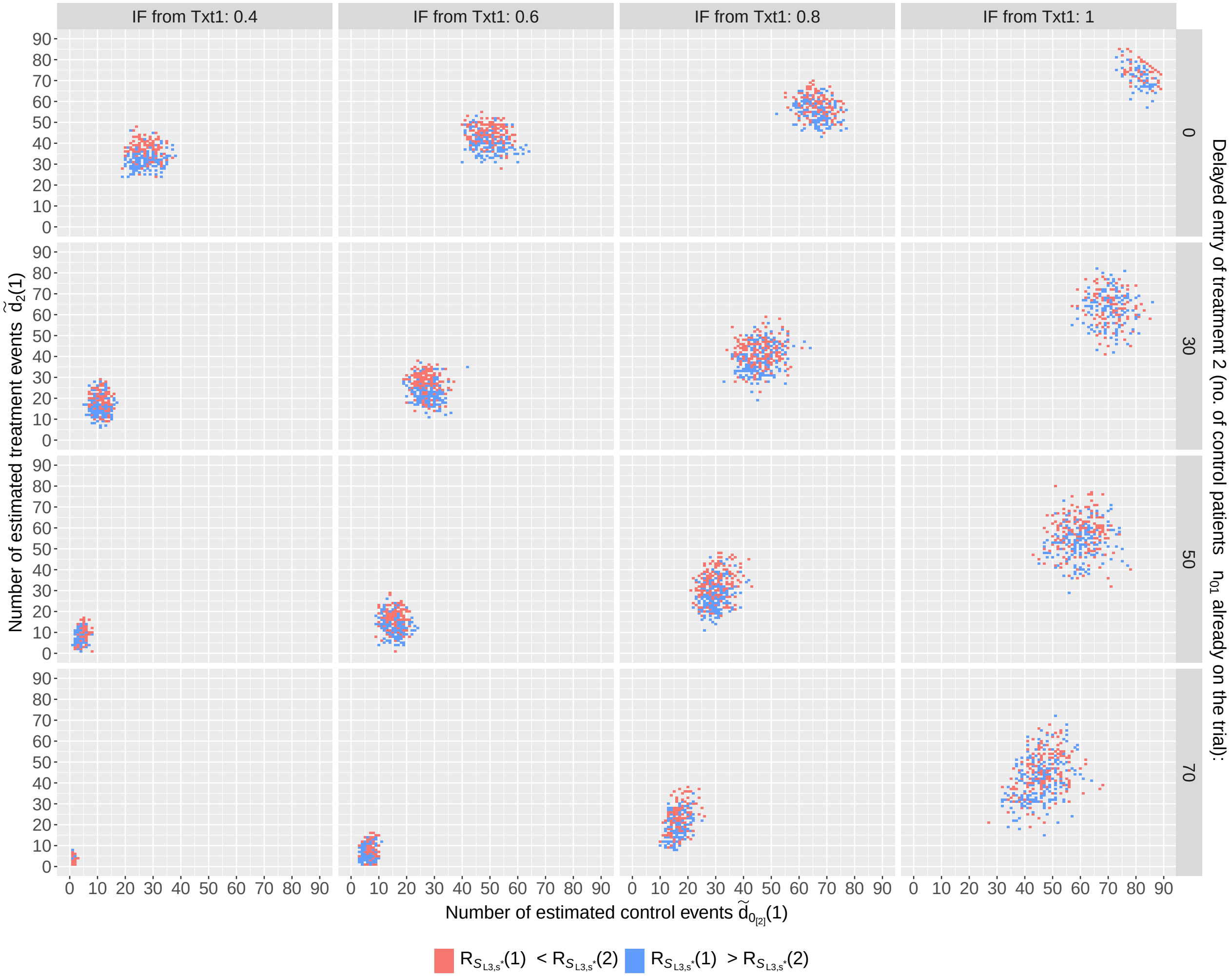

Supplementary Figure 15: $L3$: Estimated Events per Arm. Tile graph comparing the estimated conditional type I error rate at the ad-hoc analysis $t = 1$ and the primary analysis $t = 2$ given the observed number of events per arm. Row grids: Delay of treatment 2 joining the platform after 0 patients (100 % of control patients), 30, 50 and 70 patients in the control arm already on the trial. Column grids: Ad-hoc analysis $t = 1$ is triggered by treatment 1 against the shared control at information fraction 0.4, 0.6, 0.8 and 1 (primary analysis). Cases of equal conditional type I error rates are omitted.

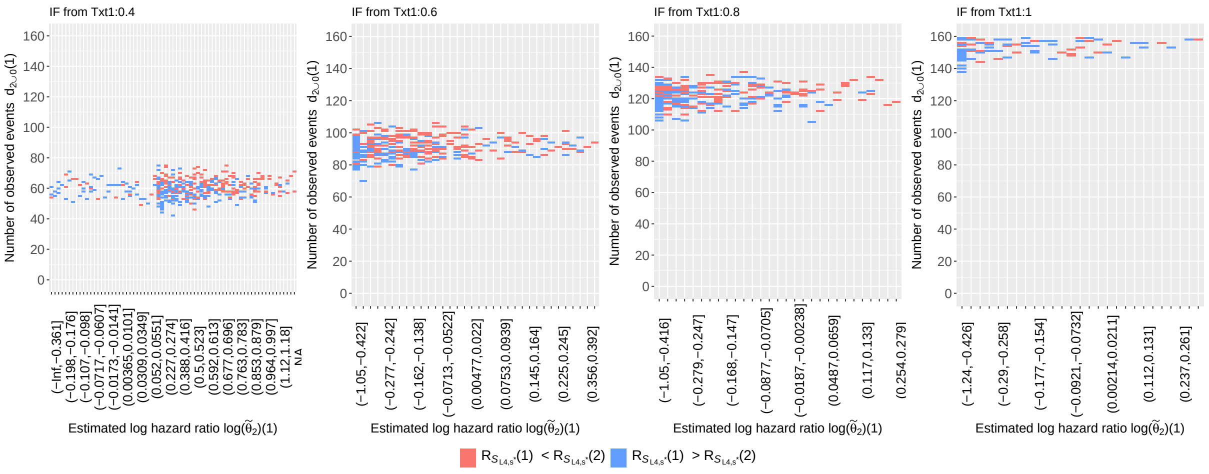

(a) Delay of treatment 2 joining the platform after 0 patients

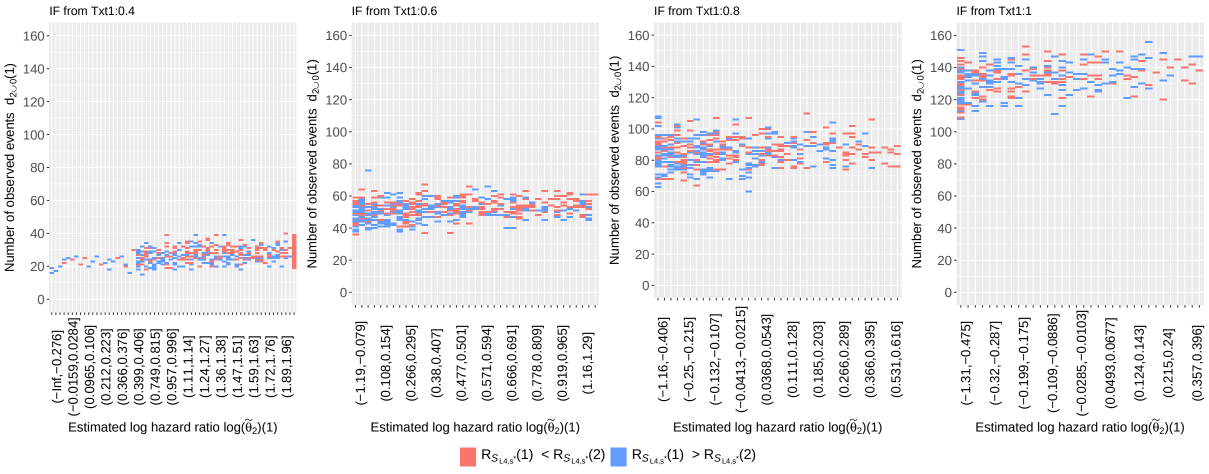

(b) Delay of treatment 2 joining the platform after 30 patients

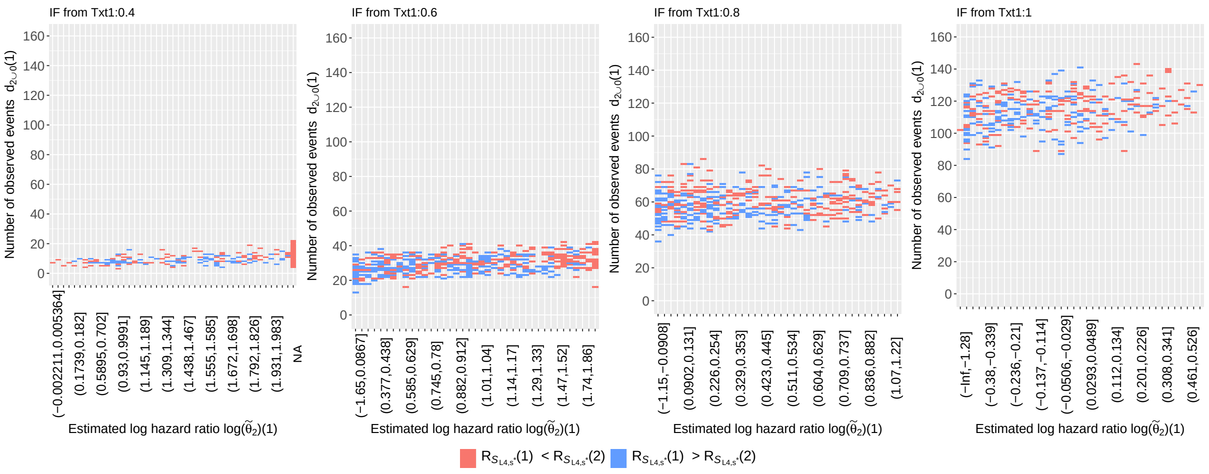

(c) Delay of treatment 2 joining the platform after 50 patients

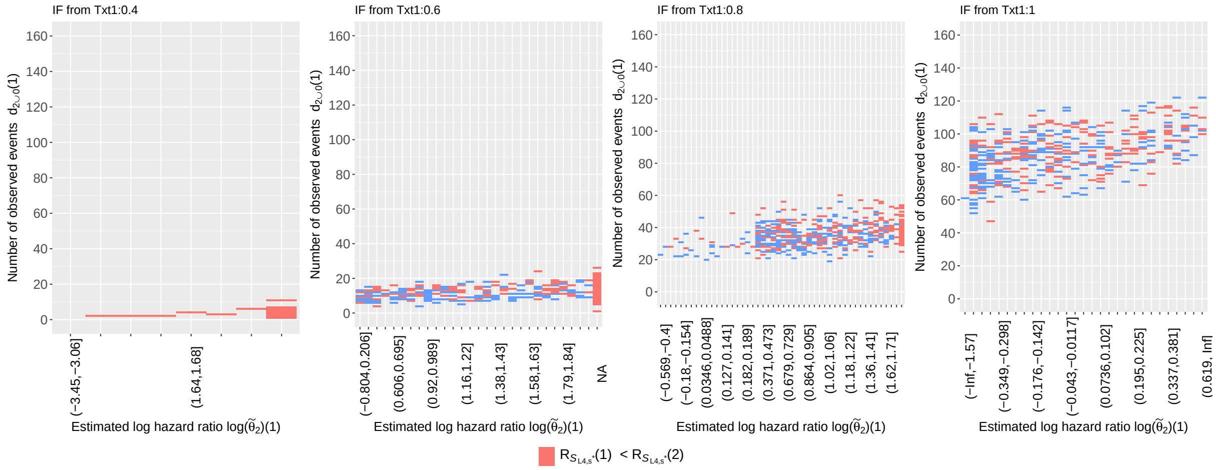

(d) Delay of treatment 2 joining the platform after 70 patients

Supplementary Figure 16: $L4$: Estimated Hazard Ratio and Observed Pooled events. Tile graph comparing the estimated conditional type I error rate at the ad-hoc analysis $t = 1$ and the primary analysis $t = 2$ given the observed number of events and estimated log hazard ratio. Ad-hoc analysis $t = 1$ is triggered by treatment 1 against the shared control at information fraction 0.4, 0.6, 0.8 and 1 (primary analysis). Cases of equal conditional type I error rates are omitted.

B.4 Smoothed tile graphs

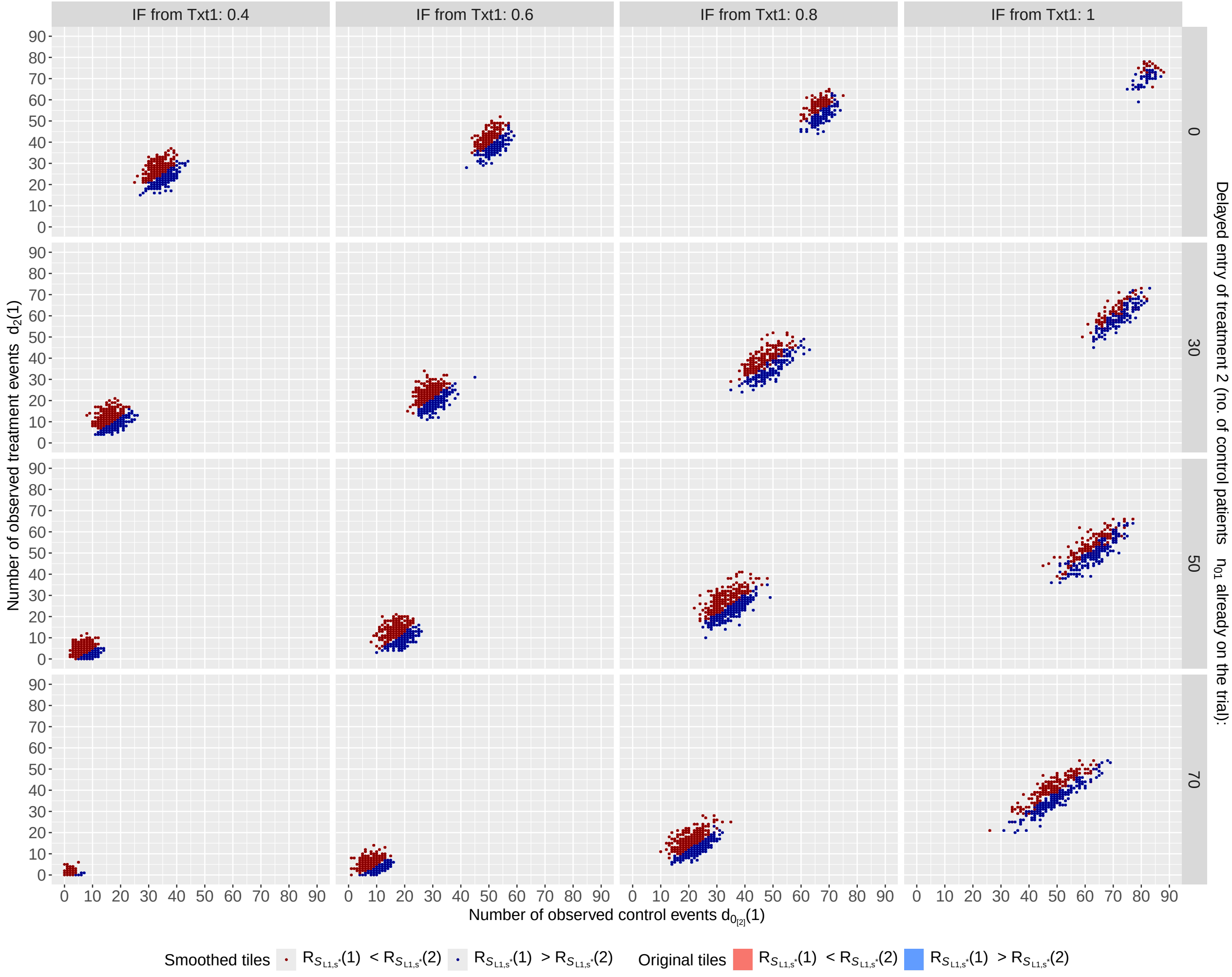

Supplementary Figure 17: $L1$: Observed events per Arm. Smoothed tile graph comparing the estimated conditional type I error rate at the ad-hoc analysis $t = 1$ and the primary analysis $t = 2$ given the observed number of events per arm. Tiles represent the original tiles. Dots are overlayed showing the smoothed tiles using the smoothed decision indicator. Row grids: Delay of treatment 2 joining the platform after 0 patients (100 % of control patients), 30, 50 and 70 patients in the control arm already on the trial. Column grids: Ad-hoc analysis $t = 1$ is triggered by treatment 1 against the shared control at information fraction 0.4, 0.6, 0.8 and 1 (primary analysis). Cases of equal conditional type I error rates are omitted.

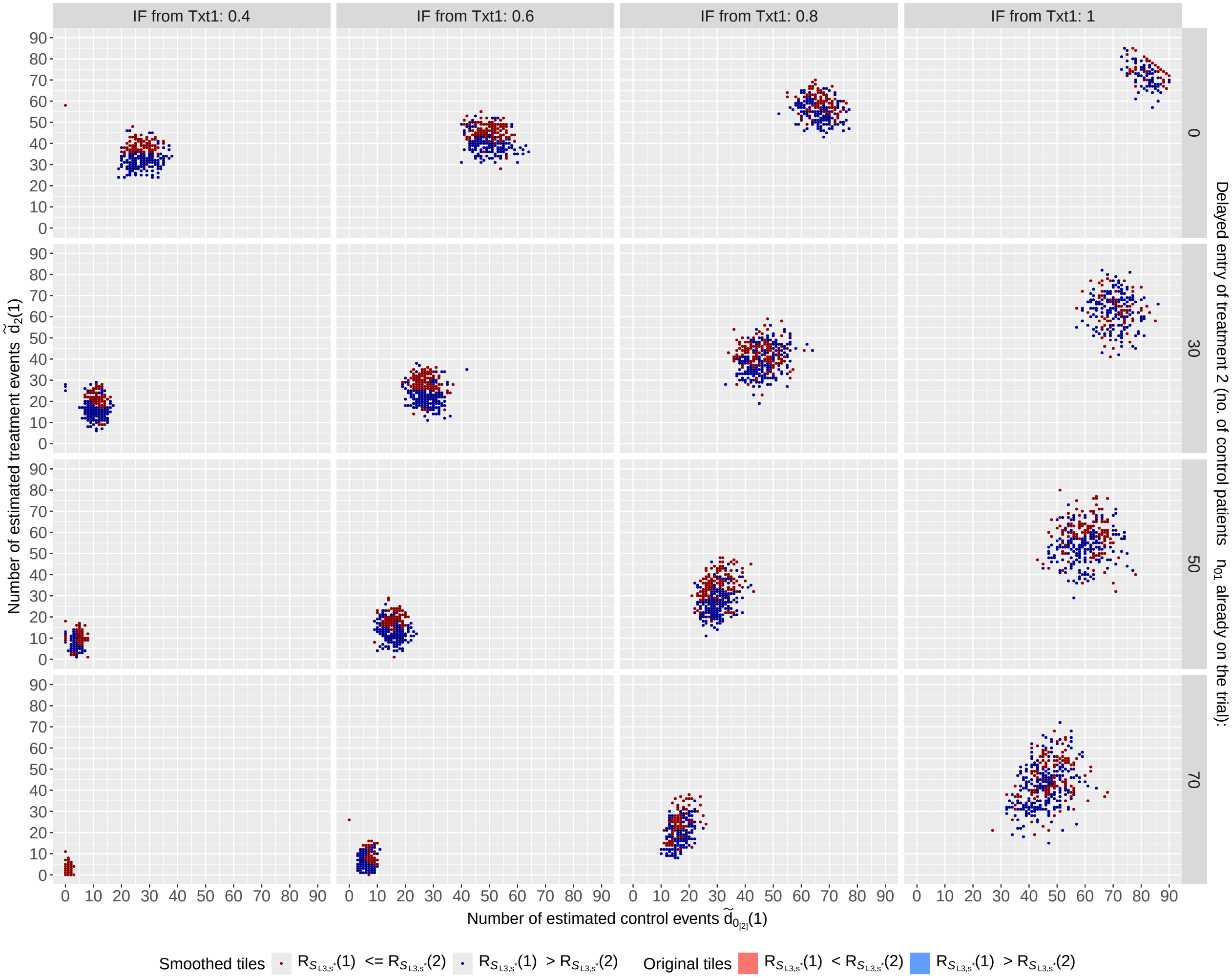

Supplementary Figure 18: $L3$: Estimated events per Arm. Smoothed tile graph comparing the estimated conditional type I error rate at the ad-hoc analysis $t = 1$ and the primary analysis $t = 2$ given the estimated number of events per arm. Tiles represent the original tiles. Dots are overlayed showing the smoothed tiles using the smoothed decision indicator. Row grids: Delay of treatment 2 joining the platform after 0 patients (100 % of control patients), 30, 50 and 70 patients in the control arm already on the trial. Column grids: Ad-hoc analysis $t = 1$ is triggered by treatment 1 against the shared control at information fraction 0.4, 0.6, 0.8 and 1 (primary analysis). Cases of equal conditional type I error rates are omitted.

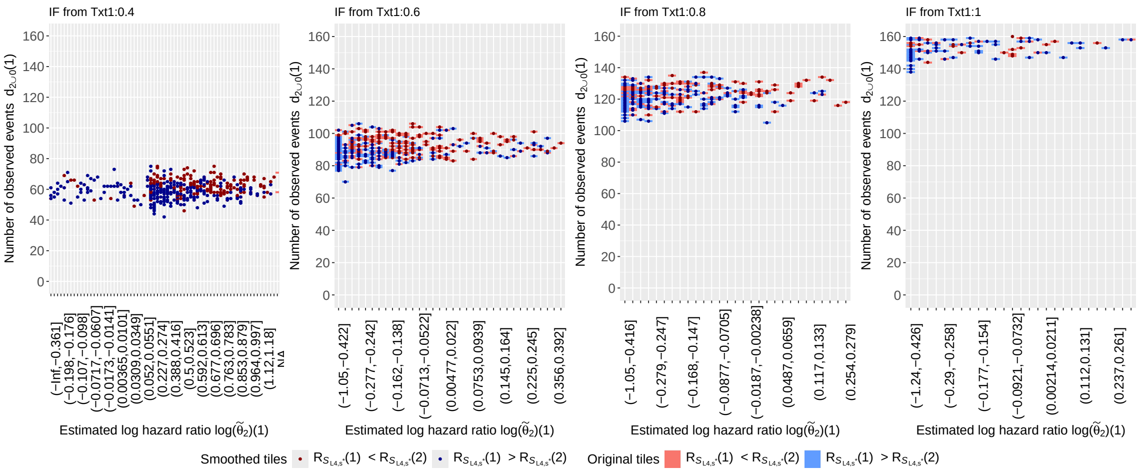

(a) Delay of treatment 2 joining the platform after 0 patients

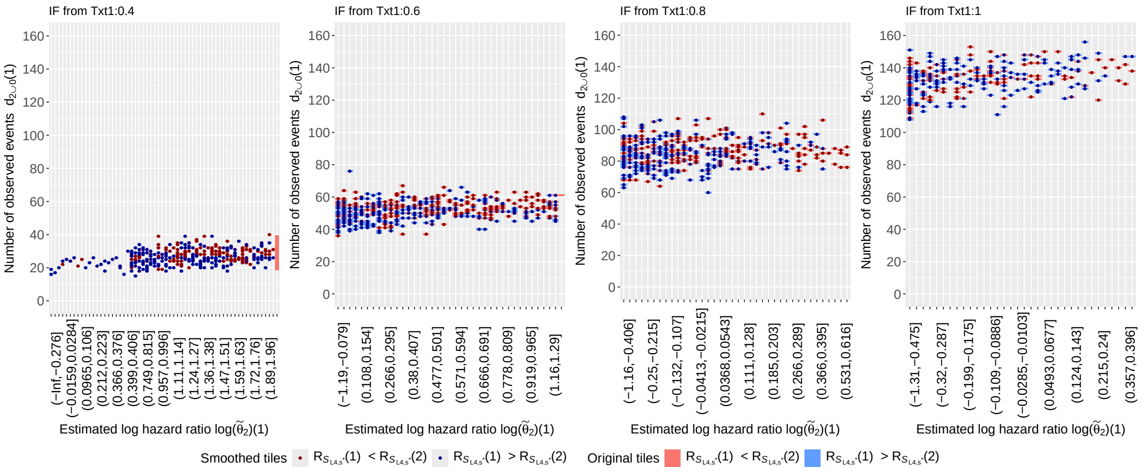

(b) Delay of treatment 2 joining the platform after 30 patients

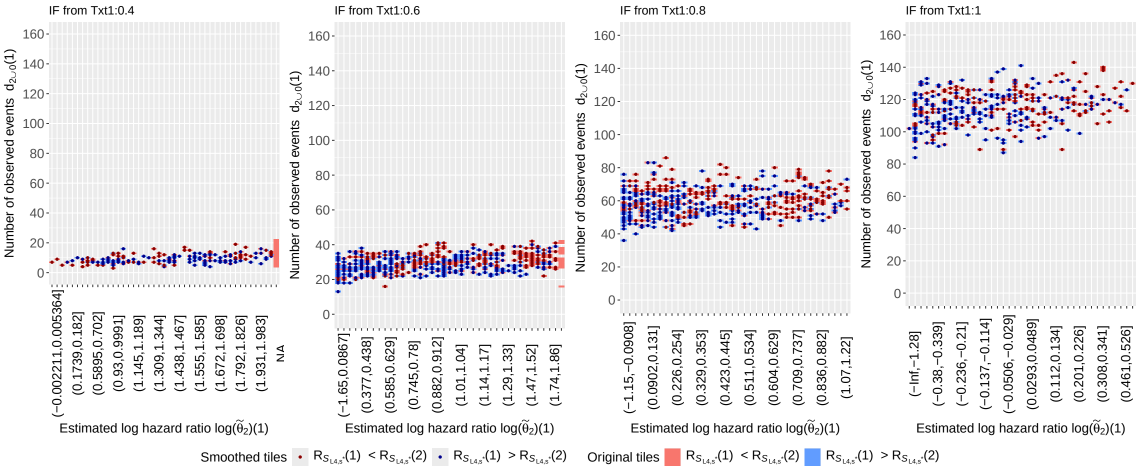

(c) Delay of treatment 2 joining the platform after 50 patients

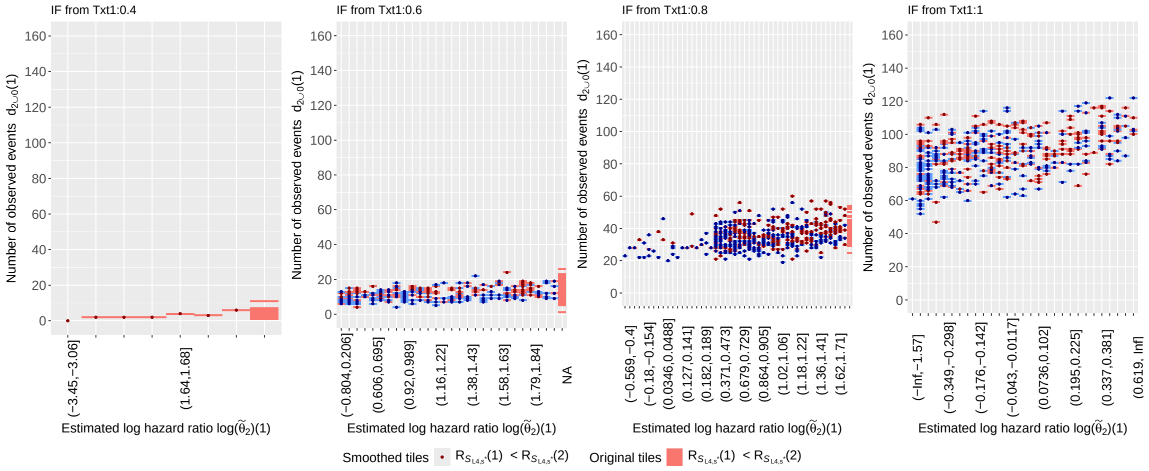

(d) Delay of treatment 2 joining the platform after 70 patients

Supplementary Figure 19: *L*4: Estimated Hazard Ratio and Observed Pooled events. Smoothed tile graph comparing the estimated conditional type I error rate at the ad-hoc analysis $t = 1$ and the primary analysis $t = 2$ given the observed number of events and estimated log hazard ratio. Tiles represent the original tiles. Dots are overlayed showing the smoothed tiles using the smoothed decision indicator. Ad-hoc analysis $t = 1$ is triggered by treatment 1 against the shared control at information fraction 0.4, 0.6, 0.8 and 1 (primary analysis). Cases of equal conditional type I error rates are omitted.

B.5 LDA overlay on tile graphs

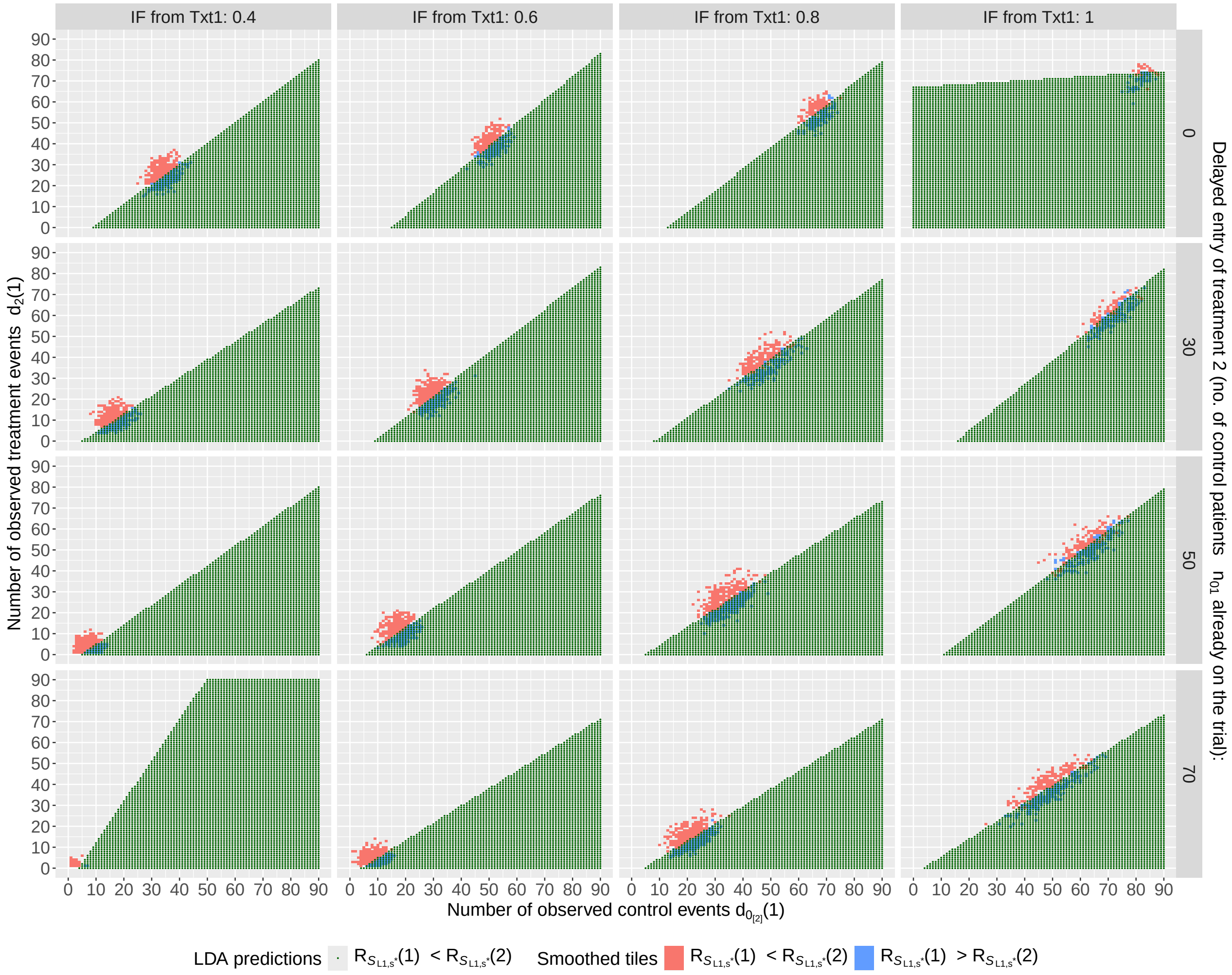

Supplementary Figure 20: $L1$: Observed events per Arm. Smoothed tile graph comparing the estimated conditional type I error rate at the ad-hoc analysis $t = 1$ and the primary analysis $t = 2$ given the observed number of events per arm. Tiles represent the smoothed tiles. Dots are overlayed showing the LDA predictions. Row grids: Delay of treatment 2 joining the platform after 0 patients (100 % of control patients), 30, 50 and 70 patients in the control arm already on the trial. Column grids: Ad-hoc analysis $t = 1$ is triggered by treatment 1 against the shared control at information fraction 0.4, 0.6, 0.8 and 1 (primary analysis). Cases of equal conditional type I error rates are omitted.

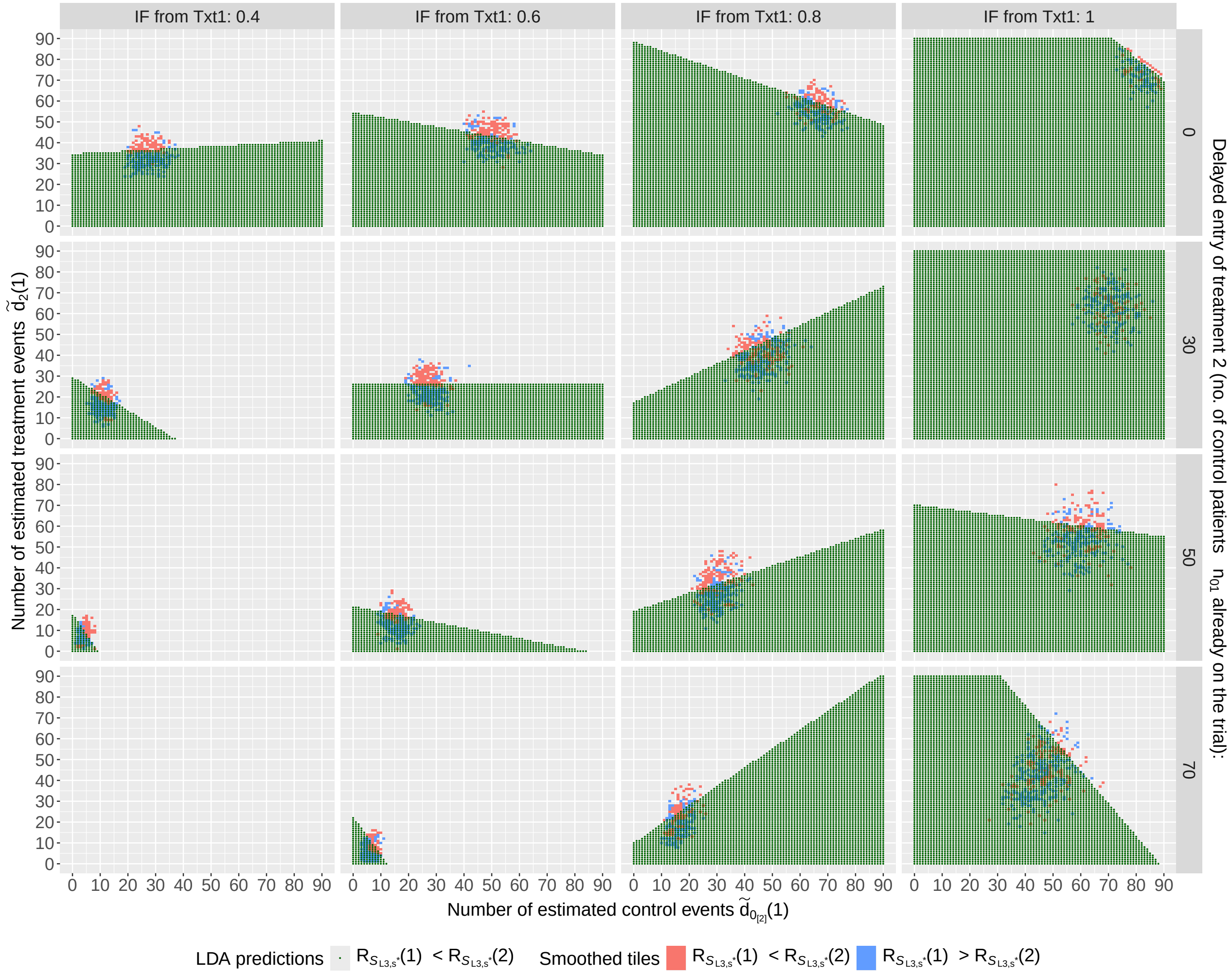

Supplementary Figure 21: *L*3: Estimated events per Arm. Smoothed tile graph comparing the estimated conditional type I error rate at the ad-hoc analysis $t = 1$ and the primary analysis $t = 2$ given the estimated number of events per arm. Tiles represent the smoothed tiles. Dots are overlayed showing the LDA predictions. Row grids: Delay of treatment 2 joining the platform after 0 patients (100 % of control patients), 30, 50 and 70 patients in the control arm already on the trial. Column grids: Ad-hoc analysis $t = 1$ is triggered by treatment 1 against the shared control at information fraction 0.4, 0.6, 0.8 and 1 (primary analysis). Cases of equal conditional type I error rates are omitted.

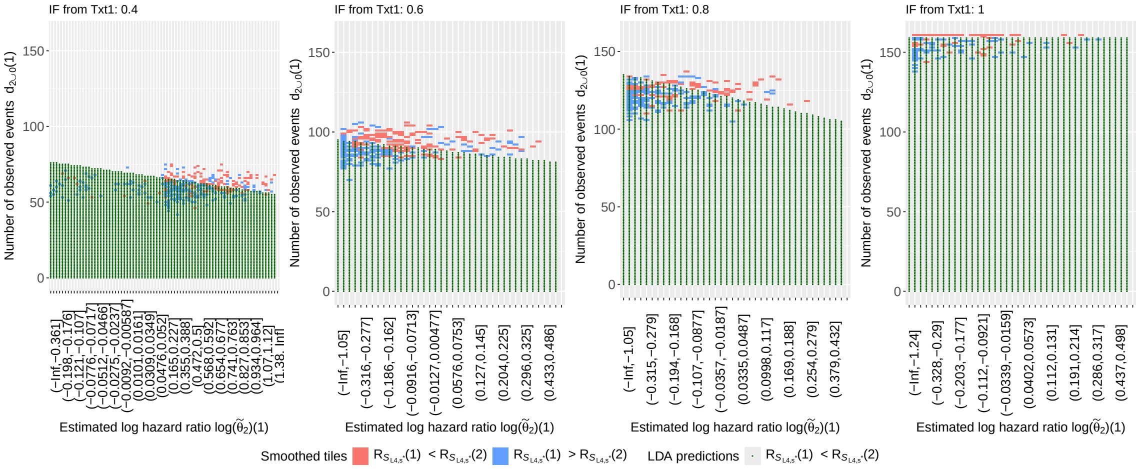

(a) Delay of treatment 2 joining the platform after 0 patients

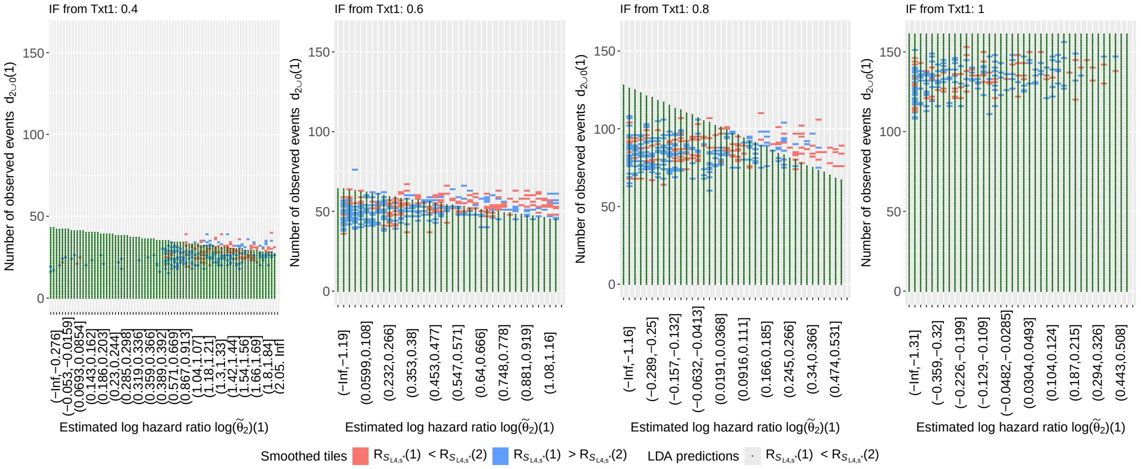

(b) Delay of treatment 2 joining the platform after 30 patients

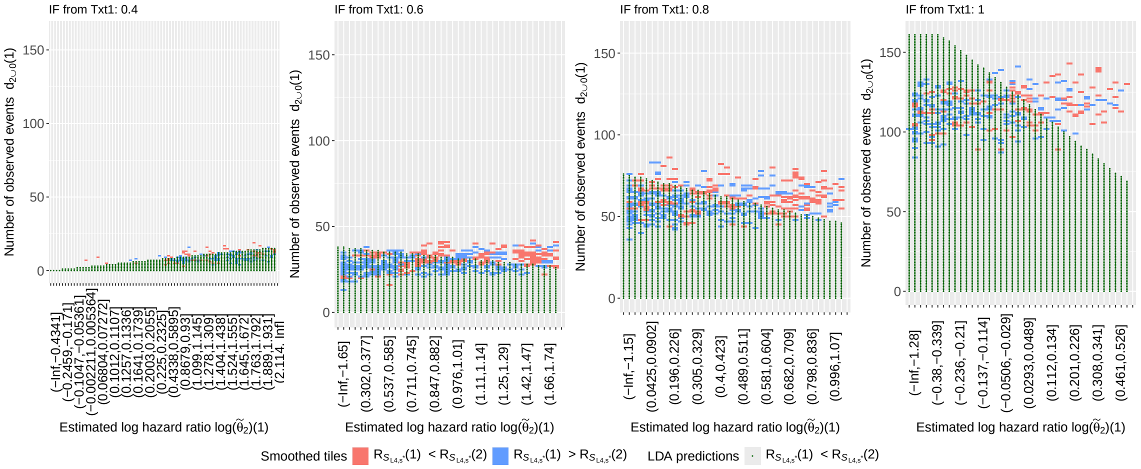

(c) Delay of treatment 2 joining the platform after 50 patients

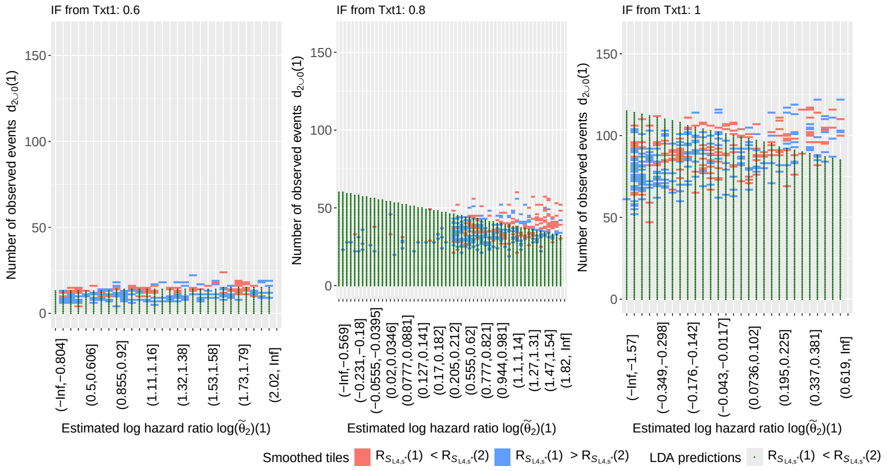

(d) Delay of treatment 2 joining the platform after 70 patients

Supplementary Figure 22: *L*4: Estimated Hazard Ratio and Observed Pooled events. Smoothed tile graph comparing the estimated conditional type I error rate at the ad-hoc analysis $t = 1$ and the primary analysis $t = 2$ given the observed number of events and estimated log hazard ratio. Tiles represent the smoothed tiles. Dots are overlayed showing the LDA predictions. Ad-hoc analysis $t = 1$ is triggered by treatment 1 against the shared control at information fraction 0.4, 0.6, 0.8 and 1 (primary analysis). Cases of equal conditional type I error rates are omitted.

B.6 Overlay of approximation of log-rank test statistic

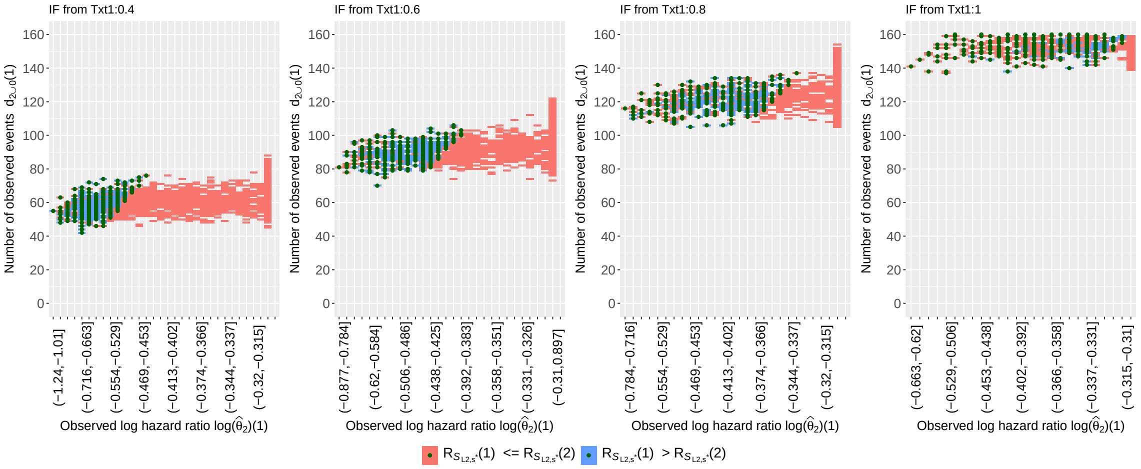

(a) Delay of treatment 2 joining the platform after 0 patients

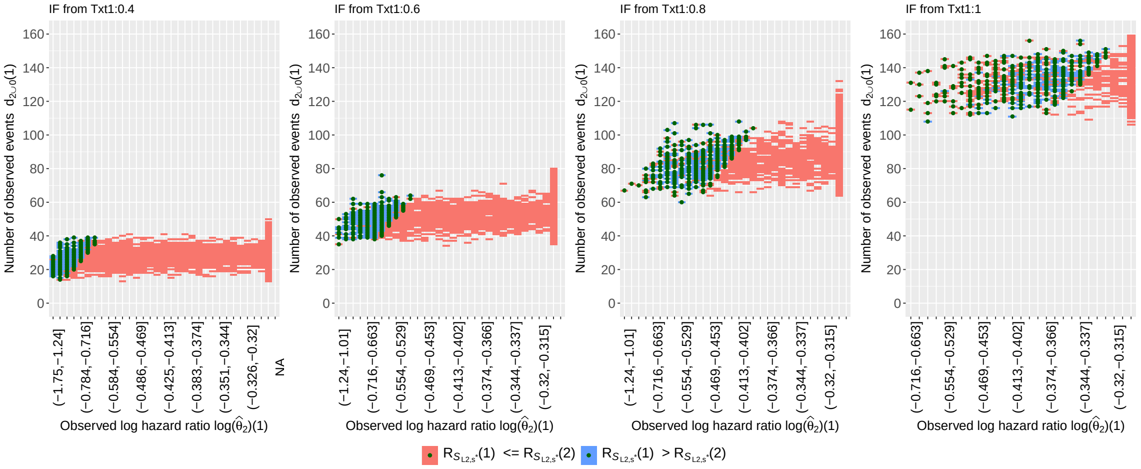

(b) Delay of treatment 2 joining the platform after 30 patients

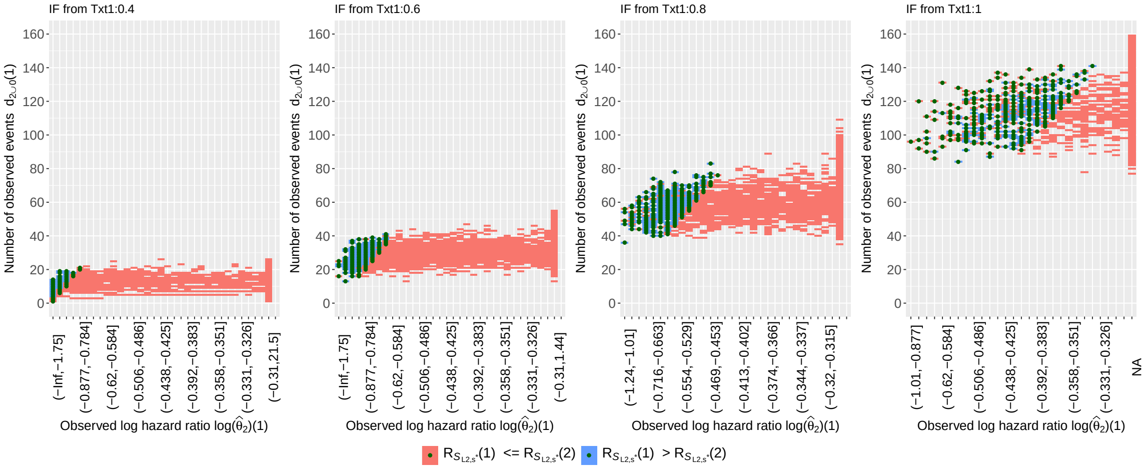

(c) Delay of treatment 2 joining the platform after 50 patients

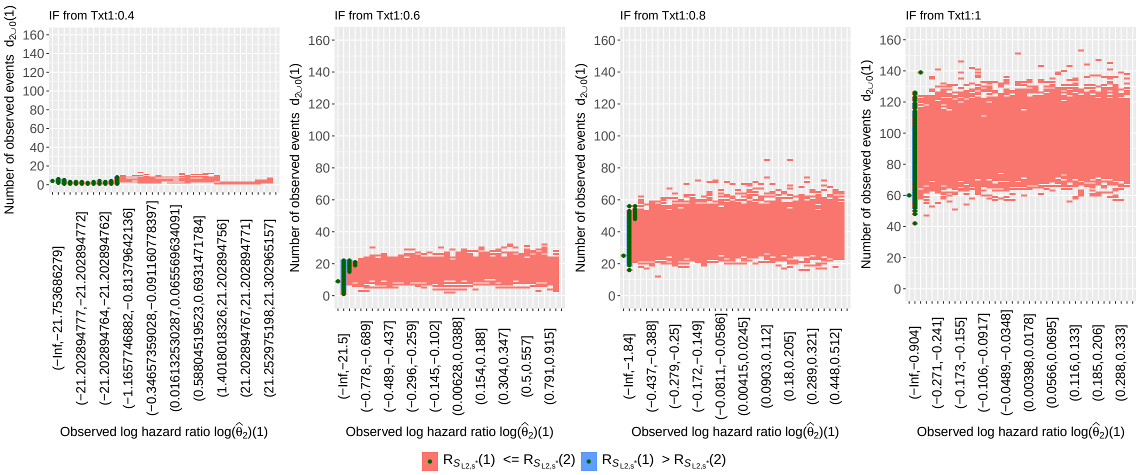

(d) Delay of treatment 2 joining the platform after 70 patients

Supplementary Figure 23: *L*2: Observed Hazard Ratio and Observed Pooled events. Tile graph comparing the estimated conditional type I error rate at the ad-hoc analysis $t = 1$ and the primary analysis $t = 2$ given the observed number of events and observed log hazard ratio. Dots are overlayed showing the condition $(-\sqrt{d_{0_{[2]}}(1) + d_2(1)} \times 0.5 \times log(\widehat{\theta_2(1)}))^2 > \chi^2_{1,1-5\%}$ and $\widehat{\theta_2(1)} < 1$. Ad-hoc analysis $t = 1$ is triggered by treatment 1 against the shared control at information fraction 0.4, 0.6, 0.8 and 1 (primary analysis). Cases of equal conditional type I error rates are omitted.

B.7 Impact of the categorisation of the HR on the maximum type I error rate for $L2$

Overlay of the estimated maximum type I error rate using the approximate adaptation rule for $L2$ with an alternative definition of the bin borders (orange line) for the hazard ratio as defined in section B.2. The alternative bin borders were chosen such that for a given number of events (y-axis) and the chosen hazard ratio bin border, the log-rank test statistic would be greater than the chi-squared critical value, i.e., leading to a rejection. The bin borders used throughout the manuscript for $L2$ using the approximate adaptation rule were based on quantiles (blue line).

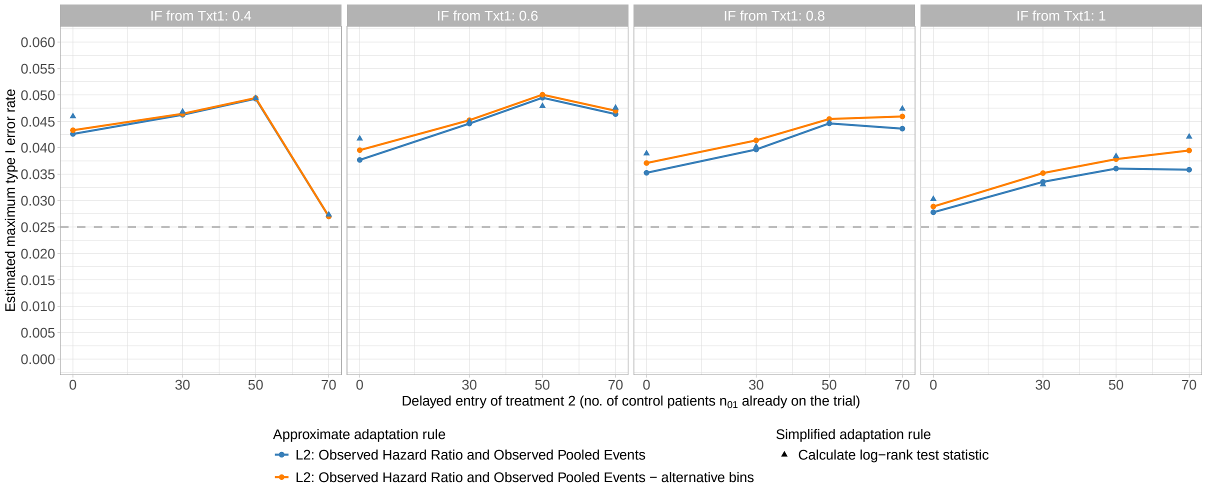

Supplementary Figure 24: Estimated maximum type I error rate for the comparison of treatment 2 and the control using the approximate adaptation rule (Eq. (10)) of $L2$ with the categorisation of the HR according to quantiles or alternatively when results of treatment 1 against the shared control at an interim analysis (information fraction (IF) 0.4, 0.6, 0.8), or at the primary analysis (information fraction 1) (see grids) of treatment 1 is published, and delayed entry of treatment 2 (x-axis). Entry at 0 denotes an immediate start of treatment 2 (i.e., all arms start at the same time), an entry at 70 denotes a late entry. An additional simulation was performed using the simplified adaptation rule using the log-rank test statistic (triangle).

C Power

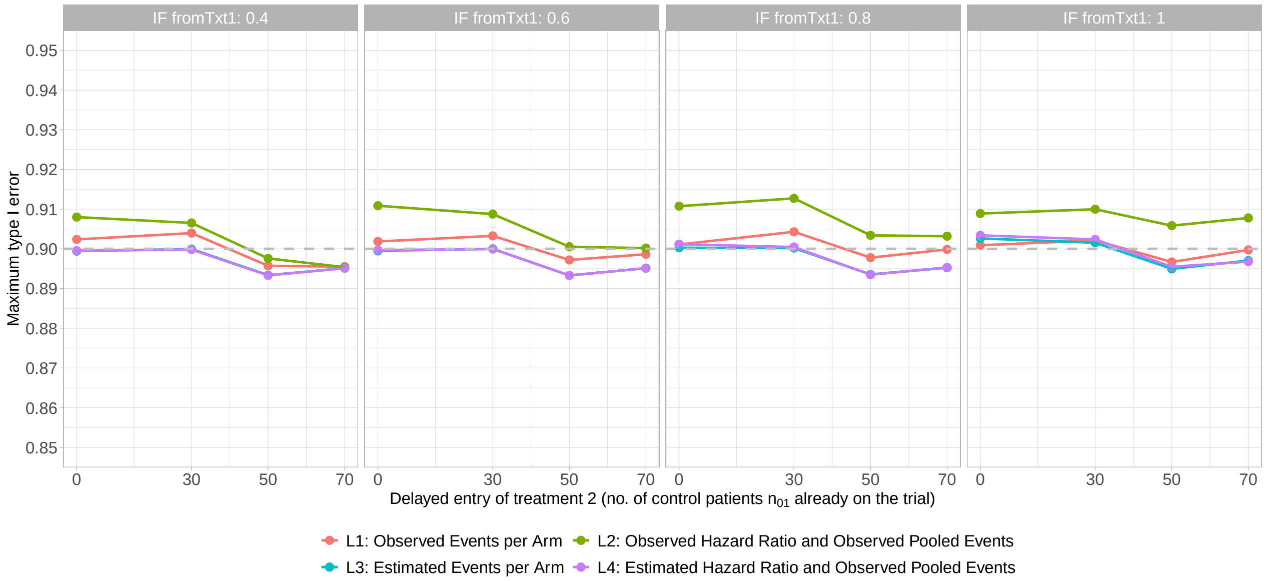

Supplementary Figure 25: Best case estimated power when analysis of treatment 1 against the shared control is published at information fraction 0.4, 0.6, 0.8 and 1 (primary analysis) (see grids) from treatment 1, and delayed entry of treatment 2 (x-axis). Entry at 0 denotes an immediate start of treatment 2, an entry at 70 denotes a late entry. Treatment 1 under null (HR 1).

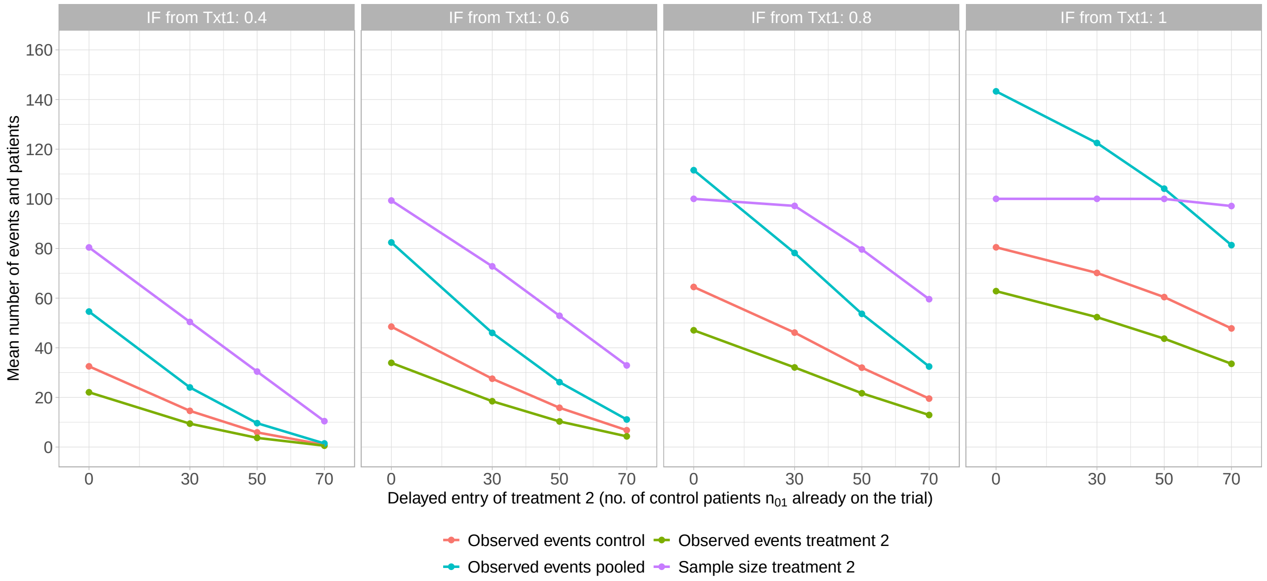

Supplementary Figure 26: Mean number of events in the control or treatment 2, as well as pooled (control and treatment 2). Mean number of patients recruited to treatment 2 at the time of analysis of treatment 1.

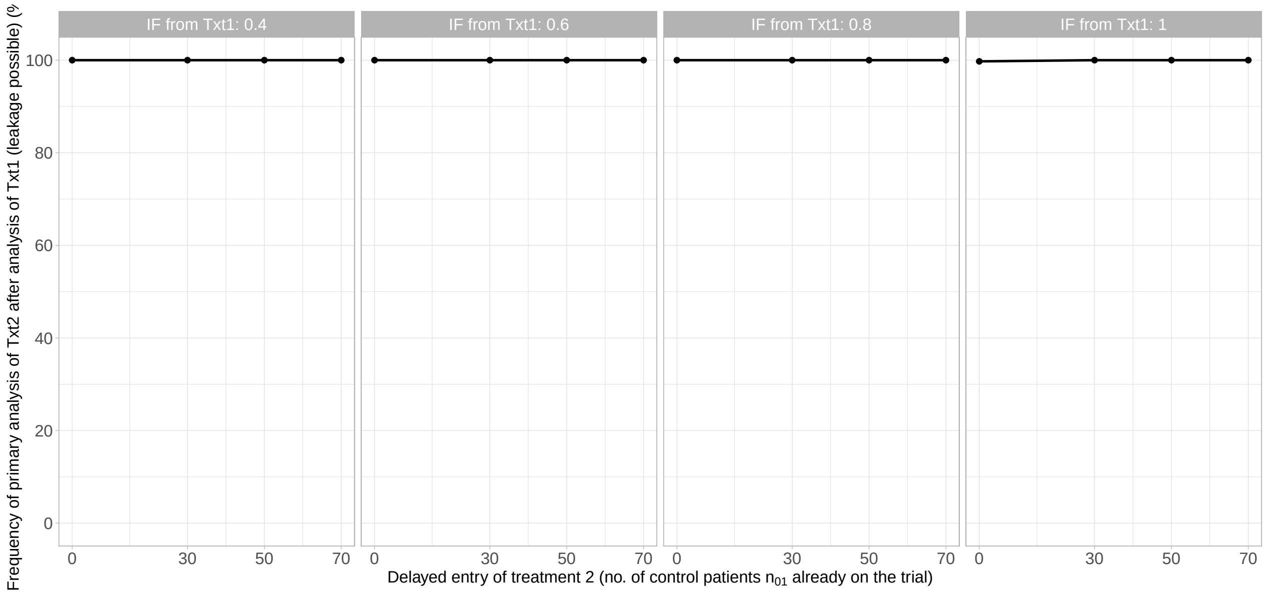

Supplementary Figure 27: Frequency of primary analysis of treatment 2 after an interim/primary analysis of treatment 1 (i.e. a possibility for leakage).

D Main simulation input parameter

Supplementary Table 1: Input parameters for the main simulation.

**Parameter**	**Treatment 1**	**Treatment 2**	**Control**
1. Data generation per exponential distribution and statistical test			
Median survival (months)	5	5	5
Target number of pooled events (treatment and control) for primary analysis	161	161	
Dropout	No dropout	No dropout	No dropout
Censoring	Administrative	Administrative	Administrative
Statistical test	Log-rank test	Log-rank test	
Significance level (one-sided)	2.5%	2.5%	
Time point of analysis (IF)	0.2, 0.4, 0.6, 0.8, 1.0	1.0	
2. All treatments start on the platform at the same time at 0 months			
Accrual start (months)	0	0	0
Accrual end (months)	10	10	10
Total sample size	100	100	100
3. Delayed entry of treatment 2 at 3 months			
Accrual start (months)	0	3	0
Accrual end (months)	10	13	13
Total sample size	100	100	130
4. Delayed entry of treatment 2 at 5 months			
Accrual start (months)	0	5	0
Accrual end (months)	10	15	15
Total sample size	100	100	150
5. Delayed entry of treatment 2 at 7 months			
Accrual start (months)	0	7	0
Accrual end (months)	10	17	17
Total sample size	100	100	170

References

Ristl, R. et al. (2021). "Delayed treatment effects, treatment switching and heterogeneous patient populations: How to design and analyze RCTs in oncology". In: *Pharmaceutical Statistics* 20(1), pp. 129–145. DOI: 10.1002/pst.2062.
